# Survey and analysis of crystal polymorphism in organic structures

**DOI:** 10.1107/S2052252518000660

**Published:** 2018-01-25

**Authors:** Kortney Kersten, Ramanpreet Kaur, Adam Matzger

**Affiliations:** a Department of Chemistry, University of Michigan, 930 North University Avenue, Ann Arbor, MI 48109, USA; b Department of Macromolecular Science And Engineering, University of Michigan, 930 North University Avenue, Ann Arbor, MI 48109, USA

**Keywords:** phase transitions, polymorphs, hydrates, cocrystals, Cambridge Structural Database

## Abstract

With the intention of producing the most comprehensive treatment of the prevalence of crystal polymorphism among structurally characterized materials, all polymorphic compounds flagged as such within the Cambridge Structural Database (CSD) are analysed and a list of crystallographically characterized organic polymorphic compounds is assembled. Classifying these structures into subclasses of anhydrates, salts, hydrates, non-hydrated solvates and cocrystals reveals that there are significant variations in polymorphism prevalence as a function of crystal type, a fact which has not previously been recognized in the literature. It is also shown that, as a percentage, polymorphic entries are decreasing temporally within the CSD, with the notable exception of cocrystals, which continue to rise at a rate that is a constant fraction of the overall entries. Some phenomena identified that require additional scrutiny include the relative prevalence of temperature-induced phase transitions among organic salts and the paucity of polymorphism in crystals with three or more chemical components.

## Abstract 2

A comprehensive list of organic crystalline polymorphs has been assembled using the Cambridge Structural Database (CSD) and the structures categorized by crystal type, uncovering significant variations in the polymorphism prevalence of each group. Phenomena such as the high prevalence of temperature-induced phase transitions in organic salts and the diminishing percentage of polymorphic crystal entries in the CSD over the last 20 years, with the exception of cocrystals, highlight areas of prospective study.

## 1. Introduction   

Polymorphism is a concept that has been well known within the crystallization field since Mitscherlich discovered different crystal forms of the same phosphate salt in the early 1800s (Bernstein, 2002 ▸). However, it was not until the mid-1960s that McCrone (1965 ▸) presented a review of the relevance of this concept in the field of pharmaceuticals, where it would eventually become one of the most studied topics in solid-state organic chemistry. McCrone (1965 ▸) famously posited that the discovery of polymorphs is correlated with the energy and time put into researching a compound. We have spent the last 15 years in our laboratory researching crystallization and polymorphism (López-Mejías *et al.*, 2011 ▸; Pfund & Matzger, 2014 ▸), and indeed, polymorphs of many molecules have been isolated in this time (López-Mejías *et al.*, 2012 ▸; Lutker & Matzger, 2010 ▸; Pfund *et al.*, 2015 ▸; Roy *et al.*, 2016 ▸; Lutker *et al.*, 2011 ▸), leading to advancements in the understanding of solid-state molecular packing and how variations in packing can affect physical properties. We are also not alone in this endeavour; a search for the term ‘polymorph’ in the journal *Crystal Growth & Design* (http://pubs.acs.org/journal/cgdefu, published by the American Chemical Society) shows that, on average, 18% of the research articles and communications published in the last 15 years contain this term. (It is notable that searching for this term in *Crystal Growth & Design* leads to very few false hits involving genetic polymorphism or indeed other meanings of the term; see section S1 of the supporting information for more details.) However, funds are limited and researcher time is in high demand, so scrutinizing every new organic molecule for polymorphism is not a realistic goal. With this obvious constraint, it is important to understand the limitations of this research topic and how to utilize what has previously been discovered in order to direct future research most efficiently (Cruz-Cabeza *et al.*, 2015 ▸). Here, we examine organic polymorphs deposited in the Cambridge Structural Database (CSD) to determine the trends in prevalence as a function of time and crystal type, thus providing an overview of research activity and progress in the field.

The CSD (Groom *et al.*, 2016 ▸) is the most extensive and accessible listing of crystal structures within the scientific community, and it serves as the basis for the analysis herein. Using this approach will underestimate the occurrence of a phenomenon such as polymorphism because many crystalline materials are not crystallographically characterized and deposited into this database. For example, Stahly (2007 ▸) showed that solid-form screening can lead to many polymorphic forms being discovered, albeit for a subset of pharma­ceutically relevant compounds whose structures were not disclosed. In order to choose a more inclusive subset of crystals, the entries available in the CSD are analysed here to make general conclusions based on the relative occurrence of polymorphism in organic compounds.

When a structure is deposited in the CSD, information about the compound is recorded such as unit-cell parameters, molecular makeup and the experimental conditions used to solve the structure. This can also include other relevant data tags such as the mention of polymorphism of the specific chemical entity. However, since the term ‘polymorphism’ is not always uniformly defined, entries are sometimes flagged as polymorphs that are not equivalent in nature (Bernstein, 2002 ▸; Bhatt & Desiraju, 2007 ▸; Rodríguez-Spong *et al.*, 2004 ▸; Grant, 1999 ▸). In addition, there is a lack of distinction between structures that represent two forms that can coexist under the same conditions, and those solid phases of a compound existing only under specific and separate conditions. Practically, this relationship is important because the stability of a form directly relates to its properties, such as bioavailability in pharmaceuticals or performance in energetic materials (Karpinski, 2006 ▸; Borka & Haleblian, 1990 ▸; Bolton & Matzger, 2011 ▸; Kersten & Matzger, 2016 ▸; Bauer *et al.*, 2001 ▸). For this reason, attempts are made herein to distinguish between these types of polymorph in the CSD.

Building on past efforts involving surveys of subsets of the CSD (Cruz-Cabeza *et al.*, 2015 ▸; Bernstein, 2002 ▸; van de Streek & Motherwell, 2005 ▸; Sarma & Desiraju, 1999 ▸), we sought here to be more comprehensive and inclusive in this analysis such that a number of trends can be discerned; these trends may be considered as one piece of the puzzle that is crystal polymorphism. Sarma & Desiraju (1999 ▸) conducted a seminal study of polymorphism prevalence, where both organic and organometallic single-component polymorphs from the CSD were analysed based on carbon content and molecular flexibility. Overall, they concluded polymorphism to be ‘essentially a random phenomenon’, with molecules of all sizes showing the same prevalence for polymorphism at ∼3% (Sarma & Desiraju, 1999 ▸). Cruz-Cabeza *et al.* (2015 ▸) analysed a subset of the 2011 version of the CSD, as well as internal statistics from solid-form screens performed at Roche and Eli Lilly, for the occurrence of polymorphism and again found that molecular flexibility and size were not correlated with polymorphism, but that ‘each compound constitutes a new challenge’ when understanding the phenomenon. Through the years, others have also compiled data from internal sources or pharmaceutical databases, such as the European Pharma­copoeia or the Merck Index (Griesser, 2006 ▸; Stahly, 2007 ▸), but such analyses are naturally biased towards pharmaceutical systems which have been screened specifically for polymorphism, and show that pharmaceutical polymorphs occur in approximately 50% of the cases investigated. To determine the relative propensity for *any* organic crystal type to display polymorphism, the present study analyses all organic structures in the 2015 version of the CSD with three-dimensional coordinates known. Making these results available to scientists interested in crystallization for any purpose, beyond just pharmaceuticals, will help to inform all about the relative likelihood of encountering polymorphs of a particular crystal type based on past research efforts.

## 2. Methods   

All CSD searches were conducted using *ConQuest* version 1.18 (section S2 in the supporting information). A text search for ‘polymorph’ was conducted, searching only for organic structures with three-dimensional coordinates known. Previously, van de Streek & Motherwell (2005 ▸) determined that, of all the polymorphic compounds in the CSD, only a few were not flagged with the ‘polymorph’ tag, and they worked with the CSD to correct omissions, indicating that the keyword search should be sufficient to find polymorphic compounds. The van de Streek and Motherwell study looked at all 325 000 entries (organic and organometallic) in the CSD in 2005, whereas in 2015 the CSD contained about 800 000 entries. Our study analysed the 318 524 organic entries present as of 2015. The search described herein yielded 11 909 entries. While this number is substantial, at least two structural forms must be known to characterize a compound as polymorphic, and therefore this number is automatically reduced at least in half. However, many compounds have multiple entries in the CSD, and therefore the number of unique refcodes, or families, may be used to determine the total number of distinct compounds present in the list. In the CSD, a refcode consists of a six-letter code with the possibility of two numbers following. Entries with the same six-letter code should constitute the same chemical entity, whether that is a single component, a salt, a solvate or a cocrystal. Herein, the term cocrystal is defined as a crystal composed of two molecules that are solids at 25°C and 1 atmosphere, in keeping with common usage (Aitipamula *et al.*, 2012 ▸). Examining the polymorph list for the number of refcode families yields 4573 distinct chemical entities which were then further examined. Some cases of conformational polymorphism, where the molecules pack in almost identical unit cells but with minor differences, can be ambiguous to analyse by this method. In such cases, the associated literature was consulted for a comparison of multiple forms.

**Figure 1:**
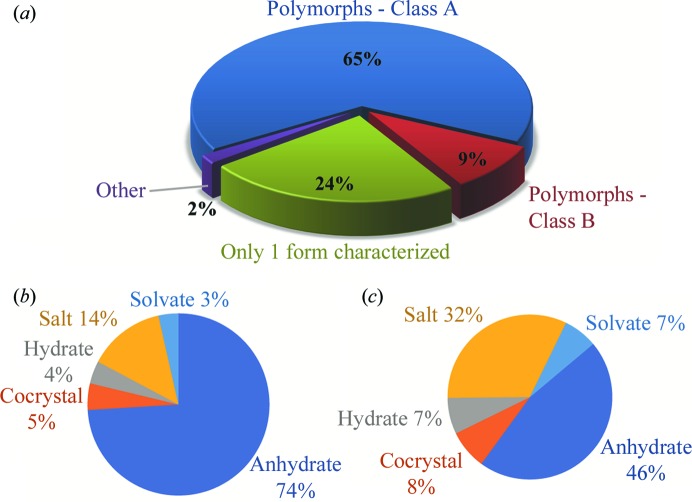
(*a*) A graphical breakdown of the entries flagged as polymorphs in the CSD. Panels (*b*) and (*c*) show further breakdown of the crystal types (anhydrate, non-hydrated solvate, salt, hydrate and cocrystal) for (*b*) polymorphs that can coexist and (*c*) polymorphs with known phase transitions.

An aspect not previously explored by van de Streek & Motherwell (2005 ▸) was the assessment of whether compounds already flagged as polymorphs correctly belonged on this list. Therefore, polymorphism was confirmed for each compound by analysing the unit-cell parameters and simulated powder X-ray diffraction (PXRD) patterns [indicated by van de Streek & Motherwell (2005 ▸) to be the most reliable methods] to confirm the existence of multiple structurally characterized forms of the same chemical entity. Some of these have since been corrected in the 2016 version, and the CSD has also been contacted to bring to their attention those discrepancies not already addressed. No attempts were made here to correct for temperature differences when assessing PXRD patterns, but instead the associated literature was consulted for a mention of polymorphism or phase transitions. For modulated structures where the PXRD would be substantially similar, these would not be flagged as polymorphs in this analysis. Fig. 1 ▸ shows a breakdown of these results.

## 3. Results and discussion   

Most (75%) of the entries in the list of 4573 unique polymorphic refcodes identified above were confirmed as polymorphic compounds (Table S3 in the supporting information). There was, however, a large percentage of compounds with only one crystal form characterized (section S5 in the supporting information). In the light of the assessment by van de Streek & Motherwell (2005 ▸) of polymorphic compounds in the CSD, we were surprised by the number of cases with only one crystal form present and analysed these instances further. Slightly more than 55% of these refcodes do in fact have only one presence in the CSD. In these cases, the compounds were most likely flagged as polymorphic due to their associated publications mentioning this concept when a second form may only have been characterized by a method other than crystallography (Chanh *et al.*, 1973 ▸; Hori, 1999 ▸). The remaining hits were found to have other entries present in the CSD, albeit only by removing the search parameter of having three-dimensional coordinates available. This parameter was chosen in order to select only those compounds with full structural proof of polymorphism, and thus these 347 entries are excluded from the overall listing (section S5 in the supporting information). However, in the remaining few cases where multiple entries were listed with three-dimensional coordinates known, they show up on this list because only one entry was flagged as a polymorph. The reason some of these polymorphic entries are not flagged upon deposition of the structures in the CSD is unknown, but these 61 compounds have been included in the overall list as they do in fact display polymorphism. Three compounds have polymorphic forms of both H and D species, which extends the list to 4576 unique chemical systems (Merz & Kupka, 2015 ▸). The small list of entries in the Other category, which have also been excluded from the overall list, are detailed in section S3 in the supporting information.

When analysing organic crystalline materials, characterization of physical properties, such as solubility or melting, is especially crucial for polymorphs (Rodríguez-Spong *et al.*, 2004 ▸; Byrn *et al.*, 1999 ▸; Hilfiker *et al.*, 2006 ▸). Property measurements should be conducted under comparable conditions, without changes in temperature or pressure, to make concrete conclusions about polymorphic differences. It was observed that a small group (∼10%) of the organic polymorphic compounds were a result of changes in structure due to temperature or pressure (Fig. 1 ▸). Due to the complications with assessing the physical properties of these polymorphs for comparison under the same conditions, we have separated these (termed here as Class B) from the rest of the polymorphs (Class A) to show the occurrence of this type of polymorphism (see section S2 in the supporting information for details of the determination of class B polymorphs). However, both classes are included in the comprehensive list.

The overall list of polymorphic compounds (Table S4 in the supporting information) was broken down further into crystal types (single-component anhydrates, salts, hydrates, non-hydrated solvates and cocrystals), as shown in Fig. 1 ▸. As expected, anhydrates are the most common crystal type of polymorphic compound, with salts a distant second. For Class B, the salt category is much larger, at 32%, than for Class A (14%). In salts, the addition of coulombic attraction/repulsion on top of other noncovalent interactions is a differentiating feature. Perhaps the weaker distance dependence of ionic interactions, overlaid with interactions much more sensitive to intermolecular distance, leads to a far greater prevalence of temperature-dependent phase transitions in salts during changes in lattice constants.

**Figure 2:**
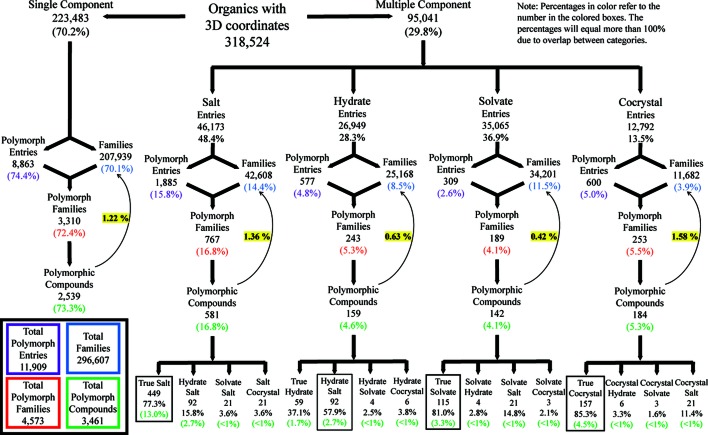
A breakdown of each type of organic crystal found in the 2015 version of the CSD (November 2015, Version 5.37 with one update). Entries are determined from a search for that particular crystal type. Families are calculated based on the number of distinct refcode families within a particular search. Solvates in this case refer to non-hydrated solvates. True crystals refer to compounds with only the minimal chemical units necessary to produce that crystal type (Grothe *et al.*, 2016 ▸). Polymorphic compounds are those on the list of polymorph families which have two structurally determined forms (see section S6 of the supporting information for more details).

To put the listing of polymorphs further into context, the overall occurrence of each type of organic crystal was analysed to compare the number of polymorphic compounds relative to the number of organic compounds in general (Fig. 2 ▸, and section S6 in the supporting information). Comparison of the number of polymorphic compounds with those considered to be monomorphic in the CSD (having only one crystal form characterized) provides a good indication of the relative occurrence of polymorphism in each crystal type. While some crystal types are obvious to search for, such as anhydrates (one chemical unit) or salts (containing ions), most multicomponent systems are more complicated. As per the majority opinion of a group of crystal engineering researchers in 2012, we have chosen in this study to separate hydrates, solvates, salts and cocrystals as separate multicomponent systems (Aitipamula *et al.*, 2012 ▸). Since there is no qualifier in the CSD to search for solid or liquid components, all entries containing two or more chemical units had to be examined individually to separate those that contain at least two neutral solid components (at 25°C and 1 atmosphere) for cocrystals from all solvated entries. Starting with all organic entries in the CSD with three-dimensional coordinates known, the data are then divided into single-component anhydrates and each form of multicomponent system. Each search can be further examined to provide the total number of refcode families (in the same manner discussed above with polymorphism) in order to show the number of unique compounds in each area. Similar searches were also conducted adding the tag ‘polymorph’. Finally, the previous analysis of the number of polymorphic compounds in each crystal type is included. For multicomponent systems, this is also further broken down into subtypes of each group, in order to show the propensity of each subtype with reference to its crystal type. According to the nomenclature of Grothe *et al.* (2016 ▸), ‘true’ crystals of a crystal type refer to structures containing, for example, only two ions of a salt, or only one compound with water for a hydrate.

One area of note is that, among multicomponent systems, true crystal forms are most prevalent for polymorphism in all cases except hydrates, where salt hydrates dominate. Ionic systems often display a high propensity towards moisture sorption, most likely leading to the higher occurrence of salt hydrates than true hydrates. This phenomenon of true crystal prevalence was investigated further to determine if the occurrence of crystals with more than two chemical components is low for all organics, beyond just polymorphs, but the data do not support this suggestion. In fact, over 24 000 unique structures of crystal systems with three or more components have been structurally characterized. Due to the recent focus in the literature on cocrystal polymorphism (Lemmerer *et al.*, 2013 ▸; Aitipamula *et al.*, 2010 ▸, 2014 ▸), these data highlight an attractive area for further study in the future to discern if there is a physical basis for the low occurrence of polymorphism in systems with more than two components.

The overall percentages of polymorphism for each crystal type were calculated by dividing the number of polymorphic compounds by the total number of organic compounds for that crystal type (yellow highlighted values in Fig. 2 ▸). These data give a static picture for 2015, compared with other values presented in the past, and show that cocrystals (1.58%), salts (1.36%) and anhydrates (1.22%) all display approximately the same percentage of polymorphs, whereas hydrates and other, non-hydrated, solvates yield polymorphs with lower incidence (0.63 and 0.42%, respectively). These percentages can change, and have changed, over time. For hydrates, the low incidence is surprising given the ubiquity of water, but for solvates, the origin of the low incidence is more readily understood. Solvates are often not sought after, and frequently occur as an incidental result of a crystallization, such that searching for additional polymorphs is not commonly carried out. The above analysis regarding percentages of polymorphism for each crystal type shows that, as of 2015, cocrystals have a higher propensity for polymorphism than single components among structurally characterized compounds, thus resolving a debate that has lingered for some time (Cruz-Cabeza *et al.*, 2015 ▸; Duggirala *et al.*, 2016 ▸). Due to the small difference in these percentages, however, these data should continue to be monitored for several more years, a task which is now made straightforward because only new structures need to be added to this extensive and scrutinized list.

**Figure 3:**
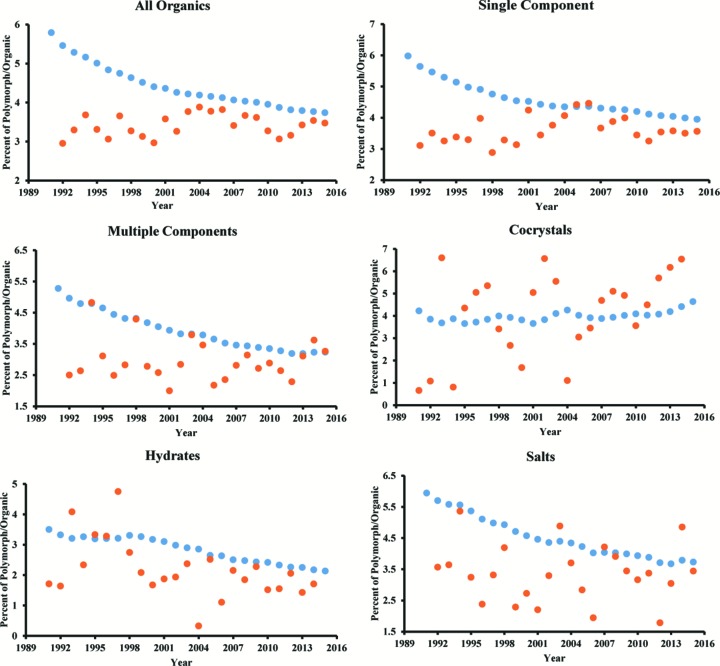
The percentage of polymorphs among all organic compounds in the CSD according to year for specific crystal types. Blue markers refer to the total number of polymorph entries/the total number of organic entries up until that time. Orange markers refer to the number of polymorph entries/the number of organic entries for that year only.

As mentioned above, several researchers have postulated over the years why they believe cocrystals show more or less prevalence for polymorphism than single-component systems (Cruz-Cabeza *et al.*, 2015 ▸; Duggirala *et al.*, 2016 ▸; Aitipamula *et al.*, 2014 ▸). Based on our analysis herein, cocrystals now appear to be the most likely crystal type to show polymorphism. To determine how this concept has changed over time, the evolution of the entries in the CSD was analysed. One of the first publications to undertake an analysis of polymorphism in the CSD (Sarma & Desiraju, 1999 ▸) also addressed this temporal question. In that article, the percentage of polymorphs compared with organics was calculated for every year from 1936–1996, albeit only for single-component systems and with a slightly different set of parameters than those outlined in this study. This analysis has been extended here by looking at all polymorphs for the years 1991–2015 and dividing the number of polymorphic entries each year by the organic entries in that year (Fig. 3 ▸). While this does not take into account the number of unique compounds added each year like the earlier data, it does allow for better analysis of literature trends by including any structural determinations deposited in the CSD for that year that fit the outlined parameters (section S7 in the supporting information). The results show that, throughout these years, the percentage of polymorphic entries in the CSD is constantly decreasing, most likely due to the large and increasing number of new crystal structures being deposited each year, which provides a large background effect. Sarma & Desiraju (1999 ▸) suggested that, by 1996, this decrease in the percentage of polymorphs had already levelled off, although it appears from our extended data to be still changing. The same decreasing trend is seen when splitting the data into single- and multicomponent crystals. However, when looking at specific types of multicomponent crystals, the results show some variance. For hydrates, the percentage of polymorphs is consistently lower than for all other crystal types, which matches the above analysis of polymorph occurrence based on crystal types. For cocrystals, however, the percentage of polymorphs has been consistent in the last 20 years, with ∼4% of the entries being polymorphic. This is reflective of an increase in research activity with regard to cocrystal polymorphism, which is likely a result of the rapidly growing field of cocrystallization in general. These data stand out from all other crystal types, and further exemplify why a breakdown of polymorphic trends by crystal type is a necessary factor to better understand the origins of trends in the phenomenon as a whole.

## 4. Conclusions   

Crystal polymorphism continues to be a very active area of solid-state chemistry research and sufficient structural data have been amassed in recent decades to discern general trends in the field. The fastest percentage growth in entries is in the area of cocrystal polymorphs, whereas the related phenomenon of polymorphism in solvates/hydrates remains relatively less frequent. These results paint a picture of polymorphism as a pervasive phenomenon, albeit one that influences different chemical classes at nonuniform rates. The future challenge is to take the results of this study and discern a physical basis for the differences in likelihood of isolating and structurally characterizing polymorphs of a specific crystal type. Efforts in this direction are ongoing.

## Supplementary Material

Details of CSD searches using ConQuest, tables of refcodes, and raw data from polymorphs/year searches. DOI: 10.1107/S2052252518000660/ed5013sup1.pdf
