# Supplementary material for: Survey and analysis of crystal polymorphism in organic structures
Source: IUCrJ. 2018 Jan 25;5(Pt 2):124–9. doi: 10.1107/S2052252518000660 (PMC5947716; doi:10.1107/S2052252518000660)
Supplement: Supplementary file 1 [file m-05-00124-sup1.pdf]

IUCrJ

Volume 5 (2018)

Supporting information for article:

Survey and Analysis of Crystal Polymorphism in Organic Structures

Kortney Kersten, Ramanpreet Kaur and Adam Matzger

Table of Contents

S1. Polymorphism in Crystal Growth & Design

S2. Methods for Polymorphism Search of the CSD using Conquest

S3. Details of Other Category

S4. Tables of Refcodes for Polymorphism Search

S5. Tables of Refcodes Not Included as Polymorphs

S6. Details of Polymorphism Tree Searching

S7. Raw data from Percentage of Polymorphism Searches

S8. References

IMPORTANT: this document contains embedded data - to preserve data integrity, please ensure where possible that the IUCr Word tools (available from http://journals.iucr.org/services/docxtemplate/) are installed when editing this document.

S1. Polymorphism in Crystal Growth & Design

Crystal Growth & Design (2017) has been published since 2001. For polymorphism articles, a search was conducted for that term for the publication range of each year, and with the restriction to use print publication date (instead of web publication date). Only research articles and rapid communications were considered for this data (reviews, editorials, and perspectives were not included). The total number of articles each year was determined by counting the number of research articles and rapid communications published in each issue, in each year.

Table S1 Percentage of articles published in Crystal Growth & Design that mention the term "polymorph" each year.

Year	Number of Articles Mentioning "Polymorph"	Number of Total Articles	Percent (%)
2001	11	67	16.42
2002	18	101	17.82
2003	33	135	24.44
2004	55	194	28.35
2005	54	320	16.88
2006	71	457	15.54
2007	65	443	14.67
2008	107	733	14.60
2009	103	782	13.17
2010	106	737	14.38
2011	125	723	17.29
2012	154	767	20.08
2013	145	660	21.97
2014	175	770	22.73
2015	134	706	18.98
Total	1356	7595	18.49

Note: Very few instances of the term genetic polymorphism or other instances where the term was not referring to polymorphic crystal structures occur, and do not contribute substantially to the numbers in this journal.

S2. Methods for Polymorphism Search of the CSD using Conquest

Searching for polymorphs in the CSD (Groom, et. al., 2016) was conducted using the ConQuest program (version 1.18, November 2015) with the restrictions of 3D coordinates known and organic only, and a keyword search of polymorph. NOTE: The list of polymorphic refcodes from searching version 1.18 of Conquest provides structures deposited up until November of 2015 (11,909 entries). When searching in version 1.19 of Conquest for structures entered before the Nov 2016 update, the list shows structures deposited up until August of 2015, but also with 192 entries updated after then (11,907 entries). Therefore, it is necessary to note which version is used when searching for this data.

Each refcode family was analyzed by comparing the unit cell parameters and the simulated powder patterns exported to Mercury to confirm the existence of multiple polymorphic forms. The associated publications for each deposited structure were also consulted to determine the situations in which phase transitions were present due to temperature or pressure (Class B polymorphs).

There are 4,573 unique refcode families in the list of 11,909 polymorphic entries. However, three of these compounds show polymorphic forms of both deuterated and protonated versions of the compound, which means there are 4,576 unique chemical entities.

S3. Details of Other Category

Entries in Table S2 are in the Other category from the list of 11,909 entries flagged as polymorphs in the 2015 CSD. There are several reasons why refcodes have been included on this list. For some, two forms were observed due to replacement of hydrogen with deuterium. If the ability to hydrogen bond is removed or altered in any way, it could affect the crystal packing, and this would not fall under the category of polymorphs due to a chemical difference in the structures (Merz, K. and Kupka, A., 2015). Some molecules were listed as a cocrystal in one structure and a salt in another, indicating those two forms would not be polymorphs but instead different compounds. Some structures were disputed between authors as to the classification of the forms as polymorphs. Most of these refcodes needed to be doubled checked in literature to confirm polymorphism, but were not available due to deposition in the CSD as a private communication, giving no experimental data to confirm multiple forms.

Table S2 Refcodes of polymorph flagged entries excluded from total polymorph list.

ADEDIX	DATREV	HIMCHC	MAPZOS	OTOLIC	RESORA	XEPKUZ
AZOXPH	DIWWEL	HIZCOK	MEPHCB	PANISD	REZRIY	XIZDEP
BIPHEN	DODDIJ	ISAZOS	MIYKOU	PFBZAC	SEVNOW	XMPRYO
BOMKOD	DOKDAI	IXEYIT	NEHKIV	PIXPOD	SOPLAL	XOTTAD
BOVYIW	DOVGAW	JABXOZ	NEYLEI	PODSEH	SSALAD	YAFQEC
BPHBAC	EDOTIA	JADNOS	NIPLED	POPGUW	TEKQAB	YIMTUJ
BUNREH	FACZUF	JINVOR	NORDII	QABVEU	UFOCAU	YIVHUI
CBZTCQ	FEQFEN	LACDAU	NOVSIA	QILHUO	VAZRIY	YULGIW
CELHIL	FUFJAT	LESQAC	ODAKUA	QINCUL	VETVOG	YUMKOH
COKVON	GADGUN	LUTROJ	OFOWOW	QUKVUN	XEBMIB	YUYPUD
COVJON	HAXFER	MAMBOR	OJEJAO	RALMOG	XECDUF	ZEXLUJ

S4. Tables of Refcodes for Polymorphism Search

Table S3 contains all refcode families from the search for "polymorph". Green cells indicate compounds which have been deemed Class A polymorphs. Pink cells indicate compounds which have been deemed Class B polymorphs. Yellow cells indicate compounds which had multiple entries listed, but did not have multiple polymorphic forms. Blue cells indicate compounds which only had one entry in the list. Orange cells indicate special cases which constitute the "other" category shown in Table S2. Those entries which have a "-D" listed after the refcode indicate compounds which had polymorphic forms of a deuterated compound. In three cases, there were polymorphs for both the H and D forms of the compound, and these are listed separately. The percentages of each group of compounds are shown in Figure 1 in the main text.

Table S3 List of unique refcodes (4,576) resulting from the search for polymorphs in the 2015 CSD

REFCODE	DABHAP	GAFXAN	KETJUP	NUNHUZ	RURROM	VIXPOJ
ABADIS	DACMOR	GAGSIR	KETTUY	NUNSIY	RUVPII	VIZHIW
ABAKEV	DACTDO	GAGVAL	KETVEK	NUPBAD	RUVZOY	VIZYUA
ABAZOS	DADDER	GAHLEG	KETYAJ	NUPBEH	RUWYIR	VOBHAX
ABEFUJ	DADTEH	GAJDUQ	KEVMEF	NUPCOS	RUXWAI	VOBNAB
ABEKUN	DAFBIV	GAJFAY	KEWQAG	NUQXAA	RUYZOA	VODGUR
ABIHIC	DAGWUF	GAKGII	KEWYES	NUQXEC	RUZWUE	VODLEG
ABIKEB	DAGYIV	GAKGIJ	KEXYOC	NURAMH	RUZXEP	VOGCOL
ABOPUC	DAHMII	GAKNAH	KGLUCP	NURFAH	RVOXMI	VOHKEK
ABUDAD	DAHPIL	GAKWIA	KHACDC	NURJEP	SABCII	VOHNOV
ABUNIU	DAJKIK	GAKWUK	KHTRON	NUVMAS	SACBAA	VOLJIR
ACADIR	DAJVUG	GALCAX	KIBBUS	NUVXAE	SACRET	VOLZEC
ACAFEQ	DAJZAQ	GAMBUT	KIBPEQ	NUWTOP	SACXID	VOMWUR
ACAMUN	DAKFUQ	GAMJEJ	KIBQOC	NUXFAP	SACXOJ	VONYOO
ACARBM	DAKPUA	GAMJUZ	KICTUN	NUXJUM	SADJAI	VOPJUH
ACBNZA	DAKXUI	GANYEA	KIDJUD	NUYCEQ	SADVUQ	VOQHIU
ACDECT	DALJAB	GANYOK	KIDQIZ	NUYDER	SAFJIT	VOQHOA
ACEDAN	DAMPEO	GAQHIR	KIFHUC	NUYJEW	SAFPIY	VOQNIY
ACEMID	DANGAL	GASFAH	KIFLEQ	NXENAM	SAGWUU	VOQXOP
ACENYL	DANQEP	GAXLEW	KIFMOC	OBAZOI	SAGXOP	VOSQUP
ACESAE	DANTEN	GAXXUY	KIFPUL	OBEHAE	SAGXUT	VOTTON
ACETAC	DAQDII	GAXYEJ	KIGKER	OBEQAN	SAGYIK	VOXDUH
ACETEJ	DARGOT	GAXYIN	KIGTOL	OBEQER	SAHZOS	VOXFUJ

ACICAR	DARWAU	GAYTIJ	KIHYOQ	OBEQIV	SAJGAL	VUBQAM
ACIGID	DASMEP	GAZFAO	KIJGUF	OBIDOS	SAJMOH	VUBYAU
ACIKAZ	DASPAO	GAZFES	KIJQEA	OBOZIQ	SAJVEE	VUCFUU
ACOMUC	DATDIL	GEBSOV	KIJSIH	OBUPUY	SAKMEW	VUFGEI
ACONEN	DATNBZ	GEBSOX	KIMKUM	OBUZUI	SAKPOJ	VUFKAJ
ACONIR	DATREV	GEBTUC	KIMLEX	OCAKEJ	SALCAN	VUFKEM
ACOVAQ	DATWUS	GEDKEH	KINQIJ	OCALAG	SALHOC	VUHFIO
ACOVIY	DATZAA	GEFGII	KINSIL	OCHTET	SALIAZ	VUJMAO
ACOVOE	DAVLAP	GEFREO	KIPTOU	OCIMUJ	SALMID	VUJNAP
ACOYOG	DAVQAS	GEGGEF	KIRHEA	OCIYEF	SALOXM	VUJSOJ
ACPRET	DAVVUR	GEGLOT	KISQEJ	OCMETD	SAMCAL	VUJTOJ
ACPYRI	DAVWAY	GEGNUB	KISQUZ	OCRSOL	SAMPYM	VUJXIH
ACRDIN	DAWBEI	GEGXAS	KIVSEP	OCTATT	SANACM	VUJZAB
ACRLAC	DAWFUE	GEHBAX	KIXLOS	OCTCOS	SANCEP	VUKGAJ
ACSALA	DAWGAL	GEHXEX	KIYSIU	OCUJOM	SAPJAV	VULCIQ
ACTART	DAWHOY	GEJCAZ	KIZCEC	OCUSUA	SAPVAI	VUNYEI
ACTDGU	DAWMAP	GEJCED	KIZMAI	ODAJIM	SARCAC	VUNYUY
ACTOLD	DAWSOJ	GEJPOC	KNATAR	ODAKUA	SASFAU	VUPTAC
ACULIV	DAWVOM	GEJPUI	KNTMET	ODASAO	SASKII	VURNAX
ACUMIU	DAXLIZ	GEJXAU	KOFKAR	ODEFOT	SATART	VURWAH
ADAJUK	DAXMAS	GEKLOX	KOFKEV	ODEGIN	SATCOG	VURYOW
ADAMAN	DAXSOM	GEKMOA	KOFKIZ	ODIGOZ	SATPIO	VUSDIX
ADEDIX	DAXTIH	GEKREV	KOJNAA	ODOBIT	SATRAG	VUSFIY
ADIPAC	DAXYUX	GEKZIF	KOJSUZ	OEPYMI	SATSUD	VUSZEO
ADIPAM	DAYWAC	GELCIK	KOKJOL	OETPOA	SAWKIL	VUTGOG
ADMNTB	DAZABZ	GELDEI	KOKQUW	OFEJUG	SAWKUX	VUTZIT
ADPOSM	DAZBAJ	GEMGAG	KOMDAR	OFEXAY	SAXDIE	VUXRIQ
ADULEQ	DAZCLA	GEMMAN	KOMZUJ	OFIQUR	SAXJEG	VUXZEU
ADURIA	DBEZLM	GENLAN	KONTIQ	OFNAPH	SAXPOW	VUZPAH
AFAZEM	DBMBPO	GENQIB	KONZEU	OFOGAR	SAYMUB	VUZWAO
AFECUJ	DBNTHR	GEPPAU	KOPDAW	OFOWOW	SAYNAI	WABZEE
AFIKEH	DBPHEN	GEPXOO	KOPJUW	OFOXEN	SAYVEV	WACHEP
AFIPIQ	DBPOLO	GERPOJ	KOTDEC	OFUFIG	SAZHOQ	WAHSAZ
AFLATM	DBXZCP	GERWEF	KOWYEA	OFUVUG	SAZLEL	WAHSEC
AFORIY	DBZSEL	GERWIJ	KOYMES	OGABEF	SAZXAS	WAJRIK
AFULIY	DCAETO	GERZAG	KPALMA	OGAPET	SBBCHD	WAJSIL
AFUWED	DCBFRO	GESBIR	KTCYQM	OGELUI	SEBFAG	WAJTEI
AFUYIJ	DCDHNQ	GESNUN	KTMSIO	OGEPUM	SEBFAH	WAJXEL

AGAKUP	DCHEXC	GETFUG	KTTOXC	OGERAU	SEBTAU	WAKCIW
AGUAHP	DCHXCS	GEXXIQ	KUBNOK-D	OGIDEN	SECZAB	WAKLUQ
AHADAQ	DCHXYK	GEYRIL	KUBWAF	OGILIA	SEDTUQ	WAMDOD
AHAPEG	DCLANT	GEZREK	KUCSAE	OGISON	SEGJAP	WAMFAS
AHBONM	DCLBEN	GEZSAH	KUCYOY	OGUROZ	SEGKEW	WAMMAY
AHBPRG	DCLBQN	GICTIV	KUDFUM	OHADAD	SEGQUS	WAMXOX
AHEGEZ	DCLNAP	GICVUJ	KUDHIC	OHIBOW	SEGXAE	WANLAY
AHEJAZ	DCLPHM	GICYUN	KUDLIG	OHIJAR	SEHNAW	WANLON
AHEMAB	DEBMOM	GIDHEG	KUDXAK	OHOVAJ	SEINDI	WANMON
AHICIF	DEBMUS	GIDLUB	KUFLUU	OIPFRP	SEJWUZ	WANMUU
AHOFAE	DEBNAZ	GIFPAM	KUGGIC	OJEJAO	SEKXUC	WANNUV
AHONUG	DECFDP	GIFSOD	KUGRIO	OLATUR	SEMBOB	WANSAG
AHOXLH	DECYIU	GIFSOE	KUGZAP	OLEBAJ	SEMYIT	WAPBUK
AIPBAR	DEDBUJ	GIFSIX	KUHPIM	OLECAC	SENGEX	WAPCAR
AJAJEA	DEFDUN	GIGXAX	KUHPIR	OLENAN	SENGOH	WAPREL
AJAPOR	DEFQIP	GIGYEA	KULSOA	OLEYEK	SEOTCR	WAQTUF
AJETAL	DEFRUB	GIHWOJ	KUMCAX	OLUPAM	SEPBAS	WARRIS
AJEYAQ	DEGGEB	GIJTEA	KUMCEB	OMAYUX	SEPCAT	WASRAJ
AJUVAC	DEHREN	GIJTIC	KUNVEU	OMCSIO	SEPDIA	WASTEP
AJUVEG	DEHSIS	GIJTUO	KUPMUE	OMEFAN	SEPTIS	WATREP
AKEMIN	DEKDAY	GIJVIG	KUPPOA	OMEKAS	SEQBAR	WAVCUR
AKOVOL	DEKDIG	GIKBOT	KUQLEO	OMIYEO	SEQHUS	WAVYAT
ALABUK	DEKFUU	GIKKAO	KUQRUK	OMOPAH	SESHUT	WAVZAV
ALADIB	DEKYEX	GIMBUZ	KURBOP	OMUZOM	SESPEL	WAWDUU
ALAHEA	DEMQIW	GIMNIA	KUSVAW	ONAVEF	SESYAR	WAYCUW
ALDCAR	DEMYEZ	GIMPOI	KUSVEZ	ONITAN	SETDTC	WAZTEY
ALIBUS	DENWUP	GINCAI	KUSVID	ONOXUL	SETLOU	WEBCOV
ALOVUS	DEPOAM	GINKIZ	KUSWUR	OPIJAZ	SETSAN	WEBMAR
ALOXAN	DERBEH	GIPGOB	KUTLIW	OPITOX	SEUREA	WEBNUO
ALUQEE	DERRAT	GIPGUH	KUTMAP	OPOXAZ	SEVGUV	WEBSIF
AMBACO	DESKER	GIRBUF	KUTQIB	OPSIOX	SEVNOW	WECCUC
AMBNAC	DESYIJ	GIRNOL	KUVWON	OQUPUM	SEVPEP	WECGIW
AMBNZA	DETBAA	GISNOM	KUVZAE	ORABEP	SEYDIJ	WEDLEW
AMBZPH	DETBIO	GISNUS	KUWHOA	OSAGAR	SEYHOT	WEDSEE
AMEPOS	DETBUZ	GISRIJ	KUWPUP	OSAGEV	SEYLEN	WEFJUN
AMHCLA	DETSUQ	GISYOX	KUXJUI	OTIYUM	SEYXUR	WEFKEY
AMIRIY	DETZUX	GISZIR	LABFID	OTIZAT	SEYYIG	WEFKIC
AMNTPY	DEVSAZ	GITWUC	LABHAX	OTOLIC	SEYZON	WEFVIN

AMPETS	DEWVOQ	GITXAK	LABJON	OTUCOW	SEZXIE	WEHXUD
AMTCAR	DEXBUF	GIVJUQ	LABSEM-D	OTUDAJ	SIBTAZ	WEJNEE
AMXBPM	DEXVEH	GIXHUR	LABSEM-H	OTUDIR	SICXIL	WEKHEA
AMYTAL	DEYNAP	GIXPEI	LACDAU	OVAYAM	SIDHOD	WELHEA
ANISAC	DEZJIB	GIYDEZ	LACTAK	OVAYIU	SIDJEU	WELZUK
ANISUC	DEZRAD	GIZFIF	LADGAY	OWEMIN	SIDSUT	WEMGAW
ANLINB	DEZZAL	GIZYIY	LAFKAE	OXACDH-D	SIFLOI	WEMGIE
ANONEX	DGLYAC	GLUCIT	LAGWOH	OXADOH	SIGFOE	WENVER
ANTCEN	DGLYCN	GLUCSA	LAJZED	OXALAC	SIHBIV	WEPDEB
ANTCYB	DHANQU	GLURAC	LAKWOL	OXAZOL	SIHNII	WERNOW
ANTMEU	DHNAPH	GLUTAS	LALWIF	OXBICB	SIHVOU	WERROA
ANUCES	DHXANT	GLYCIN	LAMYAA	OXIBZN	SIKLIH	WERTUI
ANUKAW	DHYANT	GLYGLY	LANXOO	OXTCNT	SILTOW	WERZOI
ANUMEC	DIBDEY	GNAPHB	LARGIN	OXTPTZ	SILTUC	WERZUO
ANUMIG	DIBTEP	GOBVEA	LASBUC	OXYTET	SILVAL	WESBAX
APAPAD	DIBZAQ	GOBYAX	LATNEB	OYEWUL	SILXUG	WESBIF
APATOB	DICCEX	GOCJIT	LATSEG	PABHAB	SIMXUI	WETFAD
APENTN	DICGED	GOCPAP	LATTAD	PABNAJ	SINDIG	WEWLUG
APESOE	DICNIM	GOFVAZ	LATTOR	PABNIR	SINKEF	WEWWAW
APGPAL	DICNUY	GOGQOJ	LAURAC	PABZAM	SINZIY	WEXLIU
APLYSU	DIDMTE	GOKCOA	LAVMOK	PACBOK	SIQGOO	WEXWOL
APUDEV	DIFVET	GOKQAY	LAVYOX	PACFOO	SIRDUT	WICSUW
APUSAF	DIJYOL	GOLLUO	LAWYOY	PACGAB	SIRJUZ	WIFCAQ
APUTUB	DIKHAH	GOMFOD	LAXJOK	PAHDEI	SIRMIQ	WIFZUI
APUYOA	DIKTAT	GOPDUK	LAYMED	PAHRIZ	SIRXUM	WIHBAS
APYRDN	DIKVID	GOQCEW	LAZHID	PAJCOT	SISSOE	WIJNOS
AQOMAU	DILFEL	GOQPOR	LAZPAD	PAJREY	SITDII	WIKDEZ
AQOSIJ	DILKEO	GOQZUH	LAZRAF	PAJZAC	SITKEL	WIKVIV
AQOZUB	DILVIF	GORBOE	LCYSTI	PAKPUO	SITLUD	WIMBAV
ARSMET	DIMETH	GORKUV	LCYSTN	PAKQUP	SIVBEE	WIMFON
ARUWUG	DINPAR	GORVIU	LDOPAS	PAMTAY	SIVCAB	WIMSOA
ASATET	DINRUP	GORXAM	LEBCIE	PAMVIK	SIWDEH	WIMTER
ASATOD	DINSOK	GORXEQ	LEBGEE	PANISD	SIWDIL	WIMWOE
ASEHUB	DIPGIS	GORXOA	LEBKIN	PANQUO	SIWZOP	WIMZOH
ASEQEU	DIPHUH	GOVQAJ	LECGUV	PANQUS	SIXCIM	WIMZUN
ASETOI	DIPLAR	GOVQEN	LECROB	PAQVUA	SIXFIP	WINBAW

ASITEC	DIPQAU	GOVQOX	LEDQEQ	PARQUI	SIXKAN	WINBEA
ASIXEF	DISQIG	GOVRAK	LEHWAX	PARSON	SIYDOV	WINBIE
ATABAZ	DITBEN	GOVREO	LEJDUZ	PARTEN	SIYNET	WINBIG
ATAGAD	DIUREA	GOXJOS	LEJFAH	PASPLB	SLFNMA	WINDEC
ATAWIC	DIVHOF	GOXWAR	LEJKIU	PASWUD	SLFNMB	WINJAF
ATCPEN	DIWBAM	GUBJOE	LEJMOE	PASXAK	SLFNMD	WINVAR
ATDZSA	DIWGAS	GUCNUP	LEJNET	PASXIS	SLFNMF	WINWUL
ATEBIK	DIWKOK	GUCWOQ	LEJNIX	PATSEJ	SOBJAV	WIPKUB
ATIDOX	DIWMOM	GUCYAE	LEJXON	PATSIM	SOBNEE	WIQLEN
ATIWOP	DIWVEM	GUFMOJ	LENBOV	PATVEM	SOBPEE	WIRXAW
ATPRCL	DIWWEL	GUHDIW	LEPMEZ	PAVBEU	SOBRAE	WIRYEB
AVIHIX	DIXBIW	GUHDIY	LEPPIF	PAXWEQ	SOBSUX	WISBEF
AVIWEH	DIXNIH	GUHHAS	LEPPUS	PAZCBN	SOCRUZ	WISGEK
AVIYEK	DIXTEN	GUJNAA	LEPRON	PAZOXN	SOCSIO	WISRAR
AVOJUR	DIYJUQ	GUKPEH	LEPTER	PAZPTE	SOCTOT	WITDEK
AVUFED	DIYLIG	GUMXAO	LEQKEY	PBBTAZ	SODCIX	WIYVEF
AWAJOX	DLABUT	GUPICA	LEQPIH	PBOXAT	SODDUL	WIZFEQ
AWAKIS	DLASPA	GUQPOZ	LEQRIK	PBRPOL	SODKAZ	WIZHUJ
AWAXOM	DLMALC	GUQRIU	LESFOF	PBTCHT	SOFPAG	WOBHUR
AWAYAZ	DLMAND	GUQZUP	LESHOI	PBUPEA	SOGNUX	WOBMUV
AWAYON	DLMETA	GURBUQ	LESQAC	PCBZAM	SOGUAN	WOBQEK
AWELOE	DLMSUC	GUTYAX	LETGIA	PCCOCT	SOHXAO	WOBQIO
AWEWAB	DLNLUA	GUVBIK	LEUCIN	PCHSAN	SOHXIY	WOBWIT
AWIFUI	DLSERN	GUXZUW	LEVCUK	PCLPYR	SOHXUI	WOCJED
AWIHOE	DMADEN	GUYBAF	LEVNAC	PCPZOL	SOLBEC	WOFHIK
AWIHUK	DMAFBZ	GUYGOX	LEVNUW	PDABZA	SOLPUF	WOGRIT
AWUNOV	DMANTL	HABFAS	LEVWEP	PDHTEZ	SOLSIW	WOGVEV
AWUWIY	DMBMCL	HABJUP	LEXVEP	PDSILZ	SOMKIQ	WOJXUO
AXAJOZ	DMBOPN	HACTPH	LEYXEU	PDTOMS	SOMKOU	WOKLUF
AXOGAW	DMBZAC	HADKIG	LEZJAB	PEAMAN	SOMTUJ	WOKMUG
AXUBUR	DMEACL	HADKUT	LEZPUC	PECNOC	SONSUK	WOKPER
AXUCEC	DMETSO	HADNOP	LEZZAS	PEDTAV	SOPLAL	WOLXOK
AXUWAS	DMEWAR	HAFVIV	LGLUAC	PEDTUP	SOPRUK	WOLYUR
AYIBOZ	DMFUSC	HAJSAO	LHISTD	PEDWOM	SOSMAO	WOLZAY
AYOVUG	DMHXDM	HAJYUN	LHLASP	PEFBAD	SOVFOA	WONVEA
AZADAG	DMMTCN	HAKTAP	LIACET	PEFGEO	SOVWIJ	WOQBAF
AZAFAJ	DMNPYO	HAKVAR	LIBRUL	PEFGIR	SOVXUY	WORMAN
AZAJAN	DMOXBA	HAKVUL	LICDIM	PEFGOX	SOVYOT	WORWAD

AZBNBT	DMPOXP	HALMEO	LICMER	PEFGUC	SOVZAG	WOSJIZ
AZBZCX	DMTRYP	HALMUC	LICMIT	PEFHOZ	SOXHUI	WOTJOE
AZELAC	DMURAC	HAMNEO	LICPOE	PEFJER	SOXLAS	WOTZAG
AZERIH	DNTNAP	HANFOS	LICRAS	PEFKAO	SOXVEG	WOVGIZ
AZERON	DOBCDC	HAVBIQ	LICSOF	PEFKOC	SOXVIK	WOVMID
AZESEE	DOBRUH	HAWRAZ	LIFFEM	PEFKUI	SOYSOP	WOVYOV
AZETAB	DOBTOD	HAXBUD	LIFHOZ	PEFLET	SPSNAP	WUCGUW
AZIBYR	DOBTUJ	HAXFER	LIFORM	PEFMET	SRFORA	WUKGAK
AZOMAD	DOCCEF	HAXHET	LIGPUM	PEFMEU	SSALAD	WUKKOC
AZOXHD	DODBIJ	HAXKIC	LIGVUU	PEFNIZ	SSOXAM	WULZIM
AZOXPH	DODDAB	HAXLAV	LIGXUU	PEFTIE	STARAC	WUMKUK
AZSTBA	DODDIJ	HAXMAW	LIHGAM	PEFZIL	STENON	WUNYAH
AZSTBD	DODRIZ	HAXVAF	LIHXUW	PEGVAY	STILTQ	WUQGAQ
AZUGIM	DOFNAN	HAYKUO	LIJYAE	PEGWII	STPYAZ	WURMOM
BAAANL	DOFSUM	HAZBEQ	LIKBOW	PEHZUX	SUBTIS	WUTCEU
BACLCH	DOGWOL	HAZFAP	LIKCEN	PEJBUC	SUCACB	WUTSIO
BACRIH	DOHBOC	HAZGUK	LIKGAN	PEJCAJ	SUCHOL	WUVKEE
BADPEB	DOHQAS	HBTMDX	LIKGUI	PEJHOD	SUCNOT	WUVYUH
BADTEX	DOHQEW	HCCYHD	LILHAP	PEJJOF	SUCROS	WUWTIR
BAFLID	DOHREX	HCLHEP	LILJUL	PEJKAS	SUDXAS	WUWTOX
BAGFIY	DOHSEA	HCSBTZ	LILRIJ	PEJKIA	SUHVEW	WUYPOW
BAHFAR	DOJFAJ	HDBANT	LILSUU	PEKGER	SUKNIW	WUZHOP
BAHNUU	DOJHAN	HDXMOR	LILWUY	PEKJEW	SULAMD	XABREY
BAHOXL	DOKDAI	HEBFUR	LILXIN	PEKJUM	SUPBEK	XADXOR
BAJYIU	DOKGUG	HEBQOV	LILYAG	PEMZAI	SUPFEO	XAFMIC
BAKQEL	DOKNOI	HEBWIV	LILZOV	PEMZAJ	SUQFIT	XAJPII
BALNIN	DOLBIR	HECXAO	LIMBIS	PENCEN	SUQWIK	XAQXOE
BALWEQ	DOLJET	HEGHUX	LIMCAL	PENJAT	SURGOC	XAQYEV
BANGOM	DOLNEZ	HEGPAK	LIMCEP	PENJAV	SUSJEV	XARHII
BANHOO	DOMBOW	HEHDOP	LIMNEA	PENMPH	SUSKAS	XAVPAM
BANJEH	DOMZIQ	HEJTAR	LIMNIE	PEPGUM	SUSKUM	XAVTOF
BANQOX	DONTIJ	HELBAD	LINMIF	PEPHUN	SUTHAZ	XAVVIB
BANYIY	DOPDAN	HELHEN	LIPBIW	PEPQEG	SUTWIO	XAXCOQ
BAPBOJ	DOPLOL	HELHIR	LIQQIN	PERLEN	SUWLAX	XAYBOO
BAPLOT	DOPPAB	HEMLAM	LIRRAG	PERPIL	SUWMAY	XAYGIP
BAQBEA	DOPPOP	HEPJAN	LISDOH	PERYTN	SUWMIG	XAYGUB
BAQHOS	DOQNAY	HEPJOC	LITLEG	PERYTO	SUZGIE	XAZCEH
BAQJEK	DOQWEN	HEQFIT	LIVNOV	PETLAD	SUZYES	XAZHOW

BARBAC	DOSQAD	HERMOH	LIVSAL	PETNEI	TABCAC	XAZVOK
BARBAD	DOVGAW	HESDIT	LIVVIW	PETNER	TACRIB	XAZWIF
BARWUM	DOVGEC	HETDAM	LIWRAK	PETXUH	TADLIU	XAZYED
BASVUM	DOVJOP	HETPAL	LIYTIW	PEVQUD	TADTAU	XEBKEV
BATCAA	DOVJUT	HEVMEA	LOCVOO	PEWXAQ	TADZAZ	XEBMIB
BATWOI	DOVVIT	HEVWAI	LOFDOB	PEXKOS	TAFBAD	XEBQOL
BATWUP	DOWVOC	HEVXUB	LOFEXD	PEXTAO	TAFBUZ	XECDUF
BATZAZ	DOWWET	HEWLOL	LOFKAS	PEXVOE	TAGZOQ	XEDLIC
BAWNIW	DOWWIX	HEXBME	LOFKIB	PEYMIQ	TAJCUD	XEDVIM
BAWSAT	DOWWOD	HEXCET	LOHKOK	PEZBOL	TAJQUR	XEDVOS
BAZJAO	DOWWUJ	HEXDEC	LOHKUQ	PEZLOV	TAJVAC	XEFJOI
BAZLUJ	DOWXAQ	HEXWIQ	LOHTAE	PFBZAC	TAJVIK	XEGMAX
BAZYAC	DOWXEU	HEYHUO	LOHVEK	PFLCPC	TALJIZ	XEHHEX
BBEZAM	DOWXIY	HEYJOK	LOKNII	PGLUAC	TAMBUE	XEHQAC
BBZFRO	DOWXOE	HEYQOR	LOLYUG	PGTAPA	TAMZIQ	XEHQEG
BCHBZP	DOWXUK	HEZZIV	LONKEE	PHARFE	TANTOX	XEJLII
BCOCAN	DOWYAR	HIBCOK	LONKII	PHBARB	TAPMEC	XEJNUV
BDOTHY	DOWYEV	HIBGUV	LOPQEO	PHCBUT	TAPZIT	XELLEF
BDTOLE	DOWYIZ	HIBVAP	LOQDIG	PHENAN	TAQCET	XELLOP
BEAVBA	DOWYOF	HIBXOF	LOQLAE	PHENAZ	TAQDIZ	XENDUP
BEBCIU	DOWYUL	HICDUS	LOQNAI	PHENOL	TARJIG	XENXUL
BEBMAX	DOWZAS	HICJAG	LOQQEN	PHISCY	TARNIL	XEPKUZ
BECDAO	DOWZEW	HICJIO	LOQSEP	PHNSNB	TARTAM	XEPXOF
BECJEY	DOWZIA	HICWOF	LORVAP	PHOGLY	TARTMM	XEQGUX
BECKUR	DOWZOG	HICWUL	LORWUL	PHOSBZ	TASCUM	XERBAX
BECMED	DOWZUM	HICXIA	LOTLIR	PHPLAC	TASVIS	XESKEM
BECMUT	DOXANN	HICYAT	LOTLUD	PHPYRO	TATNBZ	XESSUJ
BECWOV	DOXDIS	HICZEY	LOTZEZ	PHTGLY	TAWRIT	XETKOX
BEDMIG	DOZJAD	HICZIC	LOWFUA	PHTHCY	TAYTUI	XETZIG
BEDRIL	DOZYAS	HICZUO	LOXHAJ	PHTRPL	TAZPEN	XEWMER
BEFVAJ	DPBCOC	HIDDAZ	LOZDUZ	PIBBEH	TBCHTS	XEWMOB
BEGLAA	DPENAM	HIDFEF	LOZYII	PIBDIP	TBMNBZ	XEWZAB
BEHZOF	DPGUAN	HIDHAD	LPYGLU	PIBGOY	TBTYAC	XEXKEQ
BEKMEK	DPHACT	HIGBII	LSERIN	PICAMD	TBUBEN	XEZFIT
BELBAV	DPHETH	HIGNIV	LSERMH-D	PIDFEN	TBUHLB	XEZGAK
BELBUP	DPHNOL	HIHKUE	LSERMH-H	PIDGOZ	TBZMBA	XEZGEP
BELLIO	DPHOCE	HIHROF	LUBPOP	PIFHOC	TCAPIN	XIFRUZ

BEMBEB	DPHSAM	HIJFIP	LUDYEO	PIFKIA	TCBCAO	XIHFAW
BEMLOU	DPIPDS	HIKNEW	LUFCIY	PIFLAR	TCBENQ	XIHQEK
BEMWEV	DPOCRS	HIMCHC	LUFQIO	PIFZAG	TCHXYP	XIJKIK
BENCLN	DPOCTT	HIMGOA	LUHQIO	PIHLOJ	TCLBEN	XILPUD
BENNIT	DPPETH	HIMWIJ	LUKXAQ	PIHSEF	TCLCYH	XILQOY
BENPRL	DPPSEM	HIRPAZ	LULKEJ	PIHSUV	TCLOBQ	XILREP
BENZEN	DPRTMA	HIRQIK	LULKIN	PIJHIA	TCLTET	XIMBZB
BENZID	DPSIFL	HIRQUW	LULLOT	PIJXEL	TCMPCQ	XIMMEL
BENZIE	DPTOTE	HIRREH	LUMHOR	PILDIX	TCTCPH	XINBEB
BENZIL	DPYRAM	HIRROR	LUMNPO	PIMBAP	TCTMBE	XINRUH
BEOACT	DQUNDS	HIRYIR	LUMXAS	PIMELA	TCYETY	XIPYIF
BEQXAW	DTACAC	HISGAS	LUNPOZ	PIMTAZ	TDBOCP	XISJUE
BERCAD	DTBPTR	HISREH	LUNRUI	PINCOL	TDCHCH	XISLIV
BERPAP	DTCTCQ	HISRIL	LUPGIN	PINPAD	TEBETU	XISLUG
BERPIY	DTHDOX	HISROR	LUPGUY	PIPGIG	TEBGAI	XIYXIN
BERTOH	DTHDSX	HISRUW	LUPVOH	PIPINE	TEBGUC	XIZDEP
BESKAL	DTHTPP	HITSAG	LUQKEO	PIPMDC	TEBHEN	XIZPEB
BETANC	DTTDDO	HIVLAA	LUQLIU	PIPPTC	TEBSSU	XMPRYO
BETJAL	DTTROP	HIVROT	LUQVIC	PIQLIK	TECCAF	XNAPAC
BETPIZ	DUCKOB-D	HIWJIG	LURHAJ	PIRNOV	TECXII	XOCFOK
BETUTE	DUCLUJ	HIXHIF	LUSKUF	PIRQIS	TECZAE	XOCJEE
BEWFOY	DUDCUC	HIZCOK	LUTDUR	PIVGEH	TEDAPC	XOCXUI
BEWKUJ	DUDNAS	HMBENZ-D	LUTPEV	PIVIND	TEDFUF	XODCEZ
BEWNAT	DUDZIL	HMHOCN	LUTROJ	PIWBUT	TEEXAN	XOGDIH
BEWPEY	DUHBAK	HMPPYD	LUVYUX	PIWDUV	TEGNEA	XOGDUT
BEWYUY	DUHSEE	HNIABZ	LUXXAD	PIWKOV	TEHGUI	XOHYIE
BEXTAB	DULZIT	HOBBOP	LUYJEU	PIWPER	TEHKOG	XOJMIT
BEYWAF	DUMVAJ	HOCYSL	LUYMOH	PIXPOD	TEHMEY	XOJXOL
BEYWEJ	DUNGIC	HODWED	LYSDOL	PIYQEU	TEHNAW	XOKMOZ
BEYWIN	DUNGID	HOFFEN	MABZNA	PIYSIA	TEHNID	XOKXEC
BEYZIO	DUNWIT	HOFLOE	MABZOX	PIYSUM	TEHTUX	XOKYAZ
BEZQAZ	DUNWOZ	HOFMAQ	MACCID	PMAANO	TEJKUO	XOKZAA
BIBKUS	DUNXAM	HOFNOF	MACPRP	PMETRA	TEKMED	XOLCUX
BIBYUG	DUPTOX	HOFNOH	MACZIB	PMZPCY	TEKQAB	XOLHUC
BIBZUJ	DUQJOP	HOFWAB	MAFPOA	PNEOSI	TELKUQ	XOLJOZ
BICCIZ	DURDAV	HOHMOG	MAGTUK	POBENZ	TELYAK	XOLQUM
BICQUB	DURGAY	HOHRIF	MAKDEH	POBKOG	TEMBES	XOMTUP

BICVUE	DURZAR	HOJQEE	MALEHY	POBPIG	TEMCIX	XOPWEF
BIDLOP	DURZUM	HOJQII	MALIAC	POCPUS	TENJAX	XORLIZ
BIGTIU	DUVFUV	HOJROP	MALNAC	PODQIJ	TENQUA	XOSBIR
BIHXIA	DUVYEZ	HOKMAW	MALOAM	PODSEH	TEPHTH	XOSGUH
BIJDON	DUVZOJ	HOKPUS	MALONT	POFHUN	TEPNIT	XOTQED
BIKHIN	DUWBUT	HOLJAU	MALSOJ	POFLAX	TEPSEM	XOTTAD
BILCOO	DUXSAR	HOLLIE	MAMBOR	POFRAE	TEQMUX	XOWLIG
BILCUW	DUXSEV	HOLVAG	MAMGUD	POGVAJ	TERMEI	XOYQIN
BILNAN	DUXSUK	HOLVOU	MAMMCL	POHJON	TESTOM	XOYVOX
BILNOZ	DUYCUW	HOLWAH	MAMPUM	POKLUY	TETAMI	XOZBUK
BILQEU	DUYZUT	HOLWAI	MANMUJ	POKSAJ	TETBBZ	XOZJOM
BIMCEG	DXCYTD	HOLWOV	MANPIM	POMDAW	TETBEZ	XUCKOX
BIMYAX	DXYLEN	HONQOQ	MANSTB	POMLIO	TETDAM	XUDWUO
BINMEQ	EABVEI	HOQABS	MAPLIZ	POPFAC	TETGUU	XUDXAV
BIPCOS	EAPTZC	HOQSUB	MAPMIP	POPGUW	TETNIP	XUDXID
BIPDEJ	EAZOBZ	HORCAS	MAPPHI	POPGUX	TETROL	XUFDUY
BIPHEN	EBEKIG	HORREM	MAPWUV	POPJEL	TETTRI	XUJCIO
BIPHME	EBEWOX	HORTAJ	MAPZOS	POPJOV	TETUMS	XULDUD
BIPTEI	EBIDUP	HORTUE	MAQWIM	POPJUA	TETZOL	XULWAE
BIRFEN	EBIGUR	HORXOB	MARBEM	POPLIR	TEVFIK	XUNRON
BIRKIW	EBIRIR	HOTAUR	MATDEQ	POQGOS	TEVJOU	XUNTEH
BIRMEU	EBIXET	HOTZOF	MAVGEW	PORREU	TEVSOD	XUPHEX
BISMEV	EBIZIZ	HOYVEX	MAXDEV	POSCUV	TEYHIQ	XUQSAD
BIVLAT	EBIZUL	HOYVOH	MAXKEB	POSTEW	TEYJEM	XUTCAQ
BIVRAA	EBURCL	HOYWEY	MAXKUT	POVSEY	TEZNUJ	XUTPOR
BIVSIJ	EBYBUA	HOZSAQ	MAZNIM	POXHYN	TFMCYP	XUTPUY
BIXGIY	ECELON	HPCBDP	MAZXBZ	POYGIT	TFMETH	XUVLIJ
BIXKIE	ECITIU	HPHBNZ	MAZXOB	PPDACS	TGLYBE	XUVNIM
BIYBOA	ECITOA	HPTSIO	MBLARA	PRMDIN	TGLYCY	XUVWUH
BIYSEH	ECITUG	HUBSUT	MBPHOL	PROGST	TGLYSU	XUVZET
BIZLOM	ECIVAO	HUCHEU	MBRMAD	PRONAC	THALID	XUYHOO
BIZPUW	ECIVES	HUDCOZ	MBRMET	PROSTA	THBARB	XUZBUQ
BIZWAI	ECIVIW	HUDHUK	MBYINO	PRSTER	THEOPI	XYANAC
BMLHYD	ECOBUU	HUHDUK	MBZANQ	PTBORP	THFUCA	XYLTOL
BNPYRE	ECUVED	HUKHUQ	MBZFBZ	PTCDEC	THHYDT	YABHAM
BOBVEU	EDAPOQ	HUKQEJ	MBZPNA	PTRPHE	THIAMC	YABHEP
BOBVIY	EDAXEM	HUMFIG	MBZYAN	PTRPHT	THIMHC	YABKUI
BOCKEK	EDIGUU	HUMLUW	MCBZAC	PTZTCQ	THIOUR	YACREZ

BOCMOV	EDOPUK	HUMTEP	MCHTEP	PUBMII	THUTEC	YACTEC
BOCPRO	EDOROG	HUPGOO	MEADOB	PUBMUU	THXMAM	YADKEV
BOHZOO	EDOSAT	HUPWAR	MEBQEQ	PUBYAM	THXNOO	YADKUL
BOKBUA	EDOSOH	HURJUZ	MECHLF	PUCMIJ	TIBGAN	YAFQEC
BOKCEJ	EDOTIA	HUSQAN	MECWIC	PUDWUH	TICDIT	YAJFOG
BOKCIP	EDTAXX	HUSQIV	MEDHOU	PUDXES	TICDOZ	YALYUH
BOKDEM	EFAWIT	HUSVIB	MEDLUE	PUDYOC	TICHUI	YAMHID
BOKLAO	EFICOL	HUTQAP	MEFLEQ	PUFSIT	TICNOI	YANDAT
BOKVOM	EFIKOT	HUVVOJ	MEGHAJ	PUGVUK	TIETHE	YANMUW
BOLCUA	EFIKOU	HUWSOI	MEGYON	PUGWUK	TIFDAN	YAPNEK
BOLMIZ	EFINAJ	HUYRIC	MEHROH	PUKMAK	TIFLAV	YAQLEI
BOLNAS	EFINEN	HUZBOT	MEJPIB	PUKVEX	TIHVUC	YAQLIM
BOLNOG	EFINIR	HXACAN	MEKMIZ	PULCOP	TIHWAJ	YAQMAF
BOMDUC	EFOZAB	HXCFUL	MELEZT	PULHUZ	TIHWEN	YAQMIN
BOMKOD	EFUMAU	HXDYNB	MELFIT	PULNUF	TIHYAL	YAQMUZ
BOMKUJ	EFUPEB	HXMACA	MELPUO	PUMRUK	TIHYUF	YAQNEK
BOMWIJ	EFURIH	HXOPEN	MELVEE	PUMVIC	TIJBAQ	YARGON
BOPJIB	EGOPIB	HXOXAM	MELXEG	PUMZII	TIJKIH	YARYAR
BOPKOG	EGUTEF	HXPHOL	MEMTCQ	PUNBAD	TIJSIP	YARZUN
BOPNID	EHEREO	HXQUIN	MENMIB	PUNVIE	TIJSOV	YASGOQ
BOPQAY	EHESAL	HXTACM	MENSEE	PUPBAD	TIJSUB	YASKOS
BOPSAA	EHOPAS	HYDRZN	MEPHCB	PUQGAL	TIJTAI	YAVRIW
BOPSEF	EHOWIH	HYQUIN	MEPVAE	PUQYOQ	TIJTEM	YAWMOY
BOPSIJ	EHULAV	IBBIOX	MEQVAG	PUQZIM	TIJTIQ	YAWPER
BOQCUF	EJEQAL	IBECUO	MERHOG	PURCEM	TIJXUH	YAWPIV
BOQQUT	EJEROA	IBIJAF	METACM	PURFIT	TIJZIV	YAWWIC
BORCUG	EJESIV	IBOBUY	METHOL	PURSEB	TIKBEU	YAXCEF
BORSEG	EJIQEU	IBOCEJ	METMSX	PUVQIG	TIKBOE	YAXCEH
BORVAG	EJUQIK	IBOCOT	MEVVEO	PUVVUX	TIKFAW	YAXDUW
BOSJUP	EKECOM	IBOMAP	MEVVUE	PUXLUP	TIKIND	YAXWAX
BOTCUI	EKIGEK	IBOXAA	MEVXIU	PUZGAT	TILROV	YEBQED
BOVCEV	ELATAN	IBPRAC	MEWFUR	PYAZAC	TINBIB	YEBWIM
BOVWIT	ELEGUY	IBRBPX	MEWHED	PYDCLI	TINJAB	YEFGOG
BOVXOB	ELOHUJ	IBULIA	MEWQOW	PYDPYR	TIPDEB	YEFNEE
BOVYIW	ELOPIE	IBUSIJ	MEXFAW	PYDSYD	TIPHAM	YEFWEM
BOWJII	ELUREJ	IBUXAF	MEXJEE	PYMSUL	TIPHUW	YEGJID
BOWMIK	EMAKUY	IBZFRO	MEYCEA	PYPTCQ	TIPJAE	YEHPOQ
BOWROU	EMAMEL	ICAHIE	MEYGII	PYRAZI	TIPMEP	YEJJUU

BOWWOZ	EMCORR	ICAMOP	MEZKEH	PYRDCI	TIPSAM	YEJLII
BOYFOK	EMISOJ	ICEPOX	MHNPRY	PYRDNA	TIPWIY	YEJNAC
BOZFIF	EMONID	ICEWIW	MHPOAT	PYRDNO-D	TIQKEJ	YEJRUA
BPHBAC	EMPIPP	ICOFEM	MHQACD	PYRENE	TIRVOH	YELHUS
BPHENO	ENAPOZ	ICOLOC	MHXHQU	PYRHCL	TITSIA	YEMZAT-D
BPROMA	ENATAO	ICOMUI	MHXHQV	PYRIDO	TITSOG	YENGAZ
BPXBTZ	ENAZOI	ICOZOR	MIBABA	PYRPIC	TITVAU	YENZUN
BPYZDO	ENICEK	ICUBAL	MICRAT	PYRTCQ	TIVDUZ	YEPCAY
BRACTN	ENIZIK	ICULEY	MIGMUK	PYRZIN	TIVKOZ	YEPHAC
BRASAC	ENVFUR	ICUTOR	MIGQIC	PYRZOL	TIVXUS	YEPRAN
BRESTO	EPEJUF	IDALEF	MIGQOJ	PYZPYT	TIWTAW	YERLAJ
BRFUSO	EPETCQ	IDEPIR	MIGTEC	QABDAZ	TIWYEF	YERRUI
BRMACA	EPITED	IDOBAG	MIHYIL	QABVEU	TIWYIH	YESXUP
BRMANC	EPOPDO	IDONAS	MIHZOU	QADGEH	TIXHAJ	YETPES
BRNAPT	EPUPUB	IFAWAP	MIJHAO	QAGHAI	TIXTUQ	YETPIW
BRNICH	EPZPHX	IFILAL	MILHOF	QAGHEM	TIXVAY	YIBVEL
BROFRM	EQUZIZ	IFIZIG	MINPIJ	QAGLOZ	TIYQAU	YIBVIP
BROXUR	EQZBMI	IFOQAX	MIPBET	QAHGAH	TIZVUT	YICDAR
BRUDAG	ERGCAL	IFOVOO	MIQKOM	QAJQAV	TIZWAA	YICDIZ
BTRILA	ERIXAE	IFULUQ	MIRDEX	QAJTUQ	TMAMMC	YICDUL
BTUPTE	EROXAL	IFUTOT	MIWBAX	QAKREB	TMAMOH	YICFEX
BUCXEC	ESALUF	IFUXEN	MIWHOP	QAKZUY	TMAPCL	YICGEW
BUDKES	ESIVOR	IGALUY	MIXNUD	QALHAN	TMBRBZ	YICJAX
BUDREZ	ESIWEI	IGASIS	MIXPAL	QALPEA	TMDHCB	YICJIF
BUDYOP	ESIWUY	IGUQEG	MIYKOU	QAMNAT	TMESPH	YICNAZ
BUFNEV	ESOFIC	IHAPEO	MIZHOT	QAMQEC	TMETTS	YIDLEC
BUFTED	ESTRON	IHAPOX	MIZPES	QAMQOL	TMOSCA	YIFTIS
BUHNAV	ESTTRD	IHEKOV	MIZPUH	QAMWEH	TMPDPC	YIFYAP
BUJBEN	ETAMBR	IHEMIR	MMANCN	QAMXUY	TMPPIO	YIGPIO
BUJMUO	ETANOL	IHIKIV	MNBZAC	QANHAP	TMURIC	YIHJAA
BULMEA	ETBARB	IHOSUT	MNIAAN	QANTUV	TNBENZ	YIHKAB
BUNDAC	ETBBAR	IHOZOW	MNPHOL	QAPZEN	TNFLUO	YIJDOK
BUNDEC	ETBZIM	IHXNAQ	MNVIMZ	QARXOY	TOBRUX	YIKNEN
BUNKOK	ETCTEP	IJAJUY	MOBBAH	QATCIX	TOCPIM	YILQIV
BUNPEF	ETDIAM	IJAXAT	MOBZAE	QATDIZ	TOHBPA	YIMTUJ
BUNREH	ETEDOX	IJESUL	MOCANT	QATNUU	TOHBUN	YINSAR
BUNSIM	ETEFIT	IJETEW	MOCCOW	QATPAC	TOHHED	YIPFUY

BUPLIH	ETEXIK	IJETOG	MOCYIM	QATPEG	TOHTUG	YIPGEL
BUQQAF	ETEYUX	IJIBEJ	MOCZOU	QAXMEH	TOHVAO	YIPGUB
BURLAC	ETHANE	IJOQED	MODBAJ	QAXPEM	TOKQEP	YIQGEL
BURTIT	ETHBME	IJUDAT	MODNUQ	QAXSUD	TOKSAO	YIQJEN
BURYOC	ETHCTE	IJUDUN	MOHDAP	QAYXAP	TOKSES	YIRVAW
BUTCLT	ETHLEN	IJUMEG	MOHDET	QAYXET	TOLKEK	YIRXAZ
BUTPOV	ETOCEV	IJUXEQ	MOHZOA	QEGBUB	TOLPHO	YISBIM
BUTSIA	ETOJUS	IKOBUF	MOKYER	QEGWEG	TOLPSO	YISJUF
BUVKEJ	EVAZEG	ILIMEV	MOLGIE	QEJGOB	TOLSAM	YIVGIV
BUVZUN	EVEFAN	IMAZOL	MOLXIW	QEJZAH	TOLUEN	YIVHUI
BUYJAH	EVIHUM	IMCYLP	MOMFAX	QEKBEN	TONDUV	YIVRIF
BUYROC	EVUQAO	IMDIAC	MONRAI	QEKDIT	TOQKIV	YIVROL
BUYZUQ	EWACAG	IMEROI	MONSIR	QERJAZ	TORSEM	YIVRUR
BXLAZC	EWAMAR	IMEXUT	MONTIS	QERWAM	TORTEZ	YIWTON
BXZCPN	EWAPAU	IMIPAV	MOPBZA	QESHUS	TOSDAZ	YIXTAB
BZAMID	EWAPIC	IMIYEI	MOPHAA	QESJAA	TOTYIM	YIXTEF
BZANIL	EXAPID	IMPTHA	MOPNAH	QESJEE	TOXGLU	YIXTIJ
BZCHOL	EXCHLN	IMTCOS	MOPPUC	QESJII	TOZKOI	YIXTOP
BZCPTI	EXEKEX	IMUTEQ	MORVAR	QESJOO	TPABAN	YIXTUV
BZDMAZ	EXIBOC	INAHZC	MORVEV	QESZUK	TPASTB	YIXVAD
BZINDA	EXUQUJ	INDANT	MOSDOO	QETZUL	TPCHDA	YIXZOW
BZOFOX	EXZPUN	INDBUA	MOSGEI	QEVXIY	TPCHDB	YIZXIO
BZTRAZ	EYANRO	INDIGO	MOSTIX	QEWJUZ	TPEPHO	YODFAZ
BZTZAD	EYIBEU	INDMET	MOSXIC	QEXBEB	TPHNAP	YOFNAK
BZYACO	EYITAH	INELIW	MOTNUF	QIBQIB	TPHPOR	YOHCON
CABTUV	EYOCUQ	INEQUN	MOVRIY	QICGEP	TPPHSE	YOHKOX
CABWIM	EYOGUV	INIBUC	MOVROE	QICGIT	TPPOSS	YOJFEK
CABZOU	EZAQEB	INOBUI	MOVTIB	QIDVII	TRDECA	YOKSOG
CACBAK	EZEFEV	INOCET	MOVVAV	QIFDIT	TRDMPP	YOKTAT
CACCIS	EZELOK	INODUK	MOVXIF	QIFJAS	TRITAN	YOLDAF
CACDAM	EZIZAP	INOHIC	MOVXOL	QIFKEX	TRIZIN	YOLVUQ
CADYLA	FABDER	INOSIN	MOVYAY	QIFNIE	TRPHAM	YOLYII
CAFORM	FABFUJ	INUKAD	MOVYEC	QIGBEN	TSCARB	YOLYOO
CAGMAY	FABMEA	IOBNZA	MOWDUZ	QIJTOS	TSCPCP	YOLYUU
CAHEXT	FABNUT	IODOFO	MOXSOI	QIJZOY	TTFCAN	YOMXUT
CAHKAX	FABRAB	IPATOJ	MOXVIF	QIKFEV	TTHCTD	YORYAH
CAHSEK	FABRIJ	IPAWAY	MOXVUR	QIKFIZ	TUBDUP	YOTJUN
CAKRUB	FABSUW	IPOPQU	MOYGUC	QIKNUT	TUBRAJ	YOVCOD

CALCLA	FACCET	IPOZAO	MOZSIF	QIKZAN	TUBYIY	YOWXAK
CALOXM	FACCOB	IPUPUE	MOZZEH	QIKZUH	TUCKUZ	YOWYOY
CALQOW	FACNUS	IQACIM	MPBRBL	QILHUO	TUDQEQ	YOYFIB
CAMALD	FACRIK	IQAREY	MPCSZX	QIMCAS	TUDROZ	YOZJUU
CAMPQU	FACSEI	IQIDIW	MPHCQM	QIMGAU	TUHBAZ	YOZSEL
CANDUR	FACZUF	IQILAW	MPIMAC	QIMKIG	TUHXID	YOZZAQ
CANTRQ	FADWUC	IRIMEB	MPIMZR	QIMKOM	TUMDIP	YUBWAV
CANYOG	FAFMOQ	IRUFEH	MPIPXY	QIMXOB	TUMPOG	YUCCIJ
CAPLEK	FAFQEK	IRUQOB	MPPTCQ	QIMXUH	TUPGUH	YUCSIZ
CAPTAZ	FAFWIS	ISALOE	MPTRZS	QIMZOB	TUPRBN	YUFCUZ
CAQYAW	FAGVEO	ISAYEH	MPYAZO	QINCUL	TUQCOX	YUFDAG
CAQYIE	FAHLEF	ISAZOS	MPYRAZ	QIQLIL	TURPUR	YUFDEK
CARBTC	FAHNOR	ISENID	MPYZBD	QIRGIJ	TURPYB	YUFDIO
CARWID	FAHVOZ	ISENOJ	MSAZOP	QIVBAA	TURSAB	YUFDOU
CARZIF	FAJGUS	ISENUP	MSFURY	QIXFUY	TURTEB	YUFDUA
CASHOT	FAJTIT	ISEPAX	MSTEAR	QIXZAY	TUSSIJ	YUFFAI
CASRPP	FAJWAO	ISEPEB	MSULIN	QIYFEJ	TUTBUF	YUFFEM
CATDIM	FAKFUS	ISICUJ	MTHOLI	QIYKAL	TUVXAL	YUFFIQ
CAVCUY	FAKRIS	ISIDEU	MTHXPH	QIYKEP	TUWFIB	YUFFOW
CAVJAM	FAMDIG	ISUNAL	MTKETZ	QIYKEQ	TUWHEZ	YUFGAJ
CAVJIU	FANDOO	ISUQOC	MTPETA	QIYKIT	TUXJIF	YUFGEN
CAVVUQ	FAQTIA	ITUYAX	MUCKUR	QIYKOZ	TZAZPC	YUFGIR
CAWDUZ	FARPOF	IURACL	MUCNOO	QIZHIQ	UBABAC	YUFVOL
CAXHIS	FARRUM	IVADUE	MUDWEO	QNACRD	UBEDUA	YUHGOX
CAXMOD	FARWIF	IVEJUO	MUFBOE	QNGHSU	UBEFEO	YUHHUE
CAXWUT	FAWFOY	IVOBOK	MUFMAB	QOCDOB	UBEJIW	YUJGIT
CAZFUE	FAWSAZ	IVOLEL	MUFNEG	QOCNAX	UBUJIM	YUJJOE
CAZLAR	FAWVOO	IVUQOF	MUHDUO	QOCNIF	UBUQIR	YUKGUG
CAZTBZ	FAXBUB	IVURAS	MUJTOB	QODGUM	UBUREO	YUKPAW
CBENPH	FAXPOJ	IWEJOK	MUKNIQ	QODQUV	UCAYED	YULGIW
CBFBZF	FAXVAB	IWOCON	MULFOO	QODTAE	UCAYIH	YULNUO
CBMZPN	FAXVOQ	IWUKAM	MUNMOY	QODTIO	UCECAG	YUMHOE
CBZCAN	FAYFOA	IXEYIT	MUNTOG	QODZUG	UCENOG	YUMKOH
CBZTCQ	FAYFUG	IXOGAD	MUPPES	QOGNEF	UCEQID	YUWZEW
CBZYTO	FAYNUO	IXOYUQ	MUPQET	QOGREJ	UCIJOF	YUXCAV
CEBGOF	FAYVOQ	IYAWIO	MUROXA	QOHBEV	UCITEF	YUXCEZ
CEBGUL	FAYVUW	IYEBUJ	MUTZOQ	QOHSAJ	UCUGOP	YUXFIH
CEBKEZ	FAZTAC	IYEJIF	MUWSON	QOHYAO	UDAYUT	YUYDOL

CECPAB	FBPAZD	IYUXUW	MUWTED	QOJNOU	UDAZII	YUYHIJ
CEDGAV	FECHOM	IZANEC	MUXNUO	QOLVOD	UDELEV	YUYMIO
CEDXUE	FECZOE	IZANOM	MUYROO	QOPBED	UDETOM	YUYPAJ
CEHQEM	FEFKIL	IZAPEE	MUYSOP	QOPSEU	UDEVOO	YUYPUD
CEJWUJ	FEFKIM	JABNIJ	MXBIOX	QOPZUQ	UDIHOE	ZADREB
CEKJAD	FEFPAI	JABPEH	MXBTCP	QOQBAZ	UDIPOM	ZADRIF
CEKKOU	FEFPEM	JABPOR	MXPBUQ	QOQVOH	UDOXAN	ZADWOQ
CELBEA	FEFQUD	JABSAG	MYINOL	QOQYEC	UFAGIS	ZAJGUM
CELHIL	FEFREP	JABXOZ	NAAMPH	QOTBIM	UFAMIY	ZAKMIH
CELQUH	FEFSAK	JABZAN	NABUOX	QOTGOV	UFEBIS	ZAPFED
CELRAO	FEFXOF	JACREL	NABWOD	QOTHEM	UFEZAH	ZAQZUM
CELRIW	FEGTEQ	JADNOS	NACVEU	QOTMUI	UFIKAX	ZASQOZ
CELWIB	FEGWAP	JAFGUS	NACVIY	QOVREZ	UFOCAU	ZAVVUN
CEMCED	FEHKOS	JAGJEG	NADHOP	QOYGUH	UFOCIC	ZAXKUE
CEMDON	FEHWAR	JAGREP	NADQAL	QOYJOD	UFOGON	ZAYKAL
CEMQOA	FEJNOZ	JAHKUZ	NAFZUP	QOYKIY	UGAKUK	ZAYKOZ
CENGEH	FEMMAL	JAKGEH	NAGGEH	QOYNOH	UGEDOA	ZBCNON
CENNOA	FENPUJ	JAKKUB	NAGHOT	QQQACY	UGIFOG	ZEBVEJ
CENRIW	FENTEX	JAKQAO	NAGTEU	QQQAKS	UGOVIX	ZEBWEK
CEPXHP	FENXUR	JAKYOJ	NAHWIE	QQQAPG	UGOXEU	ZECZIQ
CERBEG	FEPHAL	JALBII	NAHWOK	QQQAUG	UGOXIY	ZEDGAQ
CERJAM	FEPJOB	JAMRIY	NAJBEF	QQQAUJ	UHAVAB	ZEDPIH
CERLOA	FEPROJ	JAPGUC	NAJDOS	QQQAXG	UHAVEG	ZEDPON
CERYAA	FEQDIQ	JAPHAJ	NAKCYB	QQQAXJ	UHAWEF	ZEFFEX
CESCNM	FEQFEN	JAQJAN	NAKQOG	QQQAZJ	UHAWIJ	ZEFXIR
CETRUQ	FEQPAS	JARPEX	NAKQUM	QQQBDJ	UHENUQ	ZEGLUS
CEVPOK	FEQVUT	JARQUP	NALCYS	QQQBLM	UHIVEN	ZEGMAZ
CEVWAC	FESCOW	JARXAB	NALIDX	QQQBNG	UHUHOU	ZEGSUZ
CEWGAM	FESKAP	JARXUV	NANQUO	QQQBNV	UHULEQ	ZEKPEM
CEWVOP	FESNEW	JATFUF	NAPACA	QQQBRD	UHUMEP	ZEKPIQ
CEXDAK	FETPIF	JAVCEO	NAPHAC	QQQBTP	UHURAR	ZEKPOW
CEZDOC	FEVBUD	JAVWIN	NAPHOB	QQQBVP	UJACAL	ZELDOJ
CHAPEP	FEVCAK	JAWQIH	NAPHYZ	QQQCIG	UJACIT	ZEMXAQ
CHIPTH	FEVKAT	JAWQUT	NAPPYR	QQQCIV	UJIRIO	ZEMXOE
CHLSAN	FEVMUO	JAWXEK	NAPTAN	QQQCVG	UJODUT	ZENQDX
CHNOCH	FEVNAV	JAXGUK	NAPYDH	QQQCYA	UJOGUW	ZENVAP
CHOCHL	FEVWUY	JAYCES	NAPYMA	QQQCZS	UJORIU	ZEPDAZ
CHOLEC	FEXHEW	JAYPUU	NAQCUF	QQQDDS	UKAKEW	ZEPFAB

CHOLES	FEXHOH	JAZCOD	NAQRIG	QQQDHD	UKANOJ	ZETSEW
CHOLEU	FEXRIK	JEBFEB	NARSOP	QQQDQY	UKIFIE	ZETZOP
CHOLSI	FEYBIU	JEDTOB	NATCNQ	QQQDVM	UKOSAP	ZEXHIV
CHXAMH	FEYGIZ	JEFDON	NATLUP	QQQDZG	UKOYUO	ZEXJAP
CIBZAQ	FEYLUQ	JEFDUT	NATMAW	QQQEJV-D	UKUQOH	ZEXLUJ
CICYES	FEZGUM	JEGLAI	NATNIF	QQQFAA	ULALUO	ZEXPEX
CICYOD	FIBKUW	JEHFIL	NAVCIX	QQQFAD	ULAWAF	ZEXQUO
CIDFEC	FIBYIY	JEJSIA	NAVGAT	QQQFBP	ULERAE	ZEXREZ
CIDXUI	FICGEE	JEKHEN	NAVSUY	QQQFDJ	UMOMAL	ZEXRIF
CIFDOK	FIDYIA	JEMNUK	NAWSUB	QQQFDS	UNDEAC	ZEXXOP
CIFLEI	FIFBEB	JEMROJ	NAXDIZ	QQQFDY	UNEVOZ	ZEXZIN
CIGHEG	FIFCEC	JEMRUP	NAXFUO	QQQFED	UNEWIU	ZEYBIO
CIGKAF	FIFFAB	JEMRYY	NAXVOY	QQQFES	UNEWUF	ZEYDEO
CIKDIK	FIFGOQ	JEMSAW	NAYHUR	QQQFGD	UNEYIV	ZEYHUG
CILHIO	FIGYID	JEPMIA	NAYNUX	QQQFHJ	UNEZAO	ZIGPAG
CILJIQ	FIHNUH	JEPMIB	NAYQEJ	QQQFVD	UNIRUF	ZILHOR
CIMETD	FIHYEA	JETKID	NAZHON	QQQGMA	UNISEQ	ZILNOX
CIMMUI	FIJBUW	JETLEA	NAZLAC	QQQGTS	UNISIU	ZIMHIO
CINCUY	FIKFIO	JEVSOS	NAZQEL	QQQGVV	UNOGIN	ZIRGUC
CINMAC	FIKJEQ	JEXBIY	NBARBT	QQQHDS	UPOYEE	ZIVGEQ
CINMER	FIKZOO	JEXKUS	NBUTOL	QUBPAF	UQOBIM	ZIWHOC
CINNDS	FILBEH	JEYDEW	NBZANO	QUBPIN	URAQEK	ZIWHUI
CINYEE	FILGEM	JEYWEQ	NBZOAC	QUCWAM	URAZIX	ZIWJAQ
CIPJAM	FILHAJ	JIBCIG	NBZSES	QUFDUQ	UREAXX	ZIWVIK
CIPPAT	FILYAA	JICTUK	NDOCLH	QUFFAY	USIZAY	ZIWYUZ
CIPYAB	FIMDOU	JIDTOF	NEBGUX	QUFHAA	UTIXAX	ZIYWAH
CIRDIS	FIMGIR	JIKDEM	NECNEO	QUIDON	UTORAX	ZOBBEZ
CIRTEC	FIMNAQ	JILZOU	NECWEX	QUJDOP	UTORIF	ZOCPUE
CISHOC	FIMTUQ	JIMVUW	NECYAX	QUKJIR	UVEXID	ZODJEH
CISJEV	FIMVAY	JINGIW	NEDMUF	QUKVUN	UWUKAZ	ZODWIY
CISQAW	FINZIL	JINROO-D	NEDNOZ	QULLUF	UXAGAC	ZOGQAN
CITRIG	FINZOR	JINROO-H	NEDZEB	QUMFEJ	UXEGEK	ZOKHAK
CITROM	FIRXUA	JINVOR	NEFCEG	QUNDIO	UXIXAB	ZOMDEM
CIVTUW	FISSUV	JIPJAT	NEFQEW	QUPHEN	UXIXIJ	ZOPTED
CIWJIB	FITQII	JIPJEX	NEGCUY	QURWOQ	UXIYIK	ZOQZIQ
CIWMUS	FIVGAS	JIRCAP	NEHJER	QUSQEB	UXIYOQ	ZOSLAU
CIWVAH	FIVSIM	JISFUM	NEHKIV	QUYLEC	UXIZAD	ZOWSUZ
CIXCES	FIWZUG	JISLUT	NEHREZ	RACLIS	UYAZEA	ZOXHAX

CIXKEA	FIYBEV	JISWOY	NEJHIU	RADLAK	UYOYIR	ZOYLII
CIYFIB	FIYFAU	JITNOP	NEKFIT	RAFFAF	UZENIX	ZOYMOP
CIYRIL	FIYTIQ	JIVXES	NEMLOG	RAFFIO	UZOVAH	ZUDTAT
CIYXAK	FIYTOW	JIWPEL	NEMYIO	RAGLIV	VACTAU	ZUDTAV
CIZDUK	FIYTUC	JIWPOV	NENFIX	RAHSUO	VAFCEK	ZUHGOY
CIZGUN	FIYVAK	JIWQAI	NENGAP	RAKCAJ	VAGDOX	ZUHKAO
CLACAM	FIZPEL	JOFWIM	NENGET	RAKMEX	VAGPOJ	ZUHQAU
CLACET	FIZQOW	JOHGEU	NENJOF	RAKWIJ	VAHTAB	ZUHRID
CLAMPL	FIZRUD	JOHJIB	NENNUP	RALMOG	VAKPUU	ZUHTOL
CLBZAM	FIZVUF	JOJYOY	NEPGCL	RALQIE	VAKTOS	ZUHXAB
CLBZNT	FIZWOC	JOKWEM	NEPTAZ	RAMGOB	VALIDL	ZULGEU
CLFORM	FLUBIP	JOLCOE	NEQHAT	RAMKIZ	VALINM	ZUMPUS
CLMCBU	FLYITU	JOPJAZ	NEQHEX	RAMNUP	VALINO	ZUMXIQ
CLNPHT	FMDURD	JOQVAO	NETCUM	RAPTEI	VALLIE	ZUNHOH
CLPHOL	FNAPTH	JORGOO	NETKOM	RAQDIW	VALPII	ZURMEG
CLPHTE	FOBBIH	JORMAE	NEWYET	RAQTIN	VALSUY	ZUSLIK
CLPMCY	FOBCAA	JOTNAH	NEXPAH	RARXEP	VALTEJ	ZUWTOA
CLPXAD	FOBCAB	JOWCAB	NEXPEL	RASTIO	VAMBOA	ZUZYAU
CLURAC	FOBMIU	JOWGAF	NEYLEI	RASTOU	VAMZIT	ZZZAKJ
CMHXDC	FOBSOE	JOWNAM	NIBGOU	RASXIS	VAMZOZ	ZZZAKP
CMPIXZ	FOCWUR	JOWPAO	NIBVUQ	RATVIR	VAMZUF	ZZZAKV
CNITBZ	FOFZEH	JOWXUO	NIBZAL	RAVMUX	VANXAK	ZZZAOV
COBBIE	FOGVIG	JOWZIG	NICOAM	RAXRAK	VAPKIG	ZZZAUS
COBJIM	FOHLIY	JOYHEK	NICSAL	RAYCID	VARCIA	ZZZAVM
COBKIN	FOJGAM	JOYJAK	NIDPIB	RBHACD	VARFAX	ZZZAWJ
COBWOF	FOJLUL	JUBFOB	NIFGAM	RBHTCA	VARKII	ZZZAWV
COCGEG	FOJMAS	JUBFUH	NIFHIT	RBTCNQ	VARNEH	ZZZAYP
COCGOQ	FOJMOG	JUDRAB	NIFMAS	REBGAG	VARYES	ZZZBCS
COCHEJ	FOJRAX	JUDRIJ	NIGWEH	REBYEE	VARYIW	ZZZBFM
COHQUN	FOLBUE	JUGVUE	NIJHUJ	RECQOH	VASXES	ZZZBLP
COHWIF	FOLPEB	JUHGAU	NIKSUV	REHGUI	VATRIT	ZZZBPD
COHZAA	FOMGIY	JUHJIF	NILLEB	REHLAT	VATSAK	ZZZDDJ
COKBIN	FOMNAX	JUHLUT	NILWIO	REHTII	VATSOA	ZZZDDP
COKVON	FOMNEB	JUHNUV	NIMFOE	REJNUP	VATXOF	ZZZEEU
COLDOX	FOMVIO	JUKMEJ	NIMNED	REKBUE	VAWDOM	ZZZFEE
COLTAY	FONFAQ	JULBUN	NIMRIK	RENSEJ	VAWKUA	ZZZFLM
COMPEA	FOPBAO	JULHED	NIPHSS	REPFOH	VAXTIZ	ZZZGMW
COMXAD	FOQJEC	JULWUI	NIPLED	REPWUG	VAXXOH	ZZZHKQ

COPTUW	FOQZAM	JULZAR	NIPSOV	REPXER	VAXZIF	ZZZHQU
COQJOH	FORBZA	JUSBUU	NIQFAV	RESORA	VAYKUB	ZZZHUW
COQNUR	FORMAC	JUVJAL	NIQZET	RESZUK	VAYSET	ZZZHWI
COQRUV	FOSFUQ	JUZBUB	NIRDUO	REVCEB	VAZCOP	ZZZIYE
COQWOU	FOSMIL	KACRUC	NIRJII	REVCIF	VAZRIX	ZZZIZM
CORDCP	FOTYUI	KADFAY	NIRTAJ	REWDOM	VAZRIY	ZZZJIQ
CORDIW	FOVBIC	KADNEI	NISLOQ	REWPUE	VEBBUA	ZZZKAO
CORSIL	FOVNOT	KADPOU	NISNAF	REYDEE	VEBCAH	ZZZKDW
CORTEI	FOVRUE	KADTEO	NITPOL	REYTEU	VECRUR	ZZZKGE
CORYIR	FOVYOE	KADZEU	NITRIR	REZNAL	VECXIK	ZZZKPE
COSDAR	FOWPIR	KADZIY	NIVPAJ	REZRIY	VECXUW	ZZZLBS
COTSAF	FOWZUN	KAHHAC	NIVTAP	REZRIZ	VECYAD	ZZZLGU
COTXUG	FOXLOT	KAHLEK	NIWFEE	RIDFOA	VEDQOL	ZZZMAI
COTYEP	FOXNAH	KAHQOA	NIWJUA	RIDJUJ	VEFLIB	ZZZMBE
COVJON	FOXNEL	KAHRIW	NIWMEN	RIFBAL	VEFWOS	ZZZMGS
COVZOE	FOXNUB	KAHTEU	NIXBEB	RIFMUQ	VEFYAG	ZZZMJK
COWRIP	FOXREQ	KAHTUK	NIYBUU	RIFQAY	VEGHIY	ZZZMMG
COXDHN	FOYNEO	KAHWEX	NIYDUU	RIGHUK	VEGMAV	ZZZMRW
COYLAF	FOZVOF	KAJQOB	NIYLUC	RIGVEJ	VEGNAX	ZZZMUC
COYMOS	FPAMCA	KAJROD	NIZVAU	RIHVUZ	VEJCIW	ZZZNNM
CPAHYD	FPYRMO	KAKBAZ	NIZVEY	RIMKAB	VEJXAJ	ZZZNQQ
CPHAZO	FSEANT	KAMCOQ	NMACEP	RINDID	VEKSOT	ZZZNQS
CPPHEN	FUCKOD	KAMHAJ	NMBYAN	RINTEN	VEKYAL	ZZZNRU
CRESOL	FUCTIG	KANQAR	NMHCOU	RIPBOH	VEMTOW	ZZZNUK
CRETAL	FUDTON	KANYUU	NMZNON	RIRZIC	VENMUW	ZZZNYY
CSIXPH	FUFJAT	KAPVIH	NOBHES	RISHIM	VENVIT	ZZZODM
CTBROM	FUFNOJ	KAQGAM	NOCZUB	RISZOK	VEQNEK	ZZZODU
CTCYME	FUFSIJ	KARCOW	NOETNA	RITBED	VERNOV	ZZZOEG
CTMTNA	FUFTEG	KARGUF	NOGUNA	RITNOY	VESTIX	ZZZOFC
CTPHMI	FUFXOV	KASKUL	NOHTOT	RIVFAE	VETVOG	ZZZOPV
CTVHVH	FUGJUM	KASSOM	NOJHEZ	RIWTAT	VEVLIR	ZZZOQK
CUBHUE	FUGMIF	KASXUY	NOLFUP	RIWTEX	VEXJEN	ZZZOYC
CUCKOA	FUGTIK	KAWFAQ	NONFOM	RIWTIB	VEXKEO	ZZZPLY
CUCSEY	FUGYAH	KAWJAU	NONJAD	RIWWEA	VEXNUI	ZZZPMI
CUFKOE	FUMAAC	KAWYOW	NOPGUU	RIYTUP	VEYGEL	ZZZPNG
CUGJAP	FUPDOJ	KAXGEW	NOPHKN	RIZQOG	VEYVEA	ZZZPPI
CUHDAK	FUQJIK	KAXHAS	NOQGIJ	RIZXON	VHVIMH	ZZZPRC
CUHTOO	FUQLIM	KAXWAJ	NORDII	ROBKEY	VIBKIA	ZZZPRO

CUKCAM	FUQMIO	KAXXAI	NORFUX	RODLIG	VIBMOJ	ZZZPUS
CUKCIU	FUQMOT	KAYVAH	NORVEX	ROFKAZ	VICPEC	ZZZPZE
CUKHUM	FUQMOU	KAYWIR	NOSWAT	ROFVAK	VICQAB	ZZZQJS
CUKJEY	FUQMUZ	KAZDUL	NOTEST	ROJROZ	VIDMAX	ZZZQNK
CUMMIG	FUQWAQ	KCAPRA	NOVNIX	ROKBOI	VIFGIA	ZZZQSW
CUMTAF	FURACL	KCHLOR	NOVQOG	ROKCEZ	VIFLON	ZZZSBA
CUNCOF	FURANE	KDGLUM	NOVSIA	ROKQUF	VIFQIL	ZZZSHS
CUNKAY	FURHUV	KEBGAB	NOZWII	ROLFEE	VIHGID	ZZZSSY
CUNKUS	FURSEM	KEBHIK	NOZWOO	ROLGEE	VIJGAY	ZZZTTC
CUNPUW	FURWIA	KEBWUK	NTBZAM	ROPQOC	VIJWEQ	ZZZUCY
CUPQUB	FUSWOH	KECYBU	NUDDIZ	ROPQUI	VIMJIM	ZZZUOQ
CUPREM	FUWWOL	KEDDAY	NUDGEZ	RORNIV	VIMPAJ	ZZZUQO
CUQZAP	FUYJAK	KEFYOK	NUGKIL	ROZRAB	VIOLME	ZZZUWK
CURJEE	FUYVUQ	KEGTAS	NUGZEV	RUCJUV	VIPBED	ZZZUXA
CUVSIV	FUYYON	KEKVEC	NUHZEX	RUFBAW	VIPKIO	ZZZUYA
CUVTAO	FUYZEE	KELFOX	NUJGUV	RUFGUV	VIQFUY	ZZZVBQ
CUYQUI	FUZMET	KELGEO	NUJKEJ	RUFHIK	VIRAZL	ZZZVCO
CUZKOX	FUZPUL	KELNIA	NUJLIN	RUGDUS	VISKAJ	ZZZVTY
CYACAC	GACFOG	KEMBUA	NUJPIT	RUGFII	VISQOF	ZZZVUY
CYANAC	GADGUN	KEMDEM	NUJTUI	RUGSIW	VISSOF	ZZZVXQ
CYCHEX	GADHAU	KEPXAE	NUKMAH	RUKDAD	VITAAC	ZZZVYE
CYCLAM	GADSIO	KERFOC	NUKPAK	RUKFIO	VITMAN	ZZZWEO
CYPRCA	GADTOV	KERNAW	NUKVOF	RUKGUZ	VITQUM	ZZZWEQ
CYTDEC	GADVUD	KERNEA	NUKWEW	RUKHAG	VITROH	ZZZWGK
CYTIAC	GAFFAV	KESNEC	NUKXEX	RULKUG	VIVQOH	ZZZWNG
CYTSIN	GAFLAA	KESYAJ	NUKXUN	RUNGEN	VIVRIB	ZZZZCA
CYVTPA	GAFPAE	KETDIW	NUKYUO	RUPFIS	VIVTAW	ZZZZCB
DABGUI	GAFPEI	KETDUI	NULTIY	RUPTIH	VIWHAM	

Table S4 contains all refcodes for compounds deemed to be polymorphic, whether Class A (green) or Class B (pink). The crystal types are listed for each compound. The percentages of each type of crystal for each class are shown in Figure 1 in the main text. Refcodes with an asterisk indicate those which had only one entry flagged as polymorphs (blue in Table S3) but were determined to be polymorphic compounds having two forms with 3D coordinates determined.

Table S4 List of refcodes (3,461) and crystal types for compounds determined to display polymorphism

Refcode	Crystal Type
ABADIS	ANHYDRATE
ABAKEV	ANHYDRATE
ABAZOS	COCRYSTAL
ABEFUJ	ANHYDRATE
ABEKUN	COCRYSTAL
ABIHIC	ANHYDRATE
ABIKEB	ANHYDRATE
ABUDAD	ANHYDRATE
ABUNIU	COCRYSTAL
ACAFEQ	ANHYDRATE
ACAMUN	ANHYDRATE
ACARBM	SALT
ACBNZA	ANHYDRATE
ACDECT	ANHYDRATE
ACEDAN	ANHYDRATE
ACEMID	ANHYDRATE
ACETAC	ANHYDRATE
ACICAR	ANHYDRATE
ACIGID	ANHYDRATE
ACOMUC	SOLVATE
ACONIR	COCRYSTAL
ACOYOG	COCRYSTAL
ACPRET	ANHYDRATE
ACPYRI	ANHYDRATE
ACRDIN	ANHYDRATE
ACRLAC	ANHYDRATE
ACSALA	ANHYDRATE

Refcode	Crystal Type
MBRMET	ANHYDRATE
MBYINO	ANHYDRATE
MBZFBZ	SOLVATE
MBZPNA	ANHYDRATE
MBZYAN	ANHYDRATE
MCBZAC	ANHYDRATE
MCHTEP	ANHYDRATE
MEADOB	SALT
MEBQEQ	ANHYDRATE
MECWIC	ANHYDRATE
MEDHOU	SALT
MEDLUE	ANHYDRATE
MEFLEQ	ANHYDRATE
MEGHAJ	ANHYDRATE
MEGYON	ANHYDRATE
MEHROH	ANHYDRATE
MEJPIB	ANHYDRATE
MEKMIZ	ANHYDRATE
MELEZT	HYDRATE
MELFIT	ANHYDRATE
MELPUO	ANHYDRATE
MELVEE	ANHYDRATE
MELXEG	ANHYDRATE
MEMTCQ	SALT
MENMIB	ANHYDRATE
MENSEE*	ANHYDRATE
MEPVAE	ANHYDRATE

ACTART	SALT
ACTOLD	ANHYDRATE
ACULIV	ANHYDRATE
ACUMIU	ANHYDRATE
ADAJUK	ANHYDRATE
ADIPAC	ANHYDRATE
ADIPAM	ANHYDRATE
ADMNTB	ANHYDRATE
ADPOSM	HYDRATE
ADULEQ	COCRYSTAL
ADURIA	ANHYDRATE
AFAZEM	ANHYDRATE
AFECUJ	ANHYDRATE
AFIKEH	ANHYDRATE
AFIPIQ	ANHYDRATE
AFLATM	ANHYDRATE
AFORIY	SALT
AFUWED	HYDRATE
AFUYIJ	ANHYDRATE
AGAKUP	ANHYDRATE
AGUAHP	SALT
AHADAQ	ANHYDRATE
AHAPEG	ANHYDRATE
AHEGEZ	ANHYDRATE
AHEJAZ	ANHYDRATE
AHEMAB	ANHYDRATE
AHICIF	ANHYDRATE
AHOFAE	ANHYDRATE
AHONUG	SALT HYDRATE
AHOXLH	SALT HYDRATE
AJAJEA	COCRYSTAL
AJAPOR	ANHYDRATE
AJETAL	ANHYDRATE
AJEYAQ	ANHYDRATE
AKEMIN	SOLVATE
AKOVOL	ANHYDRATE

MEQVAG	ANHYDRATE
MERHOG	ANHYDRATE
METHOL	ANHYDRATE
MEVVEO	ANHYDRATE
MEVVUE	SOLVATE
MEVXIU	SALT HYDRATE
MEWHED	ANHYDRATE
MEWQOW	ANHYDRATE
MEXFAW	ANHYDRATE
MEXJEE	ANHYDRATE
MEYCEA	SALT
MEYGII	ANHYDRATE
MEZKEH	ANHYDRATE
MHNPRY	ANHYDRATE
MHPOAT	ANHYDRATE
MHQACD	ANHYDRATE
MICRAT	ANHYDRATE
MIGMUK	ANHYDRATE
MIGQIC	ANHYDRATE
MIGQOJ	SALT
MIGTEC	COCRYSTAL
MIHZOU	SALT
MIJHAO	ANHYDRATE
MILHOF	ANHYDRATE
MINPIJ	SALT
MIPBET	ANHYDRATE
MIQKOM	ANHYDRATE
MIRDEX	ANHYDRATE
MIWBAX	ANHYDRATE
MIWHOP	ANHYDRATE
MIXNUD	ANHYDRATE
MIXPAL	ANHYDRATE
MIZHOT	ANHYDRATE
MIZPES	SALT
MIZPUH	ANHYDRATE
MMANCN	ANHYDRATE

ALABUK	ANHYDRATE
ALADIB	SOLVATE
ALAHEA	SOLVATE
ALIBUS	ANHYDRATE
ALOVUS	ANHYDRATE
ALOXAN*	ANHYDRATE
ALUQEE	COCRYSTAL
AMBACO	ANHYDRATE
AMBNAC	ANHYDRATE
AMBNZA	ANHYDRATE
AMBZPH	ANHYDRATE
AMHCLA	SALT
AMIRIY	ANHYDRATE
AMNTPY	ANHYDRATE
AMPETS	SALT
AMTCAR	SALT
AMXBPM	ANHYDRATE
AMYTAL	ANHYDRATE
ANISAC	SALT
ANLINB	SALT
ANONEX	ANHYDRATE
ANTCYB	COCRYSTAL
ANUCES	SOLVATE
ANUKAW	ANHYDRATE
ANUMEC	COCRYSTAL
ANUMIG	COCRYSTAL
APATOB	ANHYDRATE
APENTN	ANHYDRATE
APESOE	SALT SOLVATE
APGPAL	ANHYDRATE
APLYSU	ANHYDRATE
APUDEV	ANHYDRATE
APUSAF	ANHYDRATE
APUTUB	ANHYDRATE
APYRDN	SALT
AQOMAU	HYDRATE

MNBZAC	ANHYDRATE
MNIAAN	ANHYDRATE
MNPHOL	ANHYDRATE
MOBBAH	ANHYDRATE
MOBZAE	SALT
MOCCOW	COCRYSTAL
MOCZOU	SALT
MODBAJ	SALT
MODNUQ	ANHYDRATE
MOHDAP	ANHYDRATE
MOHDET	ANHYDRATE
MOHZOA	ANHYDRATE
MOKYER	ANHYDRATE
MOLGIE	SALT
MOLXIW	SALT HYDRATE
MOMFAX	SALT
MONRAI	ANHYDRATE
MONSIR	ANHYDRATE
MONTIS	ANHYDRATE
MOPBZA	ANHYDRATE
MOPNAH	ANHYDRATE
MOPPUC	ANHYDRATE
MORVAR	ANHYDRATE
MORVEV	COCRYSTAL
MOSDOO	ANHYDRATE
MOSGEI	SALT
MOSTIX	ANHYDRATE
MOSXIC	ANHYDRATE
MOTNUF	ANHYDRATE
MOVTIB	HYDRATE
MOVVAV	ANHYDRATE
MOVXIF	ANHYDRATE
MOVXOL	ANHYDRATE
MOVYAY	ANHYDRATE
MOVYEC	ANHYDRATE
MOWDUZ	COCRYSTAL

AQOSIJ	ANHYDRATE
ARUWUG	ANHYDRATE
ASATET	ANHYDRATE
ASEHUB	ANHYDRATE
ASEQEU	SALT
ASETOI	ANHYDRATE
ASITEC	ANHYDRATE
ASIXEF	SOLVATE
ATABAZ	ANHYDRATE
ATAGAD	ANHYDRATE
ATAWIC	ANHYDRATE
ATCPEN	ANHYDRATE
ATDZSA	ANHYDRATE
ATEBIK	SALT HYDRATE
ATIDOX	SALT
ATIWOP	ANHYDRATE
ATPRCL	ANHYDRATE
AVIHIX	ANHYDRATE
AVIWEH	SOLVATE
AVIYEK	ANHYDRATE
AVOJUR	ANHYDRATE
AVUFED	SALT HYDRATE
AWAKIS	ANHYDRATE
AWAXOM	COCRYSTAL SOLVATE
AWAYAZ	ANHYDRATE
AWAYON	ANHYDRATE
AWELOE	ANHYDRATE
AWEWAB	ANHYDRATE
AWIFUI	ANHYDRATE
AWIHOE	COCRYSTAL
AWIHUK	ANHYDRATE
AWUNOV	ANHYDRATE
AWUWIY	SALT HYDRATE
AXAJOZ	ANHYDRATE
AXOGAW	ANHYDRATE

MOXSOI*	COCRYSTAL
MOXVIF	COCRYSTAL
MOZSIF	ANHYDRATE
MOZZEH	ANHYDRATE
MPBRBL	ANHYDRATE
MPHCQM	SALT
MPIMZR	ANHYDRATE
MPIPXY	ANHYDRATE
MPTRZS	ANHYDRATE
MPYAZO	ANHYDRATE
MSAZOP	SALT
MSFURY	ANHYDRATE
MSTEAR	ANHYDRATE
MSULIN	ANHYDRATE
MTPETA	ANHYDRATE
MUCKUR	SALT
MUDWEO	ANHYDRATE
MUFBOE	SALT
MUFMAB	ANHYDRATE
MUHDUO	SALT
MUJTOB	ANHYDRATE
MUKNIQ	ANHYDRATE
MULFOO	COCRYSTAL
MUNMOY	SOLVATE
MUNTOG	ANHYDRATE
MUPPES	SOLVATE
MUROXA	COCRYSTAL
MUWSON	SALT
MUXNUO	HYDRATE
MUYROO	ANHYDRATE
MUYSOP	ANHYDRATE
MXBIOX	ANHYDRATE
MYINOL	ANHYDRATE
NABUOX	SALT
NABWOD	SALT

AXUBUR	ANHYDRATE
AXUCEC	ANHYDRATE
AXUWAS	ANHYDRATE
AYIBOZ	SALT
AYOVUG	ANHYDRATE
AZADAG	ANHYDRATE
AZAFAJ	ANHYDRATE
AZAJAN	ANHYDRATE
AZBNBT	ANHYDRATE
AZBZCX	ANHYDRATE
AZELAC	ANHYDRATE
AZERIH	ANHYDRATE
AZERON	ANHYDRATE
AZESEE	ANHYDRATE
AZETAB	ANHYDRATE
AZIBYR	ANHYDRATE
AZOMAD	ANHYDRATE
AZSTBA	ANHYDRATE
AZUGIM	ANHYDRATE
BAAANL	ANHYDRATE
BACRIH	SALT
BADTEX	SALT HYDRATE
BAFLID	ANHYDRATE
BAGFIY	ANHYDRATE
BAHFAR	SALT
BAHNUU	ANHYDRATE
BAJYIU	ANHYDRATE
BAKQEL	ANHYDRATE
BALNIN	ANHYDRATE
BALWEQ	SALT
BANGOM	ANHYDRATE
BANHOO	ANHYDRATE
BANJEH	ANHYDRATE
BANQOX	SALT HYDRATE
BAPBOJ	ANHYDRATE
BAPLOT	ANHYDRATE

NADHOP	ANHYDRATE
NADQAL	ANHYDRATE
NAFZUP	ANHYDRATE
NAGGEH	ANHYDRATE
NAGHOT	ANHYDRATE
NAGTEU	SOLVATE
NAHWIE	ANHYDRATE
NAHWOK	ANHYDRATE
NAJBEF	ANHYDRATE
NAKCYB	SALT HYDRATE
NAKQOG	ANHYDRATE
NAKQUM	ANHYDRATE
NALCYS	ANHYDRATE
NALIDX	ANHYDRATE
NANQUO	ANHYDRATE
NAPACA	ANHYDRATE
NAPHYZ	ANHYDRATE
NAPTAN	ANHYDRATE
NAPYMA	COCRYSTAL
NAQCUF	ANHYDRATE
NAQRIG	ANHYDRATE
NARSOP	COCRYSTAL
NATCNQ	SALT
NATMAW	ANHYDRATE
NATNIF	COCRYSTAL
NAVGAT	ANHYDRATE
NAVSUY	ANHYDRATE
NAXDIZ	ANHYDRATE
NAXFUO	ANHYDRATE
NAXVOY	SALT
NAYHUR	ANHYDRATE
NAYNUX	ANHYDRATE
NAYQEJ	ANHYDRATE
NAZHON	ANHYDRATE
NAZLAC	ANHYDRATE
NAZQEL	ANHYDRATE

BAQBEA	ANHYDRATE
BAQHOS	ANHYDRATE
BAQJEK	ANHYDRATE
BARBAC	ANHYDRATE
BARBAD	HYDRATE
BARWUM	ANHYDRATE
BASVUM	ANHYDRATE
BATWOI	ANHYDRATE
BATWUP	ANHYDRATE
BATZAZ	SALT
BAWNIW	ANHYDRATE
BAWSAT	ANHYDRATE
BAZJAO	ANHYDRATE
BAZLUJ	SALT HYDRATE
BAZYAC	ANHYDRATE
BBEZAM	ANHYDRATE
BBZFRO	ANHYDRATE
BCHBZP	ANHYDRATE
BCOCAN	ANHYDRATE
BDOTHY	ANHYDRATE
BDTOLE	ANHYDRATE
BEBCIU	ANHYDRATE
BEBMAX	ANHYDRATE
BECDAO	ANHYDRATE
BECKUR	SOLVATE
BECMED	ANHYDRATE
BECMUT	ANHYDRATE
BEDMIG	ANHYDRATE
BEDRIL	ANHYDRATE
BEGLAA	ANHYDRATE
BEHZOF	ANHYDRATE
BEKMEK	ANHYDRATE
BELBAV	ANHYDRATE
BELBUP	ANHYDRATE
BELLIO	SALT HYDRATE
BEMLOU	ANHYDRATE

NBZANO	ANHYDRATE
NBZOAC	ANHYDRATE
NDOCLH	ANHYDRATE
NEBGUX	ANHYDRATE
NECYAX	SALT HYDRATE
NEDMUF	ANHYDRATE
NEDNOZ	ANHYDRATE
NEDZEB	SOLVATE
NEFCEG	SALT COCRYSTAL
NEFQEW	ANHYDRATE
NEGCUY	ANHYDRATE
NEHJER	COCRYSTAL
NEHREZ	ANHYDRATE
NEJHIU	SALT
NEKFIT	SOLVATE
NEMLOG	ANHYDRATE
NEMYIO	ANHYDRATE
NENFIX	ANHYDRATE
NENJOF	ANHYDRATE
NENNUP	ANHYDRATE
NEPTAZ	ANHYDRATE
NEQHAT	SALT
NEQHEX	SALT
NETCUM	ANHYDRATE
NETKOM	SOLVATE
NEWYET	ANHYDRATE
NIBGOU	ANHYDRATE
NIBVUQ	SALT
NICOAM	ANHYDRATE
NIFGAM	ANHYDRATE
NIFHIT	ANHYDRATE
NIFMAS	ANHYDRATE
NIGWEH	ANHYDRATE
NIJHUJ	ANHYDRATE
NIKSUV	SALT
NILLEB	ANHYDRATE

BEMWEV	ANHYDRATE
BENCLN	ANHYDRATE
BENNIT	ANHYDRATE
BENPRL	ANHYDRATE
BENZEN	ANHYDRATE
BENZID	SALT
BENZIE	ANHYDRATE
BENZIL	ANHYDRATE
BEQXAW	SALT HYDRATE
BERCAD	ANHYDRATE
BERPAP	ANHYDRATE
BERPIY	SALT
BERTOH	ANHYDRATE
BESKAL	ANHYDRATE
BETANC	SALT
BETJAL	ANHYDRATE
BETPIZ	ANHYDRATE
BETUTE	SALT
BEWKUJ	ANHYDRATE
BEWNAT	SALT
BEWYUY	ANHYDRATE
BEXTAB	ANHYDRATE
BEYZIO	ANHYDRATE
BEZQAZ	ANHYDRATE
BIBKUS	ANHYDRATE
BIBZUJ	ANHYDRATE
BICCIZ	ANHYDRATE
BICQUB	COCRYSTAL HYDRATE
BIDLOP	ANHYDRATE
BIGTIU	ANHYDRATE
BIHXIA	ANHYDRATE
BIJDON	HYDRATE
BILCOO	ANHYDRATE
BILCUW	ANHYDRATE
BILNAN	ANHYDRATE

NILWIO	ANHYDRATE
NIMFOE	ANHYDRATE
NIMNED	SALT
NIMRIK	ANHYDRATE
NIPSOV	ANHYDRATE
NIQZET	HYDRATE
NIRDUO	ANHYDRATE
NIRJII	ANHYDRATE
NIRTAJ*	ANHYDRATE
NISLOQ	COCRYSTAL
NISNAF	SALT HYDRATE
NITPOL	ANHYDRATE
NITRIR	COCRYSTAL
NIVPAJ	ANHYDRATE
NIVTAP	SALT
NIWFEE	ANHYDRATE
NIWJUA	SALT COCRYSTAL
NIWMEN	COCRYSTAL
NIXBEB	SOLVATE
NIYBUU	ANHYDRATE
NIYDUU	ANHYDRATE
NIZVAU	ANHYDRATE
NMACEP	SALT
NMBYAN	ANHYDRATE
NMHCOU	SOLVATE
NMZNON	ANHYDRATE
NOBHES	ANHYDRATE
NOCZUB	ANHYDRATE
NOETNA	ANHYDRATE
NOGUNA	ANHYDRATE
NOJHEZ	ANHYDRATE
NOLFUP	ANHYDRATE
NONFOM	ANHYDRATE
NONJAD	ANHYDRATE
NOPHKN	ANHYDRATE

BILQEU	ANHYDRATE
BIMCEG	ANHYDRATE
BIMYAX	SALT
BINMEQ*	ANHYDRATE
BIPCOS	ANHYDRATE
BIPDEJ	ANHYDRATE
BIPHME	ANHYDRATE
BIPTEI	ANHYDRATE
BIRFEN	ANHYDRATE
BIRKIW	ANHYDRATE
BIRMEU	HYDRATE
BISMEV	ANHYDRATE
BIVLAT	ANHYDRATE
BIVRAA	ANHYDRATE
BIVSIJ	COCRYSTAL
BIXGIY	ANHYDRATE
BIXKIE	ANHYDRATE
BIYBOA	ANHYDRATE
BIYSEH	ANHYDRATE
BIZLOM	ANHYDRATE
BIZPUW	SALT HYDRATE
BOBVIY	SALT
BOCKEK	SALT
BOCMOV	ANHYDRATE
BOCPRO	ANHYDRATE
BOHZOO	ANHYDRATE
BOKBUA	SALT
BOKCEJ	ANHYDRATE
BOKCIP	SALT
BOKVOM	ANHYDRATE
BOLCUA	ANHYDRATE
BOLMIZ	ANHYDRATE
BOLNAS	ANHYDRATE
BOLNOG	ANHYDRATE
BOMDUC	ANHYDRATE

NORFUX	ANHYDRATE
NORVEX	ANHYDRATE
NOSWAT	ANHYDRATE
NOTEST	ANHYDRATE
NOVNIX	ANHYDRATE
NOVQOG	SALT
NTBZAM	ANHYDRATE
NUDDIZ	SOLVATE
NUDGEZ	ANHYDRATE
NUGKIL	ANHYDRATE
NUGZEV	COCRYSTAL
NUHZEX	ANHYDRATE
NUJGUV	ANHYDRATE
NUJKEJ	ANHYDRATE
NUJLIN	ANHYDRATE
NUJPIT	ANHYDRATE
NUJTUI	ANHYDRATE
NUKMAH	ANHYDRATE
NUKPAK	SALT
NUKVOF	SALT
NUKWEW	COCRYSTAL
NUKXEX	COCRYSTAL
NUKXUN	COCRYSTAL
NUKYUO	COCRYSTAL
NULTIY	ANHYDRATE
NUNHUZ	ANHYDRATE
NUNSIY	SALT
NUPCOS	ANHYDRATE
NUQXAA	ANHYDRATE
NUQXEC	ANHYDRATE
NURAMH	HYDRATE
NURFAH	ANHYDRATE
NURJEP	HYDRATE SOLVATE
NUVMAS	ANHYDRATE
NUWTOP	ANHYDRATE

BOPJIB	ANHYDRATE
BOPKOG	SALT
BOPNID	ANHYDRATE
BOPQAY	HYDRATE
BOPSAA	ANHYDRATE
BOPSEF	ANHYDRATE
BOQCUF	ANHYDRATE
BOQQUT	ANHYDRATE
BORCUG	ANHYDRATE
BORSEG	ANHYDRATE
BORVAG	ANHYDRATE
BOVCEV	ANHYDRATE
BOVWIT	SALT
BOVXOB	COCRYSTAL
BOWJII	COCRYSTAL
BOWWOZ	ANHYDRATE
BOZFIF	ANHYDRATE
BPHENO	ANHYDRATE
BPROMA	ANHYDRATE
BRACTN*	ANHYDRATE
BRESTO	ANHYDRATE
BRMACA	ANHYDRATE
BRMANC	ANHYDRATE
BROFRM	ANHYDRATE
BTUPTE	ANHYDRATE
BUDKES	SALT
BUDREZ	ANHYDRATE
BUDYOP	ANHYDRATE
BUFTED	ANHYDRATE
BUHNAV	SALT
BUJBEN	ANHYDRATE
BUNKOK	ANHYDRATE
BUNPEF	SALT COCRYSTAL
BUPLIH	SALT
BUQQAF	COCRYSTAL HYDRATE

NUXFAP	ANHYDRATE
NUXJUM	ANHYDRATE
NUYCEQ	SALT
NUYDER	SALT
NUYJEW	ANHYDRATE
NXENAM	ANHYDRATE
OBAZOI*	SOLVATE
OBEQAN	HYDRATE
OBIDOS	ANHYDRATE
OBOZIQ	ANHYDRATE
OBUPUY	ANHYDRATE
OBUZUI	ANHYDRATE
OCAKEJ	SALT
OCALAG	ANHYDRATE
OCHTET	ANHYDRATE
OCIMUJ	SALT HYDRATE
OCIYEF	SOLVATE
OCMETD	ANHYDRATE
OCRSOL	ANHYDRATE
OCUJOM	ANHYDRATE
OCUSUA	ANHYDRATE
ODAJIM	ANHYDRATE
ODASAO	ANHYDRATE
ODEFOT	ANHYDRATE
ODEGIN	ANHYDRATE
ODIGOZ	ANHYDRATE
ODOBIT	COCRYSTAL
OFEJUG	SALT
OFEXAY	ANHYDRATE
OFIQUR	ANHYDRATE
OFNAPH	ANHYDRATE
OFOGAR	ANHYDRATE
OFUFIG	ANHYDRATE
OFUVUG	SOLVATE
OGABEF	ANHYDRATE

BURLAC	ANHYDRATE
BURTIT	ANHYDRATE
BURYOC	ANHYDRATE
BUTPOV	ANHYDRATE
BUTSIA	ANHYDRATE
BUVKEJ	ANHYDRATE
BUVZUN	SALT
BUYJAH	ANHYDRATE
BUYZUQ	ANHYDRATE
BXLAZC	ANHYDRATE
BXZCPN	SALT
BZAMID	ANHYDRATE
BZANIL*	ANHYDRATE
BZCHOL	ANHYDRATE
BZDMAZ	ANHYDRATE
BZINDA	ANHYDRATE
BZTRAZ	ANHYDRATE
BZTZAD	ANHYDRATE
BZYACO	ANHYDRATE
CABTUV	ANHYDRATE
CABWIM	ANHYDRATE
CABZOU	SALT SOLVATE
CACBAK	ANHYDRATE
CACDAM	ANHYDRATE
CADYLA	ANHYDRATE
CAFORM	SALT
CAGMAY	SALT
CAHKAX	SALT
CAHSEK	SOLVATE
CAKRUB	ANHYDRATE
CALCLA	SALT HYDRATE
CALOXM	SALT HYDRATE
CALQOW	SALT
CAMALD	SALT HYDRATE
CAMPQU	ANHYDRATE
CANDUR	ANHYDRATE

OGAPET	ANHYDRATE
OGELUI	ANHYDRATE
OGEPUM	ANHYDRATE
OGILIA	ANHYDRATE
OGISON	HYDRATE
OHADAD	ANHYDRATE
OHIBOW	ANHYDRATE
OHIJAR	ANHYDRATE
OHOVAJ	SALT
OLATUR	ANHYDRATE
OLEBAJ	SALT
OLECAC	ANHYDRATE
OLENAN	ANHYDRATE
OLEYEK	ANHYDRATE
OLUPAM	SALT
OMAYUX	ANHYDRATE
OMEFAN	ANHYDRATE
OMEKAS	SALT
OMIYEO	ANHYDRATE
OMOPAH	ANHYDRATE
OMUZOM	SALT
ONAVEF	ANHYDRATE
ONITAN	ANHYDRATE
ONOXUL	ANHYDRATE
OPIJAZ	HYDRATE
OPITOX	SALT
OPOXAZ	ANHYDRATE
OPSIOX	ANHYDRATE
OQUPUM	ANHYDRATE
ORABEP	ANHYDRATE
OSAGAR	ANHYDRATE
OTIYUM*	COCRYSTAL
OTIZAT	COCRYSTAL
OTUCOW	SOLVATE
OTUDAJ	SOLVATE
OTUDIR	SOLVATE

CAPLEK	ANHYDRATE
CAQYAW	ANHYDRATE
CAQYIE	ANHYDRATE
CARBTC	ANHYDRATE
CARWID	ANHYDRATE
CARZIF	ANHYDRATE
CASHOT	ANHYDRATE
CASRPP	SALT
CATDIM	ANHYDRATE
CAVCUY	SALT
CAVJAM	ANHYDRATE
CAVJIU	SALT SOLVATE
CAVVUQ	SALT
CAWDUZ	ANHYDRATE
CAXHIS	ANHYDRATE
CAXMOD	ANHYDRATE
CAZFUE	ANHYDRATE
CAZLAR	COCRYSTAL
CAZTBZ	COCRYSTAL
CBENPH	ANHYDRATE
CBFBZF	ANHYDRATE
CBMZPN	ANHYDRATE
CBZCAN	ANHYDRATE
CEBGOF	ANHYDRATE
CEBGUL	ANHYDRATE
CEBKEZ	ANHYDRATE
CEDGAV	ANHYDRATE
CEHQEM	ANHYDRATE
CEKJAD	ANHYDRATE
CEKKOU	COCRYSTAL
CELBEA	ANHYDRATE
CELRAO	SOLVATE
CELWIB	SALT
CEMCED	SALT
CEMDON	ANHYDRATE
CEMQOA	ANHYDRATE

OVAYAM	ANHYDRATE
OVAYIU	ANHYDRATE
OWEMIN	SALT
OXACDH-D	HYDRATE
OXADOH	SALT
OXALAC	ANHYDRATE
OXYTET	ANHYDRATE
OYEWUL	ANHYDRATE
PABHAB	ANHYDRATE
PABZAM	ANHYDRATE
PACBOK	ANHYDRATE
PACFOO	ANHYDRATE
PACGAB	ANHYDRATE
PAHDEI	ANHYDRATE
PAHRIZ	ANHYDRATE
PAJCOT	ANHYDRATE
PAJREY	ANHYDRATE
PAKPUO	ANHYDRATE
PAKQUP	ANHYDRATE
PAMTAY	ANHYDRATE
PAMVIK	ANHYDRATE
PANQUO	ANHYDRATE
PANQUS	COCRYSTAL
PAQVUA	ANHYDRATE
PARQUI	SALT
PARSON	ANHYDRATE
PASXIS	SALT HYDRATE
PATSEJ	ANHYDRATE
PATSIM	ANHYDRATE
PATVEM	SALT
PAVBEU	ANHYDRATE
PAXWEQ	ANHYDRATE
PAZCBN	ANHYDRATE
PAZPTE	ANHYDRATE
PBBTAZ	ANHYDRATE
PBOXAT	ANHYDRATE

CENGEH	ANHYDRATE
CENNOA	SALT SOLVATE
CENRIW	ANHYDRATE
CEPXHP	ANHYDRATE
CERBEG	ANHYDRATE
CERJAM	ANHYDRATE
CERLOA	ANHYDRATE
CERYAA	ANHYDRATE
CETRUQ	ANHYDRATE
CEVPOK	COCRYSTAL
CEVWAC	ANHYDRATE
CEWGAM	ANHYDRATE
CEWVOP	ANHYDRATE
CEXDAK	ANHYDRATE
CEZDOC	ANHYDRATE
CHAPEP	SALT
CHIPTH	ANHYDRATE
CHLSAN	ANHYDRATE
CHOLEC*	ANHYDRATE
CHOLEU	SOLVATE
CHOLSI	ANHYDRATE
CHXAMH	HYDRATE
CIBZAQ	SALT
CICYES	ANHYDRATE
CICYOD	ANHYDRATE
CIDFEC	ANHYDRATE
CIDXUI	ANHYDRATE
CIFDOK	SALT
CIFLEI	ANHYDRATE
CIGHEG	SALT
CIGKAF	ANHYDRATE
CIKDIK	ANHYDRATE
CILHIO	SALT
CILJIQ	ANHYDRATE
CIMETD	ANHYDRATE
CIMMUI	ANHYDRATE

PBTCHT	ANHYDRATE
PBUPEA	SALT
PCBZAM	ANHYDRATE
PCHSAN	ANHYDRATE
PCLPYR*	ANHYDRATE
PCPZOL	ANHYDRATE
PDABZA	ANHYDRATE
PDHTEZ	ANHYDRATE
PDSILZ	ANHYDRATE
PDTOMS	COCRYSTAL
PEAMAN	SALT
PECNOC	ANHYDRATE
PEDTAV	ANHYDRATE
PEDTUP	ANHYDRATE
PEDWOM	ANHYDRATE
PEFBAD	ANHYDRATE
PEFGEO	COCRYSTAL
PEFGIR	ANHYDRATE
PEFHOZ	SOLVATE
PEFJER	ANHYDRATE
PEFKAO	ANHYDRATE
PEFKOC	ANHYDRATE
PEFKUI	ANHYDRATE
PEFLET	ANHYDRATE
PEFMET	ANHYDRATE
PEFMEU	SALT
PEFNIZ	COCRYSTAL
PEFTIE	ANHYDRATE
PEGVAY	ANHYDRATE
PEGWII	ANHYDRATE
PEHZUX	ANHYDRATE
PEJBUC	ANHYDRATE
PEJCAJ	ANHYDRATE
PEJHOD	ANHYDRATE
PEJJOF	ANHYDRATE
PEKGER	ANHYDRATE

CINCUY	SALT
CINMAC	ANHYDRATE
CINMER	ANHYDRATE
CINYEE	ANHYDRATE
CIPJAM	SOLVATE
CIPPAT	ANHYDRATE
CIPYAB	SALT
CIRTEC	COCRYSTAL HYDRATE
CISHOC	SALT
CISJEV	SALT
CISQAW	SALT SOLVATE
CITRIG	ANHYDRATE
CIVTUW	ANHYDRATE
CIWJIB	SOLVATE
CIWMUS	ANHYDRATE
CIWVAH	ANHYDRATE
CIXCES	ANHYDRATE
CIXKEA	ANHYDRATE
CIYFIB	SALT COCRYSTAL HYDRATE
CIYRIL	ANHYDRATE
CIYXAK	ANHYDRATE
CIZDUK	ANHYDRATE
CLACAM	ANHYDRATE
CLACET	ANHYDRATE
CLBZAM	ANHYDRATE
CLBZNT	ANHYDRATE
CLFORM	ANHYDRATE
CLPHOL	ANHYDRATE
CLPHTE	ANHYDRATE
CLPXAD	ANHYDRATE
CLURAC	ANHYDRATE
CMPIXZ	ANHYDRATE
CNITBZ	ANHYDRATE
COBBIE	ANHYDRATE
COBKIN	ANHYDRATE

PEKJEW	ANHYDRATE
PEKJUM	HYDRATE
PEMZAI	SOLVATE
PEMZAJ	ANHYDRATE
PENCEN	ANHYDRATE
PEPHUN	ANHYDRATE
PEPQEG	SALT HYDRATE
PERLEN	ANHYDRATE
PERPIL	SOLVATE
PERYTN	ANHYDRATE
PETLAD	ANHYDRATE
PETNER	ANHYDRATE
PEVQUD	SALT
PEWXAQ	ANHYDRATE
PEXKOS	SALT
PEXTAO	SOLVATE
PEXVOE	SALT HYDRATE
PEYMIQ	ANHYDRATE
PEZBOL	ANHYDRATE
PEZLOV	SALT
PFLCPC	SALT HYDRATE
PGTAPA	ANHYDRATE
PHBARB	ANHYDRATE
PHENAN	ANHYDRATE
PHENAZ	ANHYDRATE
PHENOL	ANHYDRATE
PHISCY	ANHYDRATE
PHNSNB	COCRYSTAL
PHOGLY	ANHYDRATE
PHOSBZ	ANHYDRATE
PHPLAC	ANHYDRATE
PHTGLY	ANHYDRATE
PHTHCY	ANHYDRATE
PHTRPL	ANHYDRATE
PIBDIP	SOLVATE

COBWOF	ANHYDRATE
COCHEJ	ANHYDRATE
COHQUN	SOLVATE
COHWIF	COCRYSTAL
COKBIN	ANHYDRATE
COLDOX	ANHYDRATE
COLTAY	ANHYDRATE
COMPEA	ANHYDRATE
COMXAD	SALT
COPTUW	ANHYDRATE
COQJOH	HYDRATE
COQWOU	HYDRATE
CORDCP	ANHYDRATE
CORDIW	ANHYDRATE
COSDAR	ANHYDRATE
COTSAF	ANHYDRATE
COTYEP	ANHYDRATE
COWRIP	ANHYDRATE
COXDHN	ANHYDRATE
COYLAF	ANHYDRATE
COYMOS	ANHYDRATE
CPAHYD	ANHYDRATE
CPHAZO	SOLVATE
CRESOL	ANHYDRATE
CRETAL	ANHYDRATE
CSIXPH	ANHYDRATE
CTMTNA	ANHYDRATE
CTPHMI	ANHYDRATE
CTVHVH	ANHYDRATE
CUBHUE	ANHYDRATE
CUCKOA	ANHYDRATE
CUCSEY	SALT
CUFKOE	SALT
CUGJAP	SALT SOLVATE
CUHTOO	SALT
CUKCAM	ANHYDRATE

PICAMD	ANHYDRATE
PIDFEN	ANHYDRATE
PIFLAR	ANHYDRATE
PIFZAG	ANHYDRATE
PIHLOJ	ANHYDRATE
PIHSEF	SALT HYDRATE
PIHSUV	ANHYDRATE
PIJHIA	COCRYSTAL
PIMBAP	ANHYDRATE
PIMELA	ANHYDRATE
PIMTAZ	ANHYDRATE
PINCOL	ANHYDRATE
PINPAD	ANHYDRATE
PIPGIG	SALT
PIPINE	ANHYDRATE
PIPPTC	SALT
PIQLIK	ANHYDRATE
PIRNOV	COCRYSTAL
PIRQIS	COCRYSTAL
PIWBUT	SOLVATE
PIWDUV	ANHYDRATE
PIWKOV	SOLVATE
PIWPER	ANHYDRATE
PIYQEU	COCRYSTAL
PMAANO	ANHYDRATE
PMZPCY	ANHYDRATE
PNEOSI	ANHYDRATE
POBENZ	ANHYDRATE
POBPIG	ANHYDRATE
POCPUS	HYDRATE
PODQIJ	ANHYDRATE
POFHUN	ANHYDRATE
POFLAX	ANHYDRATE
POFRAE	ANHYDRATE
POGVAJ	ANHYDRATE
POHJON	ANHYDRATE

CUKCIU	ANHYDRATE
CUKHUM	ANHYDRATE
CUKJEY	ANHYDRATE
CUMMIG	ANHYDRATE
CUMTAF	ANHYDRATE
CUNCOF	ANHYDRATE
CUNKAY	ANHYDRATE
CUNKUS	ANHYDRATE
CUNPUW	ANHYDRATE
CUPQUB	ANHYDRATE
CUPREM	ANHYDRATE
CUQZAP	SALT
CURJEE	SALT
CUVSIV	ANHYDRATE
CUYQUI	ANHYDRATE
CYACAC	ANHYDRATE
CYANAC	ANHYDRATE
CYCHEX	ANHYDRATE
CYTIAC	ANHYDRATE
CYTSIN*	ANHYDRATE
CYVTPA	ANHYDRATE
DACMOR	ANHYDRATE
DADDER	ANHYDRATE
DADTEH	ANHYDRATE
DAFBIV	ANHYDRATE
DAGWUF	ANHYDRATE
DAGYIV	SOLVATE
DAHMII	ANHYDRATE
DAHPIL	ANHYDRATE
DAJKIK	ANHYDRATE
DAJVUG	ANHYDRATE
DAJZAQ	SOLVATE
DAKFUQ	ANHYDRATE
DAKPUA	SALT SOLVATE
DAKXUI	ANHYDRATE
DAMPEO	ANHYDRATE

POKLUY	SALT
POMDAW	ANHYDRATE
POMLIO	ANHYDRATE
POPFAC	ANHYDRATE
POPGUX	ANHYDRATE
POPJEL	ANHYDRATE
POPJUA	ANHYDRATE
POPLIR	ANHYDRATE
POQGOS	SOLVATE
PORREU	ANHYDRATE
POSCUV	SALT SOLVATE
POSTEW	SALT
POVSEY*	ANHYDRATE
POXHYN	ANHYDRATE
POYGIT	ANHYDRATE
PPDACS	ANHYDRATE
PROGST	ANHYDRATE
PRONAC	ANHYDRATE
PTCDEC	ANHYDRATE
PTRPHE	ANHYDRATE
PTZTCQ	COCRYSTAL
PUBMUU	ANHYDRATE
PUBYAM	ANHYDRATE
PUCMIJ	ANHYDRATE
PUDXES	ANHYDRATE
PUDYOC	SALT
PUFSIT	ANHYDRATE
PUGVUK	ANHYDRATE
PUGWUK	ANHYDRATE
PUKMAK	SALT
PUKVEX	SALT
PULCOP	ANHYDRATE
PULNUF	ANHYDRATE
PUMRUK	SALT
PUMVIC	ANHYDRATE
PUMZII	ANHYDRATE

DANGAL	ANHYDRATE
DANQEP	ANHYDRATE
DAQDII	ANHYDRATE
DARWAU	ANHYDRATE
DASPAO	ANHYDRATE
DATDIL	ANHYDRATE
DATWUS	ANHYDRATE
DATZAA	ANHYDRATE
DAVLAP	ANHYDRATE
DAVQAS	ANHYDRATE
DAVVUR	ANHYDRATE
DAVWAY	ANHYDRATE
DAWBEI	ANHYDRATE
DAWFUE	ANHYDRATE
DAWGAL	ANHYDRATE
DAWHOY	ANHYDRATE
DAWMAP	ANHYDRATE
DAXSOM	ANHYDRATE
DAXTIH	ANHYDRATE
DAXYUX	ANHYDRATE
DAYWAC*	COCRYSTAL
DAZABZ	ANHYDRATE
DAZBAJ	ANHYDRATE
DBEZLM	ANHYDRATE
DBMBPO	ANHYDRATE
DBNTHR	ANHYDRATE
DBPHEN	ANHYDRATE
DBXZCP	ANHYDRATE
DBZSEL	ANHYDRATE
DCBFRO	ANHYDRATE
DCDHNQ	ANHYDRATE
DCHEXC	ANHYDRATE
DCHXCS	ANHYDRATE
DCLANT	ANHYDRATE
DCLBEN	ANHYDRATE
DCLBQN	ANHYDRATE

PUNBAD	ANHYDRATE
PUNVIE	ANHYDRATE
PUPBAD	ANHYDRATE
PUQGAL	SALT HYDRATE
PUQYOQ	ANHYDRATE
PUQZIM	ANHYDRATE
PURCEM	ANHYDRATE
PURFIT	ANHYDRATE
PURSEB	ANHYDRATE
PUVQIG	HYDRATE
PUVVUX	ANHYDRATE
PUXLUP	ANHYDRATE
PUZGAT	HYDRATE
PYAZAC	ANHYDRATE
PYDCLI	SALT
PYDSYD	ANHYDRATE
PYMSUL	ANHYDRATE
PYPTCQ	SALT
PYRDNA	ANHYDRATE
PYRDNO-D	ANHYDRATE
PYRENE	ANHYDRATE
PYRHCL	SALT
PYRIDO	ANHYDRATE
PYRPIC	SALT
PYRTCQ	COCRYSTAL
PYRZIN	ANHYDRATE
PYRZOL	ANHYDRATE
PYZPYT	ANHYDRATE
QAHGAH	ANHYDRATE
QAJQAV	ANHYDRATE
QAJTUQ	ANHYDRATE
QAKREB	ANHYDRATE
QAKZUY	ANHYDRATE
QALHAN	ANHYDRATE
QALPEA	HYDRATE
QAMNAT	ANHYDRATE

DCLNAP	ANHYDRATE
DEBMOM	SALT
DECFDP	ANHYDRATE
DECYIU	ANHYDRATE
DEFDUN	ANHYDRATE
DEFQIP	ANHYDRATE
DEFRUB	HYDRATE
DEGGEB	SALT
DEHREN	ANHYDRATE
DEKDAY	SALT
DEKDIG	ANHYDRATE
DEKYEX	ANHYDRATE
DEMQIW	ANHYDRATE
DEMYEZ	SALT
DENWUP	SALT
DEPOAM	ANHYDRATE
DERBEH	ANHYDRATE
DERRAT	ANHYDRATE
DESKER	ANHYDRATE
DETBAA	ANHYDRATE
DETBIO	ANHYDRATE
DETBUZ	ANHYDRATE
DETSUQ	ANHYDRATE
DETZUX	ANHYDRATE
DEVSAZ	SOLVATE
DEWVOQ	ANHYDRATE
DEXBUF	SALT
DEXVEH	ANHYDRATE
DEZJIB	SALT
DEZRAD	ANHYDRATE
DEZZAL	SALT
DGLYCN	SALT
DHANQU	ANHYDRATE
DHNAPH	ANHYDRATE
DHXANT	ANHYDRATE
DHYANT	ANHYDRATE

QAMQEC	ANHYDRATE
QAMWEH	ANHYDRATE
QAMXUY	ANHYDRATE
QANHAP	ANHYDRATE
QANTUV	ANHYDRATE
QAPZEN	ANHYDRATE
QARXOY	ANHYDRATE
QATCIX	ANHYDRATE
QATDIZ	SALT
QAXMEH	ANHYDRATE
QAXPEM	ANHYDRATE
QAXSUD	ANHYDRATE
QEGBUB	ANHYDRATE
QEGWEG	ANHYDRATE
QEJZAH	ANHYDRATE
QEKBEN	ANHYDRATE
QEKDIT	ANHYDRATE
QERJAZ	ANHYDRATE
QERWAM	ANHYDRATE
QESJAA	ANHYDRATE
QESJEE	ANHYDRATE
QESJII	ANHYDRATE
QESJOO	ANHYDRATE
QESZUK	ANHYDRATE
QEVXIY	SALT
QEWJUZ	ANHYDRATE
QEXBEB	SALT
QICGEP	ANHYDRATE
QICGIT	SOLVATE
QIDVII	ANHYDRATE
QIFDIT	ANHYDRATE
QIFJAS	ANHYDRATE
QIFKEX	ANHYDRATE
QIGBEN	ANHYDRATE
QIJTOS	ANHYDRATE
QIJZOY	ANHYDRATE

DIBDEY	ANHYDRATE
DIBTEP	ANHYDRATE
DIBZAQ	SALT HYDRATE
DICCEX	SALT
DICGED	ANHYDRATE
DICNIM	ANHYDRATE
DICNUY	ANHYDRATE
DIDMTE	ANHYDRATE
DIFVET	ANHYDRATE
DIJYOL	ANHYDRATE
DIKHAH	ANHYDRATE
DIKTAT	ANHYDRATE
DIKVID	ANHYDRATE
DILFEL	ANHYDRATE
DILVIF	SALT HYDRATE
DIMETH	ANHYDRATE
DINPAR	COCRYSTAL
DINRUP	COCRYSTAL
DINSOK	COCRYSTAL
DIPGIS	ANHYDRATE
DIPHUH	COCRYSTAL
DIPLAR	ANHYDRATE
DIPQAU	ANHYDRATE
DISQIG	ANHYDRATE
DITBEN	ANHYDRATE
DIUREA	ANHYDRATE
DIVHOF	SALT
DIWBAM	ANHYDRATE
DIWGAS	ANHYDRATE
DIWKOK	ANHYDRATE
DIWMOM	ANHYDRATE
DIWVEM	ANHYDRATE
DIXBIW	ANHYDRATE
DIXTEN	ANHYDRATE
DIYJUQ	SALT HYDRATE
DIYLIG	ANHYDRATE

QIKFEV	ANHYDRATE
QIKFIZ	ANHYDRATE
QIKNUT	ANHYDRATE
QIKZAN	ANHYDRATE
QIMCAS	ANHYDRATE
QIMGAU	ANHYDRATE
QIMKIG	ANHYDRATE
QIMKOM	HYDRATE
QIMXOB	SALT HYDRATE
QIMZOB	ANHYDRATE
QIQLIL	SALT
QIRGIJ	ANHYDRATE
QIVBAA*	COCRYSTAL
QIXFUY	ANHYDRATE
QIXZAY	ANHYDRATE
QIYFEJ	ANHYDRATE
QIYKAL	SALT
QIYKEQ	ANHYDRATE
QIZHIQ	ANHYDRATE
QNACRD	ANHYDRATE
QNGHSU	ANHYDRATE
QOCNAX	ANHYDRATE
QOCNIF*	ANHYDRATE
QODGUM	SALT
QODQUV	ANHYDRATE
QODTAE	ANHYDRATE
QODTIO	ANHYDRATE
QODZUG	ANHYDRATE
QOGNEF	ANHYDRATE
QOGREJ	ANHYDRATE
QOHBEV	ANHYDRATE
QOHSAJ	ANHYDRATE
QOHYAO	HYDRATE
QOPSEU	ANHYDRATE
QOQVOH	SALT
QOQYEC	ANHYDRATE

DLABUT	ANHYDRATE
DLMALC	ANHYDRATE
DLMAND	ANHYDRATE
DLMETA	ANHYDRATE
DLMSUC	ANHYDRATE
DLNLUA	ANHYDRATE
DMADEN	ANHYDRATE
DMAFBZ	SOLVATE
DMANTL	ANHYDRATE
DMBOPN	ANHYDRATE
DMBZAC	ANHYDRATE
DMETSO	ANHYDRATE
DMFUSC	ANHYDRATE
DMMTCN	SALT
DMNPYO	ANHYDRATE
DMOXBA	ANHYDRATE
DMURAC	ANHYDRATE
DOBTOD	SALT
DOBTUJ	SALT
DOCCEF	ANHYDRATE
DODBIJ	ANHYDRATE
DODDAB	ANHYDRATE
DODRIZ	ANHYDRATE
DOFNAN	SALT
DOFSUM	ANHYDRATE
DOGWOL	ANHYDRATE
DOHBOC	ANHYDRATE
DOHREX	ANHYDRATE
DOHSEA	ANHYDRATE
DOJFAJ	ANHYDRATE
DOJHAN	ANHYDRATE
DOKGUG	COCRYSTAL
DOKNOI	ANHYDRATE
DOLBIR	ANHYDRATE
DOLJET	ANHYDRATE
DOLNEZ	SALT

QOTGOV	ANHYDRATE
QOTHEM	SALT SOLVATE
QOTMUI	ANHYDRATE
QOVREZ	ANHYDRATE
QOYGUH	ANHYDRATE
QOYJOD	ANHYDRATE
QOYNOH	ANHYDRATE
QQQAUG	ANHYDRATE
QQQAUJ	ANHYDRATE
QQQAXG	ANHYDRATE
QQQAXJ	ANHYDRATE
QQQAZJ	SALT
QQQBNG	ANHYDRATE
QQQCIG	ANHYDRATE
QQQCIV	ANHYDRATE
QQQCYA	SALT HYDRATE
QQQDDS	ANHYDRATE
QQQDQY	SALT
QQQDVM	ANHYDRATE
QQQEJV-D	ANHYDRATE
QQQFDJ	ANHYDRATE
QQQGMA	SALT
QQQGVV	SOLVATE
QUBPAF	ANHYDRATE
QUBPIN	ANHYDRATE
QUCWAM	SOLVATE
QUFDUQ	SALT SOLVATE
QUFFAY	SALT SOLVATE
QUIDON	COCRYSTAL
QUJDOP	SOLVATE
QUKJIR	ANHYDRATE
QULLUF	COCRYSTAL
QUMFEJ	SOLVATE
QUNDIO	ANHYDRATE
QUPHEN	ANHYDRATE
QURWOQ	SALT

DONTIJ	ANHYDRATE
DOPDAN	ANHYDRATE
DOPLOL	ANHYDRATE
DOPPAB	ANHYDRATE
DOPPOP	ANHYDRATE
DOQNAY	ANHYDRATE
DOQWEN	ANHYDRATE
DOVGEC	ANHYDRATE
DOVJOP	ANHYDRATE
DOVJUT	ANHYDRATE
DOVVIT	SALT
DOXANN	ANHYDRATE
DOZJAD	ANHYDRATE
DOZYAS	ANHYDRATE
DPBCOC	ANHYDRATE
DPENAM	ANHYDRATE
DPGUAN	ANHYDRATE
DPHACT	ANHYDRATE
DPHETH	ANHYDRATE
DPHSAM	ANHYDRATE
DPIPDS	ANHYDRATE
DPOCRS	ANHYDRATE
DPPETH	ANHYDRATE
DPPSEM	ANHYDRATE
DPSIFL	ANHYDRATE
DPYRAM	ANHYDRATE
DTBPTR	ANHYDRATE
DTCTCQ	SALT
DTTDDO	ANHYDRATE
DTTROP	SALT
DUCKOB-D	ANHYDRATE
DUCLUJ	SALT
DUDCUC	ANHYDRATE
DUDNAS	ANHYDRATE
DUDZIL	ANHYDRATE
DUHBAK	SOLVATE

QUSQEB	SOLVATE
QUYLEC	SALT
RACLIS	ANHYDRATE
RADLAK	ANHYDRATE
RAFFAF	ANHYDRATE
RAFFIO	ANHYDRATE
RAGLIV	ANHYDRATE
RAHSUO	SALT
RAKCAJ	SALT HYDRATE
RAKMEX	ANHYDRATE
RAMKIZ	ANHYDRATE
RAMNUP	ANHYDRATE
RAPTEI	ANHYDRATE
RAQDIW	SALT
RAQTIN	SALT
RARXEP	ANHYDRATE
RASTOU	HYDRATE
RASXIS	ANHYDRATE
RATVIR	ANHYDRATE
RAVMUX	SALT HYDRATE
RAXRAK	ANHYDRATE
RAYCID	ANHYDRATE
RBTCNQ	SALT
REBGAG	ANHYDRATE
REBYEE	SALT HYDRATE
RECQOH	ANHYDRATE
REHGUI	ANHYDRATE
REHLAT	ANHYDRATE
REKBUE	ANHYDRATE
RENSEJ	SALT
REPFOH	ANHYDRATE
REPWUG	ANHYDRATE
REPXER	ANHYDRATE
RESZUK	ANHYDRATE
REVCEB	SALT HYDRATE
REVCIF	SALT HYDRATE

DULZIT	SALT
DUMVAJ	ANHYDRATE
DUNGIC	ANHYDRATE
DUNWIT	ANHYDRATE
DUNWOZ	ANHYDRATE
DUNXAM	ANHYDRATE
DUPTOX	ANHYDRATE
DUQJOP	ANHYDRATE
DURDAV	SALT
DURGAY	SALT HYDRATE
DURZAR	COCRYSTAL
DURZUM	ANHYDRATE
DUVFUV	SOLVATE
DUVYEZ	SALT
DUVZOJ	ANHYDRATE
DUWBUT	ANHYDRATE
DUXSUK	ANHYDRATE
DUYCUW	ANHYDRATE
DUYZUT	ANHYDRATE
DXCYTD	ANHYDRATE
DXYLEN	ANHYDRATE
EABVEI	ANHYDRATE
EAPTZC	SALT
EAZOBZ	ANHYDRATE
EBEKIG	ANHYDRATE
EBIDUP	ANHYDRATE
EBIGUR	ANHYDRATE
EBIRIR	ANHYDRATE
EBIXET	ANHYDRATE
EBIZIZ	ANHYDRATE
EBIZUL	ANHYDRATE
EBURCL	ANHYDRATE
EBYBUA	ANHYDRATE
ECELON	SOLVATE
ECITIU	ANHYDRATE

REWDOM	ANHYDRATE
REWPUE	ANHYDRATE
REYDEE	SALT
REYTEU	ANHYDRATE
REZNAL	ANHYDRATE
REZRIZ	SOLVATE HYDRATE
RIDFOA	SALT
RIDJUJ	SALT
RIFBAL	ANHYDRATE
RIFMUQ	ANHYDRATE
RIFQAY	COCRYSTAL
RIGHUK	ANHYDRATE
RIGVEJ	ANHYDRATE
RIHVUZ	ANHYDRATE
RIMKAB	ANHYDRATE
RINDID	SALT
RINTEN	SALT HYDRATE
RIPBOH	ANHYDRATE
RIRZIC	ANHYDRATE
RISHIM	SALT
RISZOK	ANHYDRATE
RITBED	ANHYDRATE
RITNOY	ANHYDRATE
RIVFAE	ANHYDRATE
RIWTAT	ANHYDRATE
RIWTEX	ANHYDRATE
RIWTIB	ANHYDRATE
RIWWEA	COCRYSTAL
RIYTUP	HYDRATE
RIZQOG	SALT
ROBKEY	ANHYDRATE
RODLIG	ANHYDRATE
ROFKAZ	ANHYDRATE
ROFVAK	ANHYDRATE
ROJROZ	COCRYSTAL

ECOBUU	ANHYDRATE
EDAXEM	ANHYDRATE
EDIGUU	ANHYDRATE
EDOPUK	ANHYDRATE
EDOROG	ANHYDRATE
EDOSAT	ANHYDRATE
EDOSOH	ANHYDRATE
EFAWIT	ANHYDRATE
EFIKOU	ANHYDRATE
EFOZAB	COCRYSTAL
EFUMAU	ANHYDRATE
EFUPEB	ANHYDRATE
EFURIH	ANHYDRATE
EGOPIB	ANHYDRATE
EGUTEF	SALT
EHESAL	ANHYDRATE
EHOPAS*	ANHYDRATE
EHOWIH	ANHYDRATE
EHULAV	ANHYDRATE
EJEQAL	ANHYDRATE
EJESIV	ANHYDRATE
EJUQIK	SALT
EKECOM	COCRYSTAL
EKIGEK	SOLVATE
ELATAN	SALT
ELEGUY	COCRYSTAL
ELOHUJ*	ANHYDRATE
ELOPIE	ANHYDRATE
ELUREJ	ANHYDRATE
EMAKUY	ANHYDRATE
EMAMEL	ANHYDRATE
EMISOJ	COCRYSTAL
EMPIPP	SALT
ENAPOZ	HYDRATE SOLVATE
ENATAO	ANHYDRATE
ENAZOI	COCRYSTAL

ROKBOI	SALT
ROKCEZ	ANHYDRATE
ROKQUF	ANHYDRATE
ROLFEE	ANHYDRATE
ROLGEE	ANHYDRATE
ROPQOC	COCRYSTAL
ROPQUI	COCRYSTAL
ROZRAB	ANHYDRATE
RUCJUV	ANHYDRATE
RUFBAW	ANHYDRATE
RUFGUV	ANHYDRATE
RUFHIK*	ANHYDRATE
RUGSIW	ANHYDRATE
RUKFIO	ANHYDRATE
RULKUG	COCRYSTAL
RUNGEN	ANHYDRATE
RUPTIH	ANHYDRATE
RURROM	COCRYSTAL
RUVPII	SALT
RUVZOY	ANHYDRATE
RUWYIR	ANHYDRATE
RUXWAI	ANHYDRATE
RUYZOA	ANHYDRATE
RUZXEP	ANHYDRATE
SABCII	ANHYDRATE
SACRET	ANHYDRATE
SADVUQ	ANHYDRATE
SAFJIT	ANHYDRATE
SAFPIY	ANHYDRATE
SAGWUU	ANHYDRATE
SAGXOP	ANHYDRATE
SAGXUT	SALT
SAGYIK	ANHYDRATE
SAHZOS	ANHYDRATE
SAJGAL	ANHYDRATE
SAJMOH	ANHYDRATE

ENIZIK	ANHYDRATE
ENVFUR	ANHYDRATE
EPEJUF	SALT
EPITED	ANHYDRATE
EPOPDO	ANHYDRATE
EPUPUB	COCRYSTAL
EQUZIZ	ANHYDRATE
EQZBMI	SALT HYDRATE
ERIXAE	ANHYDRATE
EROXAL	SALT
ESALUF	ANHYDRATE
ESIVOR	ANHYDRATE
ESIWEI	ANHYDRATE
ESIWUY	ANHYDRATE
ESOFIC	ANHYDRATE
ESTRON	ANHYDRATE
ETANOL	ANHYDRATE
ETBBAR	ANHYDRATE
ETBZIM	ANHYDRATE
ETCTEP	ANHYDRATE
ETDIAM	ANHYDRATE
ETEDOX	ANHYDRATE
ETEFIT	SOLVATE
ETEXIK	ANHYDRATE
ETEYUX	ANHYDRATE
ETHCTE	ANHYDRATE
ETOCEV	ANHYDRATE
ETOJUS	ANHYDRATE
EVAZEG	ANHYDRATE
EVEFAN	ANHYDRATE
EVIHUM	ANHYDRATE
EVUQAO	SALT HYDRATE
EWACAG	SOLVATE
EWAMAR	ANHYDRATE
EWAPAU	COCRYSTAL
EXAPID	COCRYSTAL

SAJVEE	ANHYDRATE
SAKMEW	ANHYDRATE
SAKPOJ	ANHYDRATE
SALCAN	ANHYDRATE
SALHOC	SALT
SALIAZ	ANHYDRATE
SALMID	ANHYDRATE
SALOXM	ANHYDRATE
SAMCAL	ANHYDRATE
SAMPYM	ANHYDRATE
SANACM	HYDRATE
SAPJAV	ANHYDRATE
SAPVAI	ANHYDRATE
SARCAC	SALT
SASFAU	ANHYDRATE
SASKII	SALT
SATART	SALT HYDRATE
SATCOG	ANHYDRATE
SATPIO	SALT
SATRAG	SALT
SATSUD	SALT
SAWKIL	ANHYDRATE
SAXDIE	ANHYDRATE
SAXJEG	ANHYDRATE
SAXPOW	ANHYDRATE
SAYMUB	COCRYSTAL
SAYNAI	ANHYDRATE
SAYVEV	ANHYDRATE
SAZHOQ	SALT
SAZLEL	ANHYDRATE
SAZXAS	HYDRATE
SEBTAU	ANHYDRATE
SECZAB	ANHYDRATE
SEDTUQ	ANHYDRATE
SEGJAP	ANHYDRATE
SEGKEW	SALT SOLVATE

EXEKEX	SALT
EXIBOC	SALT
EXUQUJ	COCRYSTAL
EYIBEU	SALT
EYITAH	ANHYDRATE
EYOCUQ	ANHYDRATE
EYOGUV	ANHYDRATE
EZAQEB	ANHYDRATE
EZEFEV	SALT
EZIZAP	ANHYDRATE
FABDER	SOLVATE
FABFUJ	ANHYDRATE
FABMEA	SALT COCRYSTAL
FABRAB	ANHYDRATE
FABRIJ	SOLVATE
FABSUW	SOLVATE
FACCET	ANHYDRATE
FACCOB	ANHYDRATE
FACNUS	ANHYDRATE
FACRIK	ANHYDRATE
FACSEI	ANHYDRATE
FADWUC	ANHYDRATE
FAFMOQ	ANHYDRATE
FAFQEK	ANHYDRATE
FAFWIS	ANHYDRATE
FAGVEO	SALT HYDRATE
FAHLEF	COCRYSTAL
FAHNOR	ANHYDRATE
FAHVOZ	ANHYDRATE
FAJTIT	ANHYDRATE
FAKRIS	ANHYDRATE
FAMDIG	ANHYDRATE
FAQTIA	ANHYDRATE
FARPOF	ANHYDRATE
FARRUM	ANHYDRATE
FARWIF	SALT

SEGQUS	ANHYDRATE
SEHNAW	ANHYDRATE
SEINDI	ANHYDRATE
SEJWUZ	ANHYDRATE
SEMBOB	ANHYDRATE
SEMYIT	ANHYDRATE
SENGEX	ANHYDRATE
SENGOH	ANHYDRATE
SEOTCR	COCRYSTAL
SEPBAS	ANHYDRATE
SEPCAT	ANHYDRATE
SEPDIA	ANHYDRATE
SEPTIS	ANHYDRATE
SEQHUS	ANHYDRATE
SESHUT	ANHYDRATE
SESPEL	ANHYDRATE
SESYAR	ANHYDRATE
SETDTC	ANHYDRATE
SETSAN	COCRYSTAL
SEVGUV	ANHYDRATE
SEVPEP	ANHYDRATE
SEYDIJ	HYDRATE
SEYLEN	SALT HYDRATE
SEYXUR	ANHYDRATE
SEYYIG	ANHYDRATE
SEYZON	SALT
SIBTAZ	ANHYDRATE
SICXIL	ANHYDRATE
SIDHOD	ANHYDRATE
SIDJEU	ANHYDRATE
SIDSUT	ANHYDRATE
SIFLOI	ANHYDRATE
SIGFOE	SALT
SIHBIV	SALT HYDRATE
SIHNII	ANHYDRATE
SIHVOU	ANHYDRATE

FAWFOY	ANHYDRATE
FAWSAZ	SALT
FAXBUB	ANHYDRATE
FAXPOJ	SALT
FAXVAB	ANHYDRATE
FAXVOQ	ANHYDRATE
FAYNUO	SALT
FAZTAC	ANHYDRATE
FBPAZD	ANHYDRATE
FECZOE	ANHYDRATE
FEFKIL	SALT
FEFKIM	SALT
FEFPEM	ANHYDRATE
FEFQUD	ANHYDRATE
FEFREP	ANHYDRATE
FEFSAK	ANHYDRATE
FEFXOF	SALT
FEGTEQ	SALT HYDRATE
FEGWAP	ANHYDRATE
FEHKOS	ANHYDRATE
FEJNOZ	ANHYDRATE
FEMMAL	ANHYDRATE
FENPUJ	SOLVATE
FENXUR	SALT
FEPHAL	ANHYDRATE
FEPJOB	ANHYDRATE
FEPROJ	ANHYDRATE
FEQDIQ	ANHYDRATE
FEQPAS	ANHYDRATE
FEQVUT	ANHYDRATE
FESCOW	ANHYDRATE
FESKAP	ANHYDRATE
FESNEW	ANHYDRATE
FETPIF	ANHYDRATE
FEVBUD	ANHYDRATE
FEVCAK	ANHYDRATE

SIKLIH	ANHYDRATE
SILTOW	ANHYDRATE
SILTUC	ANHYDRATE
SILVAL	ANHYDRATE
SILXUG	ANHYDRATE
SIMXUI	ANHYDRATE
SINDIG	ANHYDRATE
SINZIY	ANHYDRATE
SIRDUT*	ANHYDRATE
SIRJUZ	ANHYDRATE
SIRMIQ	ANHYDRATE
SIRXUM	ANHYDRATE
SISSOE	ANHYDRATE
SITDII	SOLVATE
SITLUD	SALT
SIVBEE	ANHYDRATE
SIVCAB	ANHYDRATE
SIWDEH	ANHYDRATE
SIWDIL	ANHYDRATE
SIWZOP	SALT
SIXKAN	ANHYDRATE
SIYDOV	SALT
SIYNET	SOLVATE
SLFNMA	ANHYDRATE
SLFNMB	ANHYDRATE
SOBJAV	SALT HYDRATE
SOBNEE	COCRYSTAL
SOBPEE	ANHYDRATE
SOBRAE	ANHYDRATE
SOBSUX	ANHYDRATE
SOCRUZ	ANHYDRATE
SOCSIO	SALT
SOCTOT	ANHYDRATE
SODCIX	ANHYDRATE
SODDUL	ANHYDRATE
SODKAZ	ANHYDRATE

FEVKAT	ANHYDRATE
FEVMUO	ANHYDRATE
FEVNAV	ANHYDRATE
FEVWUY	ANHYDRATE
FEXRIK	ANHYDRATE
FEYBIU	ANHYDRATE
FEZGUM	ANHYDRATE
FIBKUW	ANHYDRATE
FIBYIY	ANHYDRATE
FICGEE	ANHYDRATE
FIDYIA	SALT
FIFBEB	SALT SOLVATE
FIFFAB	ANHYDRATE
FIFGOQ	ANHYDRATE
FIGYID	ANHYDRATE
FIHNUH	ANHYDRATE
FIJBUW	ANHYDRATE
FIKFIO	ANHYDRATE
FIKJEQ	ANHYDRATE
FIKZOO	ANHYDRATE
FILBEH	HYDRATE
FILGEM	ANHYDRATE
FILHAJ	ANHYDRATE
FIMNAQ	ANHYDRATE
FIRXUA	ANHYDRATE
FISSUV*	ANHYDRATE
FITQII	COCRYSTAL
FIWZUG	HYDRATE
FIYBEV	ANHYDRATE
FIZPEL	ANHYDRATE
FIZRUD	ANHYDRATE
FIZWOC	ANHYDRATE
FLUBIP*	ANHYDRATE
FLYITU	ANHYDRATE
FMDURD	ANHYDRATE
FOBBIH	ANHYDRATE

SOFPAG	ANHYDRATE
SOGUAN	HYDRATE
SOHXAO	ANHYDRATE
SOHXIY	SALT
SOLBEC	COCRYSTAL
SOLPUF	ANHYDRATE
SOMKIQ	SALT
SOMKOU	ANHYDRATE
SONSUK	SALT
SOPRUK	ANHYDRATE
SOSMAO	ANHYDRATE
SOVFOA	SALT
SOVWIJ	ANHYDRATE
SOVXUY	ANHYDRATE
SOVYOT	SOLVATE
SOVZAG	SOLVATE
SOYSOP	ANHYDRATE
SPSNAP	ANHYDRATE
SRFORA	SALT
SSOXAM*	ANHYDRATE
STARAC	ANHYDRATE
STPYAZ	ANHYDRATE
SUBTIS	ANHYDRATE
SUCACB	ANHYDRATE
SUCHOL	SALT
SUCNOT	ANHYDRATE
SUCROS	ANHYDRATE
SUDXAS	ANHYDRATE
SUKNIW	ANHYDRATE
SULAMD	ANHYDRATE
SUPBEK	ANHYDRATE
SUPFEO	ANHYDRATE
SUQFIT	ANHYDRATE
SUQWIK	ANHYDRATE
SURGOC	ANHYDRATE
SUTHAZ	ANHYDRATE

FOBCAA	SALT
FOBMIU	ANHYDRATE
FOBSOE	ANHYDRATE
FOCWUR	ANHYDRATE
FOFZEH	COCRYSTAL
FOGVIG	ANHYDRATE
FOHLIY	SALT
FOJMOG	ANHYDRATE
FOLBUE	ANHYDRATE
FOLPEB	ANHYDRATE
FOMNEB	ANHYDRATE
FOMVIO	ANHYDRATE
FONFAQ	SOLVATE
FOPBAO	ANHYDRATE
FOQJEC	ANHYDRATE
FOQZAM	ANHYDRATE
FORBZA	ANHYDRATE
FORMAC	ANHYDRATE
FOSFUQ	COCRYSTAL
FOSMIL	COCRYSTAL
FOVBIC	ANHYDRATE
FOVRUE	ANHYDRATE
FOVYOE	ANHYDRATE
FOWZUN	ANHYDRATE
FOXLOT	SALT HYDRATE
FOXNAH	SALT
FOXNEL	SALT
FOXNUB	SALT
FOXREQ	ANHYDRATE
FOYNEO	ANHYDRATE
FOZVOF	ANHYDRATE
FPAMCA	ANHYDRATE
FSEANT	ANHYDRATE
FUCKOD	HYDRATE
FUCTIG	SALT
FUDTON	SALT

SUWLAX	ANHYDRATE
SUWMIG	ANHYDRATE
SUZGIE	ANHYDRATE
SUZYES	ANHYDRATE
TABCAC	SALT
TACRIB	ANHYDRATE
TADLIU	ANHYDRATE
TADTAU	ANHYDRATE
TADZAZ	SALT
TAFBAD	SOLVATE
TAFBUZ	SALT COCRYSTAL
TAGZOQ	ANHYDRATE
TAJCUD	SALT
TAJVAC	ANHYDRATE
TAJVIK	ANHYDRATE
TALJIZ	ANHYDRATE
TAMBUE	COCRYSTAL
TAMZIQ	ANHYDRATE
TAPZIT	ANHYDRATE
TAQCET	ANHYDRATE
TAQDIZ	ANHYDRATE
TARJIG	ANHYDRATE
TARNIL	ANHYDRATE
TARTMM	HYDRATE
TASCUM	ANHYDRATE
TASVIS	ANHYDRATE
TAWRIT	ANHYDRATE
TAYTUI	ANHYDRATE
TAZPEN	ANHYDRATE
TBCHTS	ANHYDRATE
TBMNBZ	ANHYDRATE
TBTYAC	ANHYDRATE
TCBENQ	ANHYDRATE
TCLBEN	ANHYDRATE
TCLOBQ	ANHYDRATE
TCLTET	ANHYDRATE

FUFSIJ	ANHYDRATE
FUFTEG	ANHYDRATE
FUGJUM	ANHYDRATE
FUGMIF	SALT
FUGTIK	ANHYDRATE
FUGYAH	ANHYDRATE
FUMAAC	ANHYDRATE
FUQJIK	ANHYDRATE
FUQLIM	ANHYDRATE
FUQMOU	SALT
FUQWAQ	ANHYDRATE
FURACL	ANHYDRATE
FURHUV	ANHYDRATE
FURSEM	ANHYDRATE
FURWIA	COCRYSTAL
FUSWOH	HYDRATE
FUWWOL	SALT COCRYSTAL
FUYJAK	ANHYDRATE
FUYVUQ	ANHYDRATE
FUZMET	ANHYDRATE
FUZPUL	ANHYDRATE
GACFOG	ANHYDRATE
GADSIO	ANHYDRATE
GADTOV	ANHYDRATE
GAFFAV	SALT
GAFPAE	ANHYDRATE
GAFPEI	ANHYDRATE
GAFXAN	ANHYDRATE
GAGSIR	ANHYDRATE
GAGVAL	ANHYDRATE
GAKNAH	ANHYDRATE
GAKWIA	ANHYDRATE
GALCAX	ANHYDRATE
GAMBUT	ANHYDRATE
GAMJUZ	SALT HYDRATE
GANYEA*	COCRYSTAL

TCTMBE	ANHYDRATE
TCYETY	ANHYDRATE
TDBOCP	ANHYDRATE
TEBGAI	ANHYDRATE
TECCAF	COCRYSTAL
TECXII	ANHYDRATE
TECZAE	SOLVATE
TEDAPC	SALT
TEDFUF	SALT
TEEXAN	ANHYDRATE
TEGNEA	SALT
TEHGUI	ANHYDRATE
TEHKOG	ANHYDRATE
TEHMEY	ANHYDRATE
TEHNAW	COCRYSTAL
TEHNID	ANHYDRATE
TEHTUX	ANHYDRATE
TEJKUO	SALT
TEKMED	ANHYDRATE
TELKUQ	ANHYDRATE
TELYAK	ANHYDRATE
TEMBES	ANHYDRATE
TENJAX	ANHYDRATE
TENQUA	SALT
TEPHTH	ANHYDRATE
TEPNIT	ANHYDRATE
TEQMUX	SALT
TERMEI	SALT HYDRATE
TESTOM	HYDRATE
TETAMI	SALT
TETBBZ	ANHYDRATE
TETBEZ	ANHYDRATE
TETNIP	ANHYDRATE
TETROL	ANHYDRATE
TETUMS	ANHYDRATE
TETZOL	ANHYDRATE

GANYOK	SALT
GAQHIR	ANHYDRATE
GASFAH	ANHYDRATE
GAXLEW	ANHYDRATE
GAXXUY	SALT
GAYTIJ	ANHYDRATE
GEBTUC	ANHYDRATE
GEDKEH	ANHYDRATE
GEFREO	SOLVATE
GEGGEF	ANHYDRATE
GEGNUB	ANHYDRATE
GEGXAS	ANHYDRATE
GEHBAX	SALT COCRYSTAL
GEKLOX	SOLVATE
GEKMOA	SALT
GEKZIF	SALT
GELDEI	ANHYDRATE
GEMGAG	ANHYDRATE
GEMMAN	ANHYDRATE
GENLAN	ANHYDRATE
GENQIB	ANHYDRATE
GEPPAU	ANHYDRATE
GEPXOO	ANHYDRATE
GERPOJ	ANHYDRATE
GERZAG	SALT
GESBIR	COCRYSTAL
GESNUN	ANHYDRATE
GETFUG	ANHYDRATE
GEXXIQ	ANHYDRATE
GEZREK	ANHYDRATE
GICTIV	ANHYDRATE
GICVUJ	ANHYDRATE
GIDHEG*	ANHYDRATE
GIFPAM	ANHYDRATE
GIFSOE	ANHYDRATE
GIGXAX	SALT COCRYSTAL

TEVFIK	ANHYDRATE
TEVJOU	ANHYDRATE
TEVSOD	ANHYDRATE
TEYHIQ	ANHYDRATE
TGLYBE	SALT
TGLYSU	SALT
THALID	ANHYDRATE
THBARB	ANHYDRATE
THEOPI	SALT
THHYDT	ANHYDRATE
THIAMC*	SALT HYDRATE
THIOUR	ANHYDRATE
THUTEC	SALT
THXMAM	ANHYDRATE
TIBGAN	ANHYDRATE
TICDIT	ANHYDRATE
TICDOZ	ANHYDRATE
TICHUI	ANHYDRATE
TICNOI	ANHYDRATE
TIETHE	ANHYDRATE
TIFDAN	SALT COCRYSTAL
TIFLAV	SALT
TIJKIH	SALT
TIJXUH	SALT
TIJZIV	ANHYDRATE
TIKBEU	SALT
TIKFAW	ANHYDRATE
TILROV	ANHYDRATE
TINBIB	ANHYDRATE
TINJAB	ANHYDRATE
TIPDEB	ANHYDRATE
TIQKEJ	ANHYDRATE
TIRVOH	SALT
TITSIA	SALT
TITVAU	ANHYDRATE
TIVDUZ	SALT

GIGYEA	ANHYDRATE
GIHWOJ	SALT
GIJTEA	ANHYDRATE
GIJTUO	SALT
GIJVIG	SALT
GIKBOT	ANHYDRATE
GIKKAO	ANHYDRATE
GIMBUZ	ANHYDRATE
GIMNIA	ANHYDRATE
GIMPOI	SALT
GINCAI	ANHYDRATE
GINKIZ	ANHYDRATE
GIRBUF	ANHYDRATE
GIRNOL	ANHYDRATE
GISNOM*	ANHYDRATE
GISNUS	ANHYDRATE
GISRIJ	SALT
GISYOX	ANHYDRATE
GISZIR	ANHYDRATE
GITXAK	ANHYDRATE
GIVJUQ	ANHYDRATE
GIXHUR	ANHYDRATE
GIXPEI	SALT SOLVATE
GIYDEZ	SALT
GIZFIF	ANHYDRATE
GIZYIY	ANHYDRATE
GLUCIT	ANHYDRATE
GLUCSA	ANHYDRATE
GLURAC	ANHYDRATE
GLUTAS	ANHYDRATE
GLYCIN	ANHYDRATE
GLYGLY	ANHYDRATE
GNAPHB	ANHYDRATE
GOBVEA	ANHYDRATE
GOBYAX	ANHYDRATE
GOCJIT	ANHYDRATE

TIVKOZ	ANHYDRATE
TIVXUS	ANHYDRATE
TIWYEF	ANHYDRATE
TIWYIH	ANHYDRATE
TIXHAJ	SALT
TIXTUQ	SALT
TIXVAY	ANHYDRATE
TIYQAU	ANHYDRATE
TIZWAA	ANHYDRATE
TMAMMC	SALT
TMAMOH	SALT HYDRATE
TMAPCL	SALT
TMESPH	ANHYDRATE
TMETTS	ANHYDRATE
TMPDPC	SALT
TMPPIO	ANHYDRATE
TNBENZ	ANHYDRATE
TNFLUO	ANHYDRATE
TOBRUX	ANHYDRATE
TOCPIM	ANHYDRATE
TOHBUN	ANHYDRATE
TOHHED	ANHYDRATE
TOHTUG	ANHYDRATE
TOHVAO	ANHYDRATE
TOKQEP	SALT
TOKSAO	ANHYDRATE
TOKSES	SALT
TOLKEK	ANHYDRATE
TOLPHO*	ANHYDRATE
TOLPSO	ANHYDRATE
TOLSAM	SALT HYDRATE
TONDUV	COCRYSTAL
TOQKIV	ANHYDRATE
TORSEM	ANHYDRATE
TORTEZ	ANHYDRATE
TOTYIM	SALT

GOCPAP	SALT SOLVATE
GOFVAZ	ANHYDRATE
GOGQOJ	ANHYDRATE
GOKCOA	ANHYDRATE
GOKQAY	SOLVATE HYDRATE
GOLLUO	ANHYDRATE
GOPDUK	ANHYDRATE
GOQCEW	SALT
GOQPOR	SALT
GORBOE	ANHYDRATE
GORKUV	SALT
GORVIU	ANHYDRATE
GORXAM	SOLVATE
GORXEQ	SOLVATE
GOXJOS	ANHYDRATE
GOXWAR	ANHYDRATE
GUBJOE	SALT
GUCNUP	SALT
GUCWOQ	ANHYDRATE
GUCYAE	ANHYDRATE
GUFMOJ	ANHYDRATE
GUHDIY	ANHYDRATE
GUHHAS	ANHYDRATE
GUJNAA	ANHYDRATE
GUKPEH	ANHYDRATE
GUMXAO	ANHYDRATE
GUQRIU	ANHYDRATE
GUQZUP	ANHYDRATE
GURBUQ	ANHYDRATE
HABFAS	ANHYDRATE
HABJUP	SALT HYDRATE
HACTPH	ANHYDRATE
HADKIG	ANHYDRATE
HADKUT	COCRYSTAL
HADNOP	ANHYDRATE
HAFVIV*	ANHYDRATE

TOXGLU	ANHYDRATE
TOZKOI	ANHYDRATE
TPASTB	SALT
TPEPHO	ANHYDRATE
TPHPOR	ANHYDRATE
TPPHSE	ANHYDRATE
TPPOSS	ANHYDRATE
TRDECA	ANHYDRATE
TRDMPP	ANHYDRATE
TRPHAM	ANHYDRATE
TSCARB	ANHYDRATE
TSCPCP	ANHYDRATE
TTFCAN*	COCRYSTAL
TTHCTD	ANHYDRATE
TUBDUP	SALT
TUBYIY	ANHYDRATE
TUCKUZ	SALT
TUDQEQ	ANHYDRATE
TUDROZ	ANHYDRATE
TUHBAZ	ANHYDRATE
TUHXID	ANHYDRATE
TUMDIP	ANHYDRATE
TUMPOG	SOLVATE
TUPGUH	ANHYDRATE
TUQCOX	ANHYDRATE
TURPUR	SALT HYDRATE
TURPYB	SALT COCRYSTAL
TURSAB	ANHYDRATE
TURTEB	SALT
TUTBUF	ANHYDRATE
TUVXAL	SALT
TUWFIB	ANHYDRATE
TUWHEZ	SALT
TUXJIF	ANHYDRATE
UBABAC	ANHYDRATE
UBEDUA	ANHYDRATE

HAJSAO	SALT HYDRATE
HAJYUN	ANHYDRATE
HAKTAP	ANHYDRATE
HAKVAR	SOLVATE
HAKVUL	SOLVATE
HALMEO	ANHYDRATE
HALMUC	ANHYDRATE
HAMNEO	SALT
HANFOS	ANHYDRATE
HAVBIQ	ANHYDRATE
HAWRAZ	ANHYDRATE
HAXBUD	HYDRATE
HAXHET	ANHYDRATE
HAXKIC	ANHYDRATE
HAXLAV	ANHYDRATE
HAXMAW	ANHYDRATE
HAXVAF	SALT
HAYKUO	ANHYDRATE
HAZBEQ*	ANHYDRATE
HAZFAP	SALT
HCSBTZ	ANHYDRATE
HDXMOR	ANHYDRATE
HEBFUR	ANHYDRATE
HEBQOV	SALT
HEBWIV	SALT
HEGPAK	COCRYSTAL
HEHDOP	SALT COCRYSTAL
HELBAD	SALT COCRYSTAL
HEPJAN	ANHYDRATE
HEPJOC	ANHYDRATE
HEQFIT	ANHYDRATE
HESDIT	ANHYDRATE
HETDAM	SOLVATE
HETPAL	ANHYDRATE
HEVWAI	ANHYDRATE
HEVXUB	ANHYDRATE

UBEFEO*	SALT
UBUJIM	COCRYSTAL
UBUQIR	ANHYDRATE
UCAYED	ANHYDRATE
UCAYIH	ANHYDRATE
UCECAG	ANHYDRATE
UCENOG	ANHYDRATE
UCEQID	ANHYDRATE
UCIJOF	ANHYDRATE
UCITEF	ANHYDRATE
UCUGOP	ANHYDRATE
UDAYUT	ANHYDRATE
UDETOM	SALT COCRYSTAL
UDEVOO	SALT COCRYSTAL
UDIHOE	HYDRATE
UDIPOM	ANHYDRATE
UDOXAN	ANHYDRATE
UFAGIS	ANHYDRATE
UFAMIY	ANHYDRATE
UFEBIS	ANHYDRATE
UFEZAH	ANHYDRATE
UFIKAX	ANHYDRATE
UFOCIC	ANHYDRATE
UFOGON	ANHYDRATE
UGAKUK	ANHYDRATE
UGEDOA	ANHYDRATE
UGIFOG	ANHYDRATE
UGOVIX	COCRYSTAL
UGOXEU	ANHYDRATE
UGOXIY	ANHYDRATE
UHAVAB	ANHYDRATE
UHAVEG	ANHYDRATE
UHAWIJ	SALT
UHENUQ	ANHYDRATE
UHIVEN	SALT HYDRATE
UHULEQ	ANHYDRATE

HEWLOL	ANHYDRATE
HEXCET	ANHYDRATE
HEXWIQ	ANHYDRATE
HEYHUO	ANHYDRATE
HEYQOR	SALT
HEZZIV	SALT
HIBCOK	SALT
HIBGUV	ANHYDRATE
HIBVAP	ANHYDRATE
HIBXOF	ANHYDRATE
HICJAG	ANHYDRATE
HICJIO	ANHYDRATE
HICYAT	ANHYDRATE
HICZEY	ANHYDRATE
HICZIC	ANHYDRATE
HICZUO	ANHYDRATE
HIDDAZ	ANHYDRATE
HIDHAD	ANHYDRATE
HIGBII	ANHYDRATE
HIGNIV	ANHYDRATE
HIHKUE	ANHYDRATE
HIHROF	ANHYDRATE
HIJFIP	ANHYDRATE
HIKNEW	ANHYDRATE
HIMGOA	ANHYDRATE
HIMWIJ	ANHYDRATE
HIRPAZ	ANHYDRATE
HIRQIK	ANHYDRATE
HIRQUW	ANHYDRATE
HIRREH	ANHYDRATE
HIRROR	ANHYDRATE
HIRYIR	ANHYDRATE
HISGAS	ANHYDRATE
HISRIL	ANHYDRATE
HISROR	ANHYDRATE
HISRUW	ANHYDRATE

UHUMEP	ANHYDRATE
UHURAR	ANHYDRATE
UJACAL	ANHYDRATE
UJACIT	ANHYDRATE
UJIRIO	ANHYDRATE
UJODUT	SALT
UJOGUW	SALT
UJORIU	ANHYDRATE
UKAKEW	SOLVATE
UKANOJ	ANHYDRATE
UKOSAP	COCRYSTAL
UKOYUO	ANHYDRATE
UKUQOH	ANHYDRATE
ULALUO	ANHYDRATE
ULAWAF	COCRYSTAL
ULERAE	SALT
UMOMAL	ANHYDRATE
UNEVOZ	ANHYDRATE
UNEWIU	ANHYDRATE
UNEWUF	ANHYDRATE
UNEYIV	SOLVATE
UNEZAO	COCRYSTAL
UNIRUF	ANHYDRATE
UNISEQ	ANHYDRATE
UNISIU*	ANHYDRATE
UNOGIN	ANHYDRATE
UPOYEE	SALT SOLVATE
UQOBIM	ANHYDRATE
URAQEK	ANHYDRATE
URAZIX	ANHYDRATE
USIZAY	ANHYDRATE
UTIXAX	ANHYDRATE
UTORAX	ANHYDRATE
UTORIF	ANHYDRATE
UVEXID	ANHYDRATE
UWUKAZ	SALT

HIVROT*	SALT
HIWJIG	ANHYDRATE
HIXHIF	ANHYDRATE
HMBENZ-D	ANHYDRATE
HMHOCN	ANHYDRATE
HNIABZ	ANHYDRATE
HODWED	ANHYDRATE
HOFFEN	ANHYDRATE
HOFLOE	ANHYDRATE
HOFMAQ	ANHYDRATE
HOFNOF	ANHYDRATE
HOFNOH	SOLVATE
HOFWAB	ANHYDRATE
HOHMOG	SALT
HOHRIF	ANHYDRATE
HOJQEE	ANHYDRATE
HOJQII*	ANHYDRATE
HOJROP	ANHYDRATE
HOKMAW	ANHYDRATE
HOKPUS	ANHYDRATE
HOLJAU	COCRYSTAL
HOLLIE	ANHYDRATE
HOLWAH	ANHYDRATE
HOLWAI	COCRYSTAL
HOQABS	ANHYDRATE
HOQSUB	ANHYDRATE
HORCAS	ANHYDRATE
HORREM	SALT
HORTAJ	ANHYDRATE
HORTUE	ANHYDRATE
HORXOB	COCRYSTAL
HOZSAQ	ANHYDRATE
HPCBDP	ANHYDRATE
HPHBNZ	ANHYDRATE
HPTSIO	ANHYDRATE
HUBSUT	SALT

UXAGAC	SALT SOLVATE
UXEGEK	HYDRATE
UXIXAB	SALT
UXIXIJ	SALT
UXIYOQ	ANHYDRATE
UXIZAD	ANHYDRATE
UYOYIR	SALT HYDRATE
UZENIX	ANHYDRATE
UZOVAH	SALT
VACTAU	ANHYDRATE
VAGDOX	SALT
VAGPOJ	ANHYDRATE
VAHTAB	ANHYDRATE
VAKPUU	ANHYDRATE
VAKTOS	COCRYSTAL
VALIDL	ANHYDRATE
VALLIE	ANHYDRATE
VALPII	ANHYDRATE
VALSUY	ANHYDRATE
VALTEJ	ANHYDRATE
VAMBOA	ANHYDRATE
VAPKIG	ANHYDRATE
VARCIA*	ANHYDRATE
VARFAX	ANHYDRATE
VARYIW	ANHYDRATE
VASXES	SALT
VATRIT	ANHYDRATE
VATSAK	ANHYDRATE
VATSOA	SALT
VATXOF	COCRYSTAL
VAWDOM	ANHYDRATE
VAWKUA	SOLVATE
VAXTIZ	ANHYDRATE
VAXXOH	ANHYDRATE
VAXZIF	ANHYDRATE
VAYKUB	SALT

HUCHEU	SALT
HUDCOZ	ANHYDRATE
HUDHUK	ANHYDRATE
HUHDUK	COCRYSTAL
HUKHUQ	ANHYDRATE
HUKQEJ	SALT
HUMFIG	ANHYDRATE
HUMLUW	ANHYDRATE
HUMTEP	ANHYDRATE
HUPGOO	ANHYDRATE
HURJUZ	SALT
HUSVIB	ANHYDRATE
HUTQAP	ANHYDRATE
HUVVOJ	SALT HYDRATE
HUWSOI	ANHYDRATE
HUYRIC	SALT
HXACAN	ANHYDRATE
HXDYNB	ANHYDRATE
HXOPEN	ANHYDRATE
HXPHOL	ANHYDRATE
HXQUIN	ANHYDRATE
HYDRZN	SALT
HYQUIN	ANHYDRATE
IBBIOX	ANHYDRATE
IBECUO	ANHYDRATE
IBIJAF	ANHYDRATE
IBOBUY	ANHYDRATE
IBOCEJ	ANHYDRATE
IBOCOT	ANHYDRATE
IBOMAP	ANHYDRATE
IBOXAA	SOLVATE
IBPRAC	ANHYDRATE
IBULIA	ANHYDRATE
IBUSIJ	ANHYDRATE
IBUXAF	SOLVATE
IBZFRO	ANHYDRATE

VAYSET	ANHYDRATE
VAZCOP	ANHYDRATE
VAZRIX	ANHYDRATE
VEBBUA	ANHYDRATE
VECRUR	ANHYDRATE
VEDQOL	ANHYDRATE
VEFWOS	ANHYDRATE
VEGHIY	SALT
VEGMAV	ANHYDRATE
VEGNAX	ANHYDRATE
VEJCIW	ANHYDRATE
VEKSOT	ANHYDRATE
VEKYAL	ANHYDRATE
VEMTOW	ANHYDRATE
VENMUW	ANHYDRATE
VEQNEK	ANHYDRATE
VERNOV	ANHYDRATE
VEVLIR	SOLVATE
VEXJEN	ANHYDRATE
VEXNUI	ANHYDRATE
VEYGEL	ANHYDRATE
VIBMOJ	SALT
VICPEC	ANHYDRATE
VICQAB	ANHYDRATE
VIDMAX*	ANHYDRATE
VIFGIA	ANHYDRATE
VIFLON	SALT
VIFQIL	ANHYDRATE
VIHGID	ANHYDRATE
VIJGAY	ANHYDRATE
VIMJIM	ANHYDRATE
VIMPAJ	ANHYDRATE
VIOLME	HYDRATE
VIPBED	ANHYDRATE
VIPKIO	ANHYDRATE
VIQFUY	ANHYDRATE

ICAHIE	ANHYDRATE
ICAMOP	ANHYDRATE
ICEPOX	SALT
ICEWIW	ANHYDRATE
ICOFEM	SALT
ICOLOC	ANHYDRATE
ICOMUI	SALT HYDRATE
ICOZOR	ANHYDRATE
ICUBAL	ANHYDRATE
ICULEY	ANHYDRATE
ICUTOR	ANHYDRATE
IDALEF	ANHYDRATE
IDEPIR	ANHYDRATE
IDOBAG	ANHYDRATE
IDONAS*	ANHYDRATE
IFAWAP	ANHYDRATE
IFILAL	ANHYDRATE
IFIZIG	HYDRATE
IFOVOO	ANHYDRATE
IFULUQ	ANHYDRATE
IFUTOT	ANHYDRATE
IFUXEN	ANHYDRATE
IGALUY	ANHYDRATE
IGASIS	ANHYDRATE
IGUQEG	ANHYDRATE
IHAPEO	ANHYDRATE
IHAPOX	ANHYDRATE
IHEKOV	HYDRATE
IHEMIR	ANHYDRATE
IHIKIV	ANHYDRATE
IHOSUT	ANHYDRATE
IHOZOW	ANHYDRATE
IHXNAQ	ANHYDRATE
IJAJUY	ANHYDRATE
IJAXAT*	SOLVATE
IJESUL	ANHYDRATE

VIRAZL	ANHYDRATE
VISKAJ	SALT
VISQOF	ANHYDRATE
VISSOF	ANHYDRATE
VITAAC	ANHYDRATE
VITMAN	ANHYDRATE
VITQUM	SALT
VITROH	ANHYDRATE
VIVQOH	SALT HYDRATE
VIVTAW	SALT SOLVATE
VIWHAM	ANHYDRATE
VIZHIW	ANHYDRATE
VIZYUA	ANHYDRATE
VOBNAB	ANHYDRATE
VODLEG	SALT
VOGCOL	ANHYDRATE
VOHKEK*	COCRYSTAL
VOHNOV	ANHYDRATE
VOLJIR	ANHYDRATE
VOLZEC	ANHYDRATE
VOMWUR	SALT
VONYOO	SALT
VOPJUH	ANHYDRATE
VOQHIU	ANHYDRATE
VOQHOA	ANHYDRATE
VOQNIY	SALT
VOQXOP*	ANHYDRATE
VOSQUP	ANHYDRATE
VOXDUH	SALT
VOXFUJ	ANHYDRATE
VUBQAM	ANHYDRATE
VUBYAU	ANHYDRATE
VUFGEI	ANHYDRATE
VUFKAJ	ANHYDRATE
VUFKEM	ANHYDRATE
VUHFIO	COCRYSTAL

IJETEW	COCRYSTAL
IJETOG	COCRYSTAL
IJIBEJ	COCRYSTAL
IJOQED	ANHYDRATE
IJUDAT	HYDRATE
IJUDUN	ANHYDRATE
IJUMEG	ANHYDRATE
IJUXEQ	ANHYDRATE
ILIMEV	ANHYDRATE
IMAZOL	ANHYDRATE
IMDIAC	ANHYDRATE
IMEROI	ANHYDRATE
IMIPAV	ANHYDRATE
IMPTHA	ANHYDRATE
IMUTEQ	ANHYDRATE
INAHZC	SALT
INDBUA	ANHYDRATE
INDIGO	ANHYDRATE
INDMET	ANHYDRATE
INELIW	SOLVATE
INEQUN	ANHYDRATE
INOBUI	SOLVATE
INOCET	ANHYDRATE
INODUK	ANHYDRATE
INOHIC	ANHYDRATE
INOSIN	ANHYDRATE
INUKAD	SOLVATE
IOBNZA	ANHYDRATE
IODOFO	ANHYDRATE
IPATOJ	ANHYDRATE
IPAWAY	ANHYDRATE
IPOPQU	ANHYDRATE
IPOZAO	SALT
IPUPUE	ANHYDRATE
IQAREY	ANHYDRATE
IQIDIW	ANHYDRATE

VUJMAO	ANHYDRATE
VUJNAP	SALT
VUJSOJ	COCRYSTAL
VUJXIH	SALT
VUJZAB	ANHYDRATE
VUKGAJ	ANHYDRATE
VULCIQ	SOLVATE
VUNYEI	ANHYDRATE
VUNYUY	ANHYDRATE
VUPTAC	ANHYDRATE
VURNAX	ANHYDRATE
VURWAH	SALT
VURYOW	ANHYDRATE
VUSDIX	ANHYDRATE
VUSFIY	ANHYDRATE
VUSZEO	ANHYDRATE
VUTGOG	ANHYDRATE
VUTZIT	ANHYDRATE
VUXRIQ	ANHYDRATE
VUXZEU	ANHYDRATE
VUZPAH	ANHYDRATE
VUZWAO	ANHYDRATE
WABZEE	SALT
WACHEP	ANHYDRATE
WAJRIK	SALT
WAJSIL	SALT
WAJTEI	SALT
WAJXEL	ANHYDRATE
WAKCIW	ANHYDRATE
WAKLUQ	ANHYDRATE
WAMDOD	ANHYDRATE
WAMFAS	ANHYDRATE
WAMXOX	ANHYDRATE
WANLON	ANHYDRATE
WANMUU	ANHYDRATE
WANNUV	COCRYSTAL

IQILAW	ANHYDRATE
IRIMEB	SALT HYDRATE
IRUFEH	ANHYDRATE
IRUQOB	ANHYDRATE
ISALOE	ANHYDRATE
ISAYEH	ANHYDRATE
ISICUJ	ANHYDRATE
ISIDEU	ANHYDRATE
ISUNAL	ANHYDRATE
IURACL	ANHYDRATE
IVADUE	ANHYDRATE
IVEJUO	ANHYDRATE
IVOLEL	ANHYDRATE
IVUQOF	ANHYDRATE
IWEJOK	ANHYDRATE
IWUKAM	ANHYDRATE
IXOGAD	ANHYDRATE
IXOYUQ	SALT
IYAWIO	ANHYDRATE
IYEBUJ	ANHYDRATE
IYEJIF	ANHYDRATE
IZANEC	ANHYDRATE
IZANOM	ANHYDRATE
IZAPEE	ANHYDRATE
JABSAG	ANHYDRATE
JABZAN	ANHYDRATE
JACREL	SALT
JAFGUS	ANHYDRATE
JAHKUZ	ANHYDRATE
JAKGEH	ANHYDRATE
JAKKUB	SOLVATE
JAKQAO	ANHYDRATE
JALBII	ANHYDRATE
JAMRIY	SALT
JAPGUC	SALT
JAQJAN	ANHYDRATE

WANSAG	ANHYDRATE
WAPBUK	ANHYDRATE
WAPREL	ANHYDRATE
WAQTUF	SALT HYDRATE
WARRIS	ANHYDRATE
WASRAJ	ANHYDRATE
WASTEP	ANHYDRATE
WATREP	COCRYSTAL
WAVCUR	SALT
WAVZAV	ANHYDRATE
WAWDUU	SOLVATE
WAZTEY	ANHYDRATE
WEBCOV	SALT
WEBMAR	SOLVATE
WEBNUO	ANHYDRATE
WEBSIF	ANHYDRATE
WECCUC	ANHYDRATE
WECGIW	ANHYDRATE
WEDLEW	ANHYDRATE
WEDSEE	ANHYDRATE
WEFJUN	ANHYDRATE
WEFKEY	ANHYDRATE
WEFKIC	ANHYDRATE
WEFVIN	ANHYDRATE
WEHXUD	ANHYDRATE
WEKHEA	ANHYDRATE
WELHEA	ANHYDRATE
WELZUK	SOLVATE
WENVER	ANHYDRATE
WEPDEB	ANHYDRATE
WERNOW	ANHYDRATE
WERROA	ANHYDRATE
WERTUI	ANHYDRATE
WESBIF	ANHYDRATE
WETFAD	ANHYDRATE
WEWLUG	SALT COCRYSTAL

JARPEX	ANHYDRATE
JARQUP	ANHYDRATE
JARXUV	ANHYDRATE
JATFUF	ANHYDRATE
JAVCEO	ANHYDRATE
JAVWIN	ANHYDRATE
JAWQIH	SALT HYDRATE
JAWQUT	ANHYDRATE
JAWXEK	ANHYDRATE
JAYCES	ANHYDRATE
JAYPUU	SALT
JAZCOD	ANHYDRATE
JEBFEB	ANHYDRATE
JEDTOB	HYDRATE
JEGLAI	ANHYDRATE
JEJSIA	ANHYDRATE
JEKHEN	ANHYDRATE
JEMNUK	ANHYDRATE
JEMROJ	ANHYDRATE
JEMRUP	ANHYDRATE
JEMSAW	ANHYDRATE
JETLEA	ANHYDRATE
JEYDEW	ANHYDRATE
JEYWEQ	ANHYDRATE
JIBCIG	ANHYDRATE
JICTUK	COCRYSTAL
JIDTOF	SOLVATE
JIKDEM	ANHYDRATE
JILZOU	COCRYSTAL
JIMVUW	ANHYDRATE
JINGIW*	SALT
JINROO-D	ANHYDRATE
JINROO-H	ANHYDRATE
JIRCAP	SALT
JISLUT	ANHYDRATE
JISWOY	ANHYDRATE

WEWWAW	ANHYDRATE
WEXLIU	SOLVATE
WEXWOL	ANHYDRATE
WICSUW	ANHYDRATE
WIFZUI	SALT HYDRATE
WIJNOS	SALT
WIKDEZ	ANHYDRATE
WIKVIV	ANHYDRATE
WIMBAV	ANHYDRATE
WIMFON	SALT HYDRATE
WIMSOA	ANHYDRATE
WIMZOH	ANHYDRATE
WINBAW	ANHYDRATE
WINJAF	ANHYDRATE
WINVAR*	ANHYDRATE
WINWUL	ANHYDRATE
WIPKUB	ANHYDRATE
WIRXAW	ANHYDRATE
WITDEK	ANHYDRATE
WIZFEQ	ANHYDRATE
WIZHUJ	ANHYDRATE
WOBHUR*	ANHYDRATE
WOBMUV	SALT
WOBQEK	COCRYSTAL
WOBWIT	ANHYDRATE
WOCJED	ANHYDRATE
WOFHIK	ANHYDRATE
WOGRIT	ANHYDRATE
WOGVEV	ANHYDRATE
WOKLUF	SALT HYDRATE
WOKPER*	ANHYDRATE
WOLXOK	ANHYDRATE
WOLYUR	SALT
WOLZAY	SALT
WONVEA	SALT HYDRATE
WOQBAF	COCRYSTAL

JITNOP	SALT
JIVXES	SALT
JIWPEL	ANHYDRATE
JIWPOV	ANHYDRATE
JIWQAI	SALT
JOFWIM	ANHYDRATE
JOHGEU	ANHYDRATE
JOHJIB	ANHYDRATE
JOKWEM	ANHYDRATE
JOLCOE	SOLVATE
JOPJAZ	ANHYDRATE
JOQVAO	SALT HYDRATE
JORGOO	SALT
JOTNAH	ANHYDRATE
JOWCAB	ANHYDRATE
JOWGAF	SALT
JOWNAM	ANHYDRATE
JOWPAO	ANHYDRATE
JOWXUO	ANHYDRATE
JOWZIG	COCRYSTAL
JOYHEK	ANHYDRATE
JOYJAK	COCRYSTAL
JUBFOB	ANHYDRATE
JUBFUH	ANHYDRATE
JUDRAB	ANHYDRATE
JUDRIJ	ANHYDRATE
JUGVUE	ANHYDRATE
JUHGAU	SALT
JUHLUT	SALT
JUHNUV	ANHYDRATE
JUKMEJ	SALT HYDRATE
JULBUN	ANHYDRATE
JULHED	ANHYDRATE
JULWUI	SALT
JULZAR	ANHYDRATE
JUSBUU	ANHYDRATE

WORWAD	ANHYDRATE
WOSJIZ	SALT
WOTJOE	ANHYDRATE
WOTZAG	COCRYSTAL
WOVGIZ	ANHYDRATE
WOVYOV	SALT
WUCGUW	ANHYDRATE
WUKGAK	COCRYSTAL
WUKKOC	ANHYDRATE
WULZIM	ANHYDRATE
WUMKUK	ANHYDRATE
WUNYAH	ANHYDRATE
WUQGAQ	ANHYDRATE
WURMOM	ANHYDRATE
WUTCEU	ANHYDRATE
WUTSIO	ANHYDRATE
WUVKEE	COCRYSTAL
WUVYUH*	ANHYDRATE
WUWTIR	SALT
WUWTOX	SALT
WUYPOW	ANHYDRATE
WUZHOP	COCRYSTAL
XABREY	ANHYDRATE
XADXOR	SALT
XAJPII	ANHYDRATE
XAQXOE	ANHYDRATE
XAQYEV	SALT HYDRATE
XARHII	ANHYDRATE
XAVPAM	ANHYDRATE
XAVTOF	SALT
XAVVIB*	ANHYDRATE
XAXCOQ	ANHYDRATE
XAYBOO	ANHYDRATE
XAYGIP	SALT
XAYGUB	SALT
XAZCEH	ANHYDRATE

JUZBUB	ANHYDRATE
KACRUC	ANHYDRATE
KADFAY	ANHYDRATE
KADNEI	ANHYDRATE
KADPOU	ANHYDRATE
KADTEO	ANHYDRATE
KAHLEK	ANHYDRATE
KAHRIW	SALT HYDRATE
KAHTUK	ANHYDRATE
KAHWEX	SALT
KAJQOB	ANHYDRATE
KAJROD	ANHYDRATE
KAMCOQ	ANHYDRATE
KAMHAJ	ANHYDRATE
KANYUU	ANHYDRATE
KAPVIH	ANHYDRATE
KAQGAM	SALT
KARCOW	ANHYDRATE
KARGUF	SALT
KASKUL	ANHYDRATE
KASSOM	ANHYDRATE
KASXUY	SALT
KAWFAQ	ANHYDRATE
KAWJAU	ANHYDRATE
KAXGEW	ANHYDRATE
KAXHAS	ANHYDRATE
KAXWAJ	COCRYSTAL
KAXXAI	ANHYDRATE
KAYVAH	ANHYDRATE
KAYWIR	ANHYDRATE
KAZDUL	ANHYDRATE
KDGLUM	SALT HYDRATE
KEBGAB	SALT
KEBHIK	ANHYDRATE
KEBWUK	ANHYDRATE
KECYBU	ANHYDRATE

XAZVOK	SALT
XAZWIF	ANHYDRATE
XAZYED	ANHYDRATE
XEBQOL	SOLVATE
XEDLIC	ANHYDRATE
XEFJOI	SOLVATE
XEHHEX	ANHYDRATE
XEJLII	SALT
XEJNUV	ANHYDRATE
XELLEF	ANHYDRATE
XELLOP	ANHYDRATE
XENXUL	SALT HYDRATE
XEPXOF	ANHYDRATE
XEQGUX	SOLVATE
XERBAX	SALT
XESKEM	SALT COCRYSTAL
XESSUJ	ANHYDRATE
XETKOX	ANHYDRATE
XETZIG	COCRYSTAL
XEWMER	ANHYDRATE
XEWMOB	ANHYDRATE
XEWZAB	ANHYDRATE
XEZGAK	ANHYDRATE
XEZGEP	SALT
XIFRUZ	ANHYDRATE
XIHFAW	SALT
XIJKIK	SALT
XILPUD	ANHYDRATE
XILQOY	ANHYDRATE
XIMBZB	SALT
XIMMEL	ANHYDRATE
XINBEB	SALT
XINRUH	SALT
XIPYIF	SALT
XISLIV	SALT
XIYXIN	ANHYDRATE

KEDDAY	ANHYDRATE
KEFYOK	ANHYDRATE
KEGTAS	ANHYDRATE
KEKVEC	SALT
KELFOX	ANHYDRATE
KELGEO	ANHYDRATE
KELNIA	ANHYDRATE
KEMBUA	ANHYDRATE
KEMDEM	ANHYDRATE
KERFOC	ANHYDRATE
KERNAW	ANHYDRATE
KERNEA	SALT
KESNEC*	ANHYDRATE
KESYAJ	ANHYDRATE
KETDIW	SALT HYDRATE
KETDUI	ANHYDRATE
KETJUP	SALT
KETTUY	ANHYDRATE
KETVEK	ANHYDRATE
KETYAJ	ANHYDRATE
KEVMEF	ANHYDRATE
KEWQAG	ANHYDRATE
KEWYES	ANHYDRATE
KEXYOC	ANHYDRATE
KHTRON	SALT
KIBPEQ	SALT
KIDJUD	ANHYDRATE
KIDQIZ	ANHYDRATE
KIFHUC	ANHYDRATE
KIFLEQ	SALT
KIFMOC	ANHYDRATE
KIFPUL	SALT
KIGKER	COCRYSTAL
KIGTOL	ANHYDRATE
KIHYOQ	COCRYSTAL
KIJGUF	ANHYDRATE

XIZPEB	SALT HYDRATE
XNAPAC	ANHYDRATE
XOCFOK	ANHYDRATE
XOCJEE	ANHYDRATE
XOCXUI	ANHYDRATE
XODCEZ	SOLVATE
XOGDIH	ANHYDRATE
XOGDUT*	ANHYDRATE
XOHYIE	ANHYDRATE
XOJMIT	ANHYDRATE
XOJXOL	ANHYDRATE
XOKXEC	SALT HYDRATE
XOKYAZ	ANHYDRATE
XOKZAA	ANHYDRATE
XOLCUX	SALT COCRYSTAL
XOLHUC	COCRYSTAL
XOLJOZ	SALT
XOLQUM	ANHYDRATE
XOMTUP	SALT
XOPWEF	ANHYDRATE
XORLIZ	COCRYSTAL
XOSBIR	ANHYDRATE
XOSGUH	ANHYDRATE
XOTQED	ANHYDRATE
XOWLIG	ANHYDRATE
XOYQIN	ANHYDRATE
XOYVOX	ANHYDRATE
XOZBUK	ANHYDRATE
XUCKOX	SOLVATE
XUDWUO	ANHYDRATE
XUDXAV	ANHYDRATE
XUDXID	ANHYDRATE
XUFDUY	ANHYDRATE
XUJCIO	ANHYDRATE
XULDUD	ANHYDRATE
XULWAE	ANHYDRATE

KIJQEA	ANHYDRATE
KIJSIH	COCRYSTAL
KIMLEX	SALT
KINQIJ	ANHYDRATE
KINSIL	ANHYDRATE
KIPTOU	SALT
KIRHEA	ANHYDRATE
KISQEJ	ANHYDRATE
KISQUZ	ANHYDRATE
KIVSEP	ANHYDRATE
KIYSIU	SOLVATE
KIZCEC	ANHYDRATE
KIZMAI	ANHYDRATE
KNATAR	SALT
KNTMET	SALT
KOJNAA	ANHYDRATE
KOJSUZ	ANHYDRATE
KOKJOL	ANHYDRATE
KOKQUW	ANHYDRATE
KOMDAR	ANHYDRATE
KOMZUJ*	ANHYDRATE
KONTIQ	HYDRATE
KOPDAW	ANHYDRATE
KOPJUW	COCRYSTAL
KOTDEC	ANHYDRATE
KOWYEA	ANHYDRATE
KOYMES	ANHYDRATE
KTCYQM	SALT
KUBNOK-D	SALT
KUBWAF	ANHYDRATE
KUCSAE	SALT
KUCYOY	ANHYDRATE
KUDFUM	SALT
KUDHIC	ANHYDRATE
KUDLIG	SOLVATE

XUNRON	ANHYDRATE
XUNTEH	SALT
XUPHEX	SALT
XUQSAD	SALT
XUTCAQ	SALT HYDRATE
XUTPOR	SALT
XUTPUY	ANHYDRATE
XUVLIJ	ANHYDRATE
XUVWUH	SALT HYDRATE
XUYHOO	ANHYDRATE
XUZBUQ	SALT SOLVATE
XYANAC	ANHYDRATE
YABHAM	COCRYSTAL
YABHEP	ANHYDRATE
YABKUI	ANHYDRATE
YACREZ	ANHYDRATE
YACTEC	ANHYDRATE
YADKEV	ANHYDRATE
YADKUL	ANHYDRATE
YAMHID	ANHYDRATE
YANMUW	SALT
YAPNEK	ANHYDRATE
YAQMAF	ANHYDRATE
YAQMIN	ANHYDRATE
YAQMUZ	ANHYDRATE
YAQNEK	ANHYDRATE
YARGON	ANHYDRATE
YARYAR	ANHYDRATE
YARZUN	ANHYDRATE
YASGOQ	COCRYSTAL
YASKOS	ANHYDRATE
YAVRIW	SALT
YAWPER	ANHYDRATE
YAWWIC	ANHYDRATE
YAXCEF	COCRYSTAL HYDRATE

KUDXAK	ANHYDRATE
KUFLUU	ANHYDRATE
KUGGIC	COCRYSTAL
KUGRIO	ANHYDRATE
KUGZAP	ANHYDRATE
KULSOA	SALT
KUMCAX	SALT
KUMCEB	SALT
KUNVEU	ANHYDRATE
KUPMUE	ANHYDRATE
KUQLEO	ANHYDRATE
KUQRUK	SALT HYDRATE
KURBOP*	ANHYDRATE
KUSVAW	SALT
KUSVEZ	ANHYDRATE
KUSVID	ANHYDRATE
KUSWUR	ANHYDRATE
KUTLIW	ANHYDRATE
KUTMAP	ANHYDRATE
KUTQIB	SALT
KUVWON	ANHYDRATE
KUVZAE	ANHYDRATE
KUWHOA	SALT
KUWPUP	COCRYSTAL
KUXJUI	ANHYDRATE
LABFID	SALT HYDRATE
LABHAX	ANHYDRATE
LABJON	ANHYDRATE
LABSEM-D	SALT
LABSEM-H	SALT
LAFKAE	ANHYDRATE
LAGWOH	ANHYDRATE
LAJZED	ANHYDRATE
LAKWOL	SALT
LALWIF	HYDRATE
LAMYAA	SALT

YAXCEH	ANHYDRATE
YAXDUW	ANHYDRATE
YAXWAX	ANHYDRATE
YEBQED	ANHYDRATE
YEBWIM	ANHYDRATE
YEFGOG	ANHYDRATE
YEFNEE	ANHYDRATE
YEGJID	ANHYDRATE
YEHPOQ	ANHYDRATE
YEJJUU	SALT HYDRATE
YEJLII	ANHYDRATE
YEJNAC	ANHYDRATE
YEJRUA	ANHYDRATE
YELHUS	ANHYDRATE
YEMZAT-D	ANHYDRATE
YENZUN	ANHYDRATE
YEPCAY	SALT
YEPHAC	ANHYDRATE
YEPRAN	ANHYDRATE
YERLAJ	ANHYDRATE
YERRUI	ANHYDRATE
YICDAR	SALT SOLVATE
YICDIZ	ANHYDRATE
YICDUL	ANHYDRATE
YICFEX	ANHYDRATE
YICJAX	SALT
YICJIF	SALT
YICNAZ	SALT HYDRATE
YIDLEC	ANHYDRATE
YIFTIS	SOLVATE
YIGPIO	ANHYDRATE
YIHJAA	SALT
YIHKAB	ANHYDRATE
YIJDOK	ANHYDRATE
YIKNEN	SOLVATE
YILQIV	ANHYDRATE

LANXOO	ANHYDRATE
LARGIN	SALT
LASBUC	SOLVATE
LATNEB	SOLVATE
LATSEG*	COCRYSTAL
LATTAD	COCRYSTAL
LATTOR	COCRYSTAL
LAURAC	ANHYDRATE
LAVMOK	ANHYDRATE
LAVYOX	SALT HYDRATE
LAWYOY	ANHYDRATE
LAXJOK	ANHYDRATE
LAZHID	SALT
LAZPAD	SALT
LAZRAF	ANHYDRATE
LCYSTI	ANHYDRATE
LCYSTN	ANHYDRATE
LEBCIE	ANHYDRATE
LEBGEE	ANHYDRATE
LEBKIN	ANHYDRATE
LECGUV	ANHYDRATE
LECROB	SOLVATE
LEDQEQ	ANHYDRATE
LEHWAX	ANHYDRATE
LEJMOE	COCRYSTAL
LEJXON	COCRYSTAL
LENBOV	ANHYDRATE
LEPMEZ	ANHYDRATE
LEPPIF	ANHYDRATE
LEPPUS	ANHYDRATE
LEPRON	ANHYDRATE
LEPTER	ANHYDRATE
LEQKEY	ANHYDRATE
LEQPIH	ANHYDRATE
LEQRIK	ANHYDRATE
LESFOF	ANHYDRATE

YINSAR	ANHYDRATE
YIPGEL	SALT
YIPGUB	ANHYDRATE
YIQJEN	ANHYDRATE
YIRVAW	SOLVATE
YIRXAZ	ANHYDRATE
YISBIM	ANHYDRATE
YIVGIV	SALT
YIVRIF	ANHYDRATE
YIVROL	ANHYDRATE
YIVRUR	ANHYDRATE
YIWTON	SALT
YIXTAB	ANHYDRATE
YIXTEF	ANHYDRATE
YIXVAD	ANHYDRATE
YIXZOW	SALT HYDRATE
YODFAZ	ANHYDRATE
YOFNAK	ANHYDRATE
YOHCON	ANHYDRATE
YOHKOX	SALT HYDRATE
YOJFEK	SALT
YOKTAT	SALT
YOLDAF	ANHYDRATE
YOLVUQ	ANHYDRATE
YOLYII	SALT
YOLYOO	HYDRATE
YOMXUT	ANHYDRATE
YORYAH	ANHYDRATE
YOTJUN	ANHYDRATE
YOVCOD	ANHYDRATE
YOWXAK	ANHYDRATE
YOWYOY	ANHYDRATE
YOYFIB	SOLVATE
YOZJUU	ANHYDRATE
YOZSEL	ANHYDRATE
YOZZAQ	ANHYDRATE

LESHOI	ANHYDRATE
LETGIA	SALT
LEUCIN*	ANHYDRATE
LEVNAC	SOLVATE
LEVNUW	ANHYDRATE
LEXVEP	ANHYDRATE
LEYXEU	ANHYDRATE
LEZJAB	ANHYDRATE
LEZPUC	SOLVATE
LEZZAS	SALT COCRYSTAL
LGLUAC	ANHYDRATE
LHISTD	ANHYDRATE
LHLASP	SALT HYDRATE
LIACET	SALT HYDRATE
LIBRUL	HYDRATE
LICDIM	ANHYDRATE
LICMER	ANHYDRATE
LICMIT	ANHYDRATE
LICPOE	SALT
LICRAS	ANHYDRATE
LIFFEM	SALT HYDRATE
LIGPUM	ANHYDRATE
LIGVUU	ANHYDRATE
LIGXUU	ANHYDRATE
LIHGAM	SALT
LIHXUW	ANHYDRATE
LIJYAE	ANHYDRATE
LIKBOW	ANHYDRATE
LIKCEN	ANHYDRATE
LIKGAN	ANHYDRATE
LIKGUI	ANHYDRATE
LILHAP	SALT
LILJUL	ANHYDRATE
LILRIJ	ANHYDRATE
LILSUU	ANHYDRATE
LILZOV	ANHYDRATE

YUBWAV	ANHYDRATE
YUCCIJ	ANHYDRATE
YUCSIZ	ANHYDRATE
YUFGAJ	COCRYSTAL
YUFVOL	ANHYDRATE
YUHGOX	ANHYDRATE
YUHHUE*	ANHYDRATE
YUJGIT	ANHYDRATE
YUJJOE	COCRYSTAL
YUKGUG	ANHYDRATE
YUKPAW	SALT
YULNUO	ANHYDRATE
YUMHOE	ANHYDRATE
YUWZEW	SALT
YUXCAV	SALT
YUXFIH	SALT
YUYHIJ	ANHYDRATE
YUYPAJ	ANHYDRATE
ZAJGUM	HYDRATE
ZAKMIH	ANHYDRATE
ZAPFED	ANHYDRATE
ZAQZUM	ANHYDRATE
ZAVVUN	ANHYDRATE
ZAXKUE	ANHYDRATE
ZAYKOZ	HYDRATE
ZEBVEJ	ANHYDRATE
ZEBWEK	ANHYDRATE
ZECZIQ	ANHYDRATE
ZEDPIH	ANHYDRATE
ZEDPON	SALT
ZEFFEX	SALT
ZEGSUZ	ANHYDRATE
ZEKPOW	ANHYDRATE
ZELDOJ	ANHYDRATE
ZEMXAQ	COCRYSTAL
ZENQDX	ANHYDRATE

LIMNIE	ANHYDRATE
LINMIF	ANHYDRATE
LIPBIW	ANHYDRATE
LIQQIN	COCRYSTAL
LIRRAG	ANHYDRATE
LISDOH	ANHYDRATE
LITLEG	ANHYDRATE
LIVNOV	SALT
LIVSAL	ANHYDRATE
LIVVIW	SALT
LIWRAK	ANHYDRATE
LIYTIW	ANHYDRATE
LOCVOO	COCRYSTAL
LOFKAS	ANHYDRATE
LOFKIB	COCRYSTAL
LOHKOK	SALT
LOHTAE*	ANHYDRATE
LOHVEK	ANHYDRATE
LOLYUG	ANHYDRATE
LOPQEO*	ANHYDRATE
LOQDIG	SOLVATE
LOQLAE	ANHYDRATE
LOQNAI	SALT
LOQQEN	ANHYDRATE
LORVAP	ANHYDRATE
LORWUL	ANHYDRATE
LOTLIR	SALT COCRYSTAL
LOTLUD	SALT
LOTZEZ	ANHYDRATE
LOXHAJ	COCRYSTAL
LOZDUZ	ANHYDRATE
LOZYII*	ANHYDRATE
LPYGLU	ANHYDRATE
LSERIN	ANHYDRATE
LSERMH-D	HYDRATE

ZENVAP	ANHYDRATE
ZEPDAZ	ANHYDRATE
ZEPFAB	ANHYDRATE
ZETSEW	ANHYDRATE
ZETZOP	ANHYDRATE
ZEXHIV	SALT
ZEXJAP	SALT
ZEXQUO	SALT
ZEXREZ	ANHYDRATE
ZEXRIF	ANHYDRATE
ZEXXOP	ANHYDRATE
ZEYBIO	ANHYDRATE
ZEYDEO	SALT
ZEYHUG	ANHYDRATE
ZIGPAG	COCRYSTAL
ZILHOR	ANHYDRATE
ZILNOX	ANHYDRATE
ZIMHIO	SOLVATE
ZIVGEQ	ANHYDRATE
ZIWVIK	ANHYDRATE
ZIYWAH	ANHYDRATE
ZOBBEZ	COCRYSTAL
ZOCPUE	ANHYDRATE
ZODJEH	ANHYDRATE
ZODWIY	COCRYSTAL
ZOGQAN	ANHYDRATE
ZOKHAK	ANHYDRATE
ZOPTED	ANHYDRATE
ZOQZIQ	ANHYDRATE
ZOSLAU	ANHYDRATE
ZOWSUZ	ANHYDRATE
ZOXHAX	ANHYDRATE
ZOYLII	ANHYDRATE
ZOYMOP	ANHYDRATE
ZUDTAV	COCRYSTAL SOLVATE

LSERMH-H	HYDRATE
LUBPOP	ANHYDRATE
LUFCIY	ANHYDRATE
LUKXAQ	ANHYDRATE
LULKEJ	ANHYDRATE
LULLOT	ANHYDRATE
LUMHOR	ANHYDRATE
LUMXAS	ANHYDRATE
LUNPOZ	ANHYDRATE
LUNRUI	SALT
LUPGIN	ANHYDRATE
LUPGUY	ANHYDRATE
LUPVOH	ANHYDRATE
LUQLIU	ANHYDRATE
LUQVIC	ANHYDRATE
LURHAJ	ANHYDRATE
LUSKUF*	ANHYDRATE
LUTDUR	SOLVATE
LUTPEV	ANHYDRATE
LUVYUX	ANHYDRATE
LUXXAD	ANHYDRATE
LUYMOH	SALT HYDRATE
LYSDOL	ANHYDRATE
MABZNA	ANHYDRATE
MACCID	COCRYSTAL
MACPRP	SALT
MACZIB	ANHYDRATE
MAFPOA	ANHYDRATE
MAGTUK	SALT
MAKDEH	ANHYDRATE
MALEHY	ANHYDRATE
MALIAC	ANHYDRATE
MALNAC	ANHYDRATE
MALOAM	ANHYDRATE
MALONT	ANHYDRATE
MALSOJ	ANHYDRATE

ZUHGOY	ANHYDRATE
ZUHKAO	ANHYDRATE
ZUHQAU	HYDRATE
ZUHRID	ANHYDRATE
ZUHTOL	ANHYDRATE
ZUHXAB	ANHYDRATE
ZULGEU	ANHYDRATE
ZUMPUS	ANHYDRATE
ZUMXIQ	ANHYDRATE
ZUNHOH	HYDRATE
ZURMEG	ANHYDRATE
ZUWTOA	ANHYDRATE
ZZZAUS	ANHYDRATE
ZZZAVM	ANHYDRATE
ZZZAWJ	ANHYDRATE
ZZZBCS	ANHYDRATE
ZZZBLP	ANHYDRATE
ZZZDDJ	ANHYDRATE
ZZZEEU	ANHYDRATE
ZZZFEE	ANHYDRATE
ZZZGMW	SOLVATE
ZZZHQU	ANHYDRATE
ZZZHWI	ANHYDRATE
ZZZIYE	ANHYDRATE
ZZZIZM	SALT
ZZZJIQ	ANHYDRATE
ZZZKPE	ANHYDRATE
ZZZLBS	SALT
ZZZLGU	ANHYDRATE
ZZZMGS	ANHYDRATE
ZZZMMG	ANHYDRATE
ZZZMRW	ANHYDRATE
ZZZMUC	ANHYDRATE
ZZZNQS	ANHYDRATE
ZZZNUK	ANHYDRATE
ZZZODU	ANHYDRATE

MAMGUD	ANHYDRATE
MAMPUM	SALT
MANMUJ	SALT
MANPIM	ANHYDRATE
MANSTB	ANHYDRATE
MAPLIZ	ANHYDRATE
MAPMIP	SALT
MAPPHI	SALT
MAQWIM	ANHYDRATE
MARBEM	HYDRATE
MATDEQ	ANHYDRATE
MAVGEW	ANHYDRATE
MAXDEV	SALT SOLVATE COCRYSTAL
MAXKEB	SALT
MAXKUT	SALT
MAZNIM	ANHYDRATE
MAZXOB	SALT
MBLARA	ANHYDRATE
MBPHOL	ANHYDRATE
MBRMAD	ANHYDRATE

ZZZPNG	ANHYDRATE
ZZZPRC	ANHYDRATE
ZZZPRO	ANHYDRATE
ZZZPUS	ANHYDRATE
ZZZQNK	ANHYDRATE
ZZZSBA	SALT HYDRATE
ZZZSSY	HYDRATE
ZZZTTC	ANHYDRATE
ZZZUOQ	SALT HYDRATE
ZZZUXA	ANHYDRATE
ZZZUYA	SALT
ZZZVBQ	SOLVATE
ZZZVCO	ANHYDRATE
ZZZVTY	ANHYDRATE
ZZZVUY	ANHYDRATE
ZZZVXQ	ANHYDRATE
ZZZWGK*	ANHYDRATE
ZZZWNG	ANHYDRATE
ZZZZCA	ANHYDRATE
ZZZZCB	ANHYDRATE

S5. Tables of Refcodes Not Included as Polymorphs

Entries in Table S5 are not included in the overall list of polymorphs. The 60 entries in blue in Table S3 that were determined to be polymorphic compounds are not included in Table S5, and were instead integrated into Table S4 and highlighted with an asterisk. Table S4 only includes compounds that have two structurally characterized entries in a refcode which contain 3D coordinates. Several entries in Table S5 have two forms listed as polymorphs, but do not have multiple forms with 3D coordinates known, and therefore are not included in Table S4. In this table and below, PT means phase transition.

Table S5 Description of compounds with only one entry in the polymorph list (shown as blue in Table S3) explaining why they are not included in Table S4 (857 refcodes).

Refcode	Crystal Type	More entries in CSD?	Multiple Polymorphs?	Explanation for why to not include
ABOPUC	anhydrate	no		
ACADIR	salt	yes	yes	Both listed as poly, one without 3D coord.
ACENYL	anhydrate	yes	yes, PT	Updated late 2015 or 2016
ACESAE	cocrystal	no		
ACETEJ	cocrystal	no		
ACIKAZ	solvate	no		
ACONEN	solvate	no		
ACOVAQ	anhydrate	no		
ACOVIY	salt	no		
ACOVOE	salt	no		
ACTDGU	cocrystal hydrate	yes	yes	Both listed as poly, one without 3D coord.
AFULIY	salt	no		
AHBONM	anhydrate	no		
AHBPRG	anhydrate	yes	yes	Both listed as poly, one without 3D coord.
AIPBAR	anhydrate	no		
AJUVAC	cocrystal hydrate	no		
AJUVEG	cocrystal hydrate	no		
ALDCAR	anhydrate	yes	yes	
AMEPOS	anhydrate	yes	no	
ANISUC	anhydrate	yes	yes	Both listed as poly, both added in 2016 and one without 3D coord.
APAPAD	hydrate	yes	same unit cell	Both listed as poly, one without 3D coord.

APUYOA	solvate	yes	no	
AQOZUB	anhydrate	yes	no	Both listed as poly, one without 3D coord.
ARSMET	anhydrate	no		
ASATOD	solvate	no		
AZOXHD	anhydrate	yes	yes	Both listed as poly, one without 3D coord.
AZSTBD	anhydrate	yes	yes	Both listed as poly, one without 3D coord.
BACLCH	anhydrate	no		
BAHOXL	salt	no		
BANYIY	anhydrate	no		
BATCAA	anhydrate	yes	yes	Both listed as poly, one without 3D coord.
BEAVBA	salt cocrystal solvate	no		
BECJEY	anhydrate	yes	yes	All listed as poly, only one with 3D coord.
BECWOV	anhydrate	yes	yes	Both listed as poly, one without 3D coord.
BEFVAJ	anhydrate	yes	no	
BEMBEB	anhydrate	yes	no	
BEOACT	anhydrate	yes	yes	All listed as poly, only one with 3D coord.
BEWFOY	salt hydrate	no		
BEWPEY	anhydrate	no		
BEYWAF	salt	no		
BEYWEJ	salt	no		
BEYWIN	salt	no		
BIBYUG	salt	yes	PT	All listed as poly, only one with 3D coord.
BICVUE	solvate	yes	yes	Both listed as poly, one without 3D coord.
BIKHIN	solvate	yes	yes	Both listed as poly, one without 3D coord.
BILNOZ	anhydrate	yes	yes	All listed as poly, only one with 3D coord.
BMLHYD	solvate	yes	PT	Both listed as poly, one without 3D coord.
BOBVEU	anhydrate	no		
BOKDEM	anhydrate	no		
BOKLAO	anhydrate	yes	yes	Both listed as poly, one without 3D coord.
BOPSIJ	salt	no		
BOSJUP	anhydrate	no		
BOTCUI	anhydrate	yes	yes	Both listed as poly, one without 3D coord.
BPXBTZ	anhydrate	yes	yes	Both listed as poly, one without 3D coord.
BRASAC	anhydrate	yes	no	Both listed as poly, one without 3D coord.
BRNAPT	anhydrate	no		
BRNICH	anhydrate	yes	yes	Both listed as poly, one without 3D coord.
BROXUR	anhydrate	yes	yes	Two listed as poly, two with 3D coord. are same form

BRUDAG	anhydrate	no		
BUCXEC	salt	yes	PT	All listed as poly, only one with 3D coord. Similar unit cells, temp difference
BUJMUO	anhydrate	no		
BULMEA	salt hydrate	yes	no	Two are deuterated. Only 1 with H missing 3D coord., only one D listed as poly. Text says PT in deuterated
BUNDAC	anhydrate	yes	yes	All listed as poly, only one with 3D coord.
BUNDEC	anhydrate	no		
BUNSIM	anhydrate	yes	PT	All listed as poly, only one with 3D coord.
BUTCLT	anhydrate	yes	PT	All listed as poly, only one with 3D coord.
BUYROC	anhydrate	yes	yes	All listed as poly, only one with 3D coord.
BZCPTI	anhydrate	yes	yes	All listed as poly, only one with 3D coord.
BZOFOX	anhydrate	yes	yes	All listed as poly, only one with 3D coord.
CACCIS	salt cocrystal	yes	yes	Both listed as poly, one without 3D coord.
CAHEXT	salt cocrystal hydrate	yes	yes	Both listed as poly, one without 3D coord.
CANTRQ	anhydrate	yes	maybe	All listed as poly, only one with 3D coord.
CANYOG	salt	no		
CAPTAZ	salt	no		
CBZYTO	anhydrate	yes	yes	Both listed as poly, one without 3D coord.
CEDXUE	anhydrate	yes	yes	Both listed as poly, one without 3D coord.
CEJWUJ	anhydrate	no		
CELQUH	anhydrate	no		
CELRIW	anhydrate	yes	yes	Both have 3D coord., one added in 2016
CESCNM	salt	yes	yes	All listed as poly, only one with 3D coord.
CHNOCH	anhydrate	yes	yes	Both listed as poly, one without 3D coord.
CHOLES	hydrate	yes	yes	All listed as poly, only one with 3D coord.
CIRDIS	salt hydrate	no		
CITROM	anhydrate	yes	yes	All listed as poly, only one with 3D coord.
CIZGUN	salt hydrate	yes	yes	Both listed as poly, one without 3D coord.
CLMCBU	anhydrate	yes	yes	Both listed as poly, one without 3D coord.
CLNPHT	anhydrate	yes	PT	All listed as poly, only one with 3D coord.
CLPMCY	anhydrate	yes	yes	Two listed as poly, only one with 3D coord.
CMHXDC	anhydrate	yes	yes	Both listed as poly, one without 3D coord.
COBJIM	anhydrate	yes	PT	Both listed as poly, one without 3D coord.
COCGEG	solvate	yes	yes	Both listed as poly, one without 3D coord.
COCGOQ	solvate hydrate	yes	yes	Both listed as poly, one without 3D coord.
COHZAA	salt	no		
COQRUV	anhydrate	no		

CORSIL	anhydrate	yes	yes	Both listed as poly, one without 3D coord.
CORTEI	anhydrate	yes	yes	Both listed as poly, one without 3D coord.
COTXUG	anhydrate	no		
COVZOE	salt	no		
CPPHEN	anhydrate	yes	yes	All listed as poly, only one with 3D coord.
CTCYME	anhydrate	no		
CUVTAO	anhydrate	yes	yes	Both listed as poly, one without 3D coord.
CUZKOX	salt	yes	yes	Both listed as poly, one without 3D coord.
CYCLAM	salt	yes	yes	Both listed as poly, one without 3D coord.
CYPRCA	anhydrate	yes	yes	Both listed as poly, one without 3D coord.
CYTDEC	anhydrate	yes	PT	Both listed as poly, one without 3D coord.
DABGUI	anhydrate	no		
DABHAP	hydrate	no		
DACTDO	anhydrate	yes	yes	All listed as poly, only one with 3D coord.
DASMEP	anhydrate	yes	yes	Both listed as poly, one without 3D coord.
DATNBZ	anhydrate	yes	no	
DAWSOJ	salt	yes	PT	Both listed as poly, one without 3D coord.
DAWVOM	salt	yes	PT	All listed as poly, only one with 3D coord.
DAXMAS	anhydrate	no		
DAZCLA	anhydrate	yes	yes	Both listed as poly, one without 3D coord.
DBPOLO	anhydrate	no		
DCAETO	solvate hydrate	yes	yes	Both listed as poly, one without 3D coord.
DCLPHM	anhydrate	yes	no	All have 3D coord., only one listed as poly.
DEBMUS	anhydrate	no		
DEBNAZ	anhydrate	no		
DEDBUJ	hydrate	no		
DEHSIS	salt	yes	no	Both listed as poly, one without 3D coord.
DEKFUU	anhydrate	yes	yes	Both listed as poly, one without 3D coord.
DESYIJ	anhydrate	no		
DEYNAP	anhydrate	yes	yes	All listed as poly, only one with 3D coord.
DILKEO	anhydrate	yes	PT	All listed as poly, only one with 3D coord.
DMBMCL	anhydrate	yes	yes	Both listed as poly, one without 3D coord.
DMEWAR	anhydrate	yes	yes	Both listed as poly, one without 3D coord.
DMHXDM	anhydrate	no		
DMPOXP	salt	yes	no	All listed as poly, only one with 3D coord.
DMTRYP	anhydrate	yes	yes	Both listed as poly, one without 3D coord.
DOBCDC	anhydrate	yes	yes	Both listed as poly, one without 3D coord.
DOBRUH	anhydrate	yes	yes	Both listed as poly, one without 3D coord.

DOHQAS	anhydrate	no		
DOHQEW	anhydrate	no		
DOMZIQ	anhydrate	no		
DOWWET	salt	no		
DOWWIX	salt	no		
DOWWOD	salt	no		
DOWWUJ	salt	no		
DOWXAQ	salt	no		
DOWXEU	salt	no		
DOWXIY	salt	no		
DOWXOE	salt	no		
DOWXUK	salt	no		
DOWYAR	salt	no		
DOWYEV	salt	no		
DOWYIZ	salt	no		
DOWYOF	salt	no		
DOWYUL	salt	no		
DOWZAS	salt	no		
DOWZEW	salt	no		
DOWZIA	salt	no		
DOWZOG	salt	no		
DOWZUM	salt	no		
DOXDIS	anhydrate	yes	yes	Both listed as poly, one without 3D coord.
DPHNOL	anhydrate	yes	yes	All listed as poly, only one with 3D coord.
DPOCTT	anhydrate	yes	yes	Two have 3D coord. but those are same and one wasn't listed as poly.
DPRTMA	salt	yes	yes	Both listed as poly, one without 3D coord.
DPTOTE	anhydrate	yes	yes	Both listed as poly, one without 3D coord.
DTACAC	anhydrate	no		
DTHDOX	anhydrate	yes	no	Only one listed as poly, both have 3D coord.
DTHDSX	anhydrate	no		
DTHTPP	anhydrate	yes	yes	All listed as poly, only one with 3D coord.
DUNGID	anhydrate	no		
DUXSAR	cocrystal	no		
DUXSEV	salt solvate	no		
EBEWOX	anhydrate	no		
ECITOA	anhydrate	no		
ECITUG	anhydrate	no		
ECIVAO	anhydrate	no		

ECIVES	anhydrate	no		
ECIVIW	anhydrate	no		
ECUVED	cocrystal	yes	PT	Both listed as poly, one without 3D coord.
EDAPOQ	cocrystal	no		
EFICOL	salt	no		
EFIKOT	anhydrate	yes	PT	Both listed as poly, one without 3D coord.
EFINAJ	salt	no		
EFINEN	salt	no		
EFINIR	salt	no		
EHEREO	anhydrate	no		
EMCORR	anhydrate	yes	yes	Both listed as poly, one without 3D coord.
EMONID	solvate	no		
ENICEK	cocrystal	no		
EPETCQ	salt	no		
EPZPHX	anhydrate	no		
ERGCAL	anhydrate	no		
ESTTRD	anhydrate	yes	yes	Two listed as poly, only one with 3D coord.
ETAMBR	salt	no		
ETBARB	anhydrate	no		
ETHANE	anhydrate	yes	PT	Only one with 3D coord.
ETHBME	solvate	yes	yes	Both listed as poly, one without 3D coord.
ETHLEN	anhydrate	yes		Only one with 3D coord.
EWAPIC	cocrystal	no		
EXCHLN	salt	no		
EXZPUN	anhydrate	yes	yes	All listed as poly, only one with 3D coord.
EYANRO	anhydrate	no		
EZELOK	anhydrate	yes	yes	Both listed as poly, one without 3D coord.
FABNUT	anhydrate	no		
FAJGUS	anhydrate	yes	PT	
FAJWAO	solvate	no		
FAKFUS	anhydrate	yes	yes	Both listed as poly, one without 3D coord.
FANDOO	anhydrate	no		
FAYFOA	anhydrate	no		
FAYFUG	anhydrate	no		
FAYVOQ	anhydrate	no		
FAYVUW	hydrate	no		
FECHOM	anhydrate	no		
FEFPAI	anhydrate	no		

FEHWAR	salt	no		
FENTEX	salt	no		
FEXHEW	anhydrate	no		
FEXHOH	cocrystal	no		
FEYGIZ	anhydrate	yes	yes	Both listed as poly, one without 3D coord.
FIFCEC	anhydrate	yes	yes	Both listed as poly, one without 3D coord.
FILYAA	solvate	no		
FIMDOU	salt	no		
FIMGIR	salt solvate	no		
FIMTUQ	salt	no		
FIMVAY	salt	no		
FINZIL	anhydrate	no		
FINZOR	anhydrate	no		
FIVGAS	salt	no		
FIYFAU	solvate	no		
FIYTIQ	salt	yes	no	Only one with 3D coord.
FIYTOW	salt	yes	no	Only one with 3D coord.
FIYTUC	salt	yes	no	Only one with 3D coord.
FIZQOW	anhydrate	no		
FIZVUF	salt hydrate	no		
FNAPTH	anhydrate	yes	no	All listed as poly, only one with 3D coord.
FOBCAB	salt	no		
FOJGAM	anhydrate	yes	yes	All listed as poly, only one with 3D coord.
FOJLUL	anhydrate	no		
FOJMAS	anhydrate	no		
FOJRAX	anhydrate	no		
FOMNAX	anhydrate	yes	yes	Both listed as poly, one without 3D coord.
FOVNOT	anhydrate	no		
FOWPIR	anhydrate	no		
FPYRMO	anhydrate	no		
FUFXOV	salt	no		
FUQMIO	salt hydrate	no		
FUQMOT	salt	yes	no	Both listed as poly, one without 3D coord.
FUQMUZ	salt	yes	no	Both listed as poly, one without 3D coord.
FURANE	anhydrate	yes	yes	Both listed as poly, one without 3D coord.
GADHAU	anhydrate	no		
GADVUD	cocrystal	no		
GAFLAA	anhydrate	no		

GAHLEG	anhydrate	yes	PT	All listed as poly, only one with 3D coord.
GAJDUQ	hydrate	no		
GAJFAY	hydrate solvate	no		
GAKGII	anhydrate	no		
GAKGIJ	anhydrate	no		
GAKWUK	cocrystal	no		
GAMJEJ	anhydrate	no		
GAXYEJ	salt	no		
GAXYIN	salt	no		
GAZFAO	salt solvate	no		
GAZFES	salt solvate	no		
GEBSOV	anhydrate	no		
GEBSOX	anhydrate	no		
GEFGII	salt	no		
GEGLOT	anhydrate	yes	yes	Both listed as poly, one without 3D coord.
GEHXEX	anhydrate	no		
GEJCAZ	anhydrate	no		
GEJCED	anhydrate	no		
GEJPOC	anhydrate	no		
GEJPUI	cocrystal	no		
GEJXAU	anhydrate	no		
GEKREV	anhydrate	no		
GELCIK	salt	no		
GERWEF	solvate	no		
GERWIJ	solvate	yes	no	
GEYRIL	anhydrate	no		
GEZSAH	anhydrate	no		
GIDLUB	cocrystal	no		
GIFSIX	anhydrate	no		
GIFSOD	anhydrate	no		
GIJTIC	anhydrate	yes	yes	Both listed as poly, one without 3D coord.
GIPGOB	salt	yes	no	
GIPGUH	salt	no		
GITWUC	anhydrate	no		
GOMFOD	anhydrate	yes	yes, PT	Both listed as poly, one without 3D coord.
GOQZUH	hydrate	no		
GORXOA	solvate	no		
GOVQAJ	cocrystal	no		

GOVQEN	solvate	no		
GOVQOX	solvate	no		
GOVRAK	cocrystal	no		
GOVREO	solvate	yes	no	
GUHDIW	anhydrate	no		
GUPICA	salt	yes	yes	Both listed as poly, one without 3D coord.
GUQPOZ	anhydrate	no		
GUTYAX	anhydrate	no		
GUVBIK	anhydrate	no		
GUXZUW	anhydrate	no		
GUYBAF	anhydrate	no		
GUYGOX	salt	yes	yes	All listed as poly, only one with 3D coord.
HAZGUK	salt	no		
HBTMDX	solvate	yes	yes	All listed as poly, only one with 3D coord.
HCCYHD	anhydrate	yes	yes	All listed as poly, only one with 3D coord.
HCLHEP	anhydrate	yes	yes	All listed as poly, only one with 3D coord.
HDBANT	anhydrate	yes	yes	All listed as poly, only one with 3D coord.
HEGHUX	anhydrate	no		
HEJTAR	anhydrate	yes	yes	Both listed as poly, one without 3D coord.
HELHEN	cocrystal	no		
HELHIR	cocrystal	no		
HEMLAM	anhydrate	no		
HEVMEA	salt	no		
HICDUS	anhydrate	no		
HICWOF	anhydrate	yes	yes	Both listed as poly, one without 3D coord.
HICWUL	anhydrate	yes	yes	Both listed as poly, one without 3D coord.
HICXIA	anhydrate	no		
HIDFEF	anhydrate	no		
HISREH	anhydrate	no		
HITSAG	anhydrate	no		
HIVLAA	anhydrate	yes	no	
HMPPYD	anhydrate	no		
HOLVOU	anhydrate	no		
HOLWOV	anhydrate	no		
HONQOQ	anhydrate	no		
HOTAUR	anhydrate	yes	no	
HOTZOF	salt solvate	no		
HOYVEX	anhydrate	no		

HOYVOH	anhydrate	no		
HOYWEY	anhydrate	no		
HUPWAR	cocrystal hydrate	no		
HUSQAN	anhydrate	yes	yes, PT	Both listed as poly, one without 3D coord.
HUSQIV	anhydrate	yes	yes, PT	Both listed as poly, one without 3D coord.
HUZBOT	salt hydrate	no		
HXMACA	anhydrate	yes	yes	Both listed as poly, one without 3D coord.
HXOXAM	anhydrate	yes	yes	All listed as poly, only one with 3D coord.
HXTACM	anhydrate	yes	yes	All listed as poly, only one with 3D coord.
IBRBPX	anhydrate	yes	yes	Both listed as poly, one without 3D coord.
IFOQAX	salt	no		
IKOBUF	anhydrate	no		
IMCYLP	anhydrate	yes	yes, PT	Both listed as poly, one without 3D coord.
IMEXUT	salt	no		
IMIYEI	salt cocrystal hydrate	no		
IMTCOS	anhydrate	no		
INDANT	anhydrate	no		
INIBUC	anhydrate	no		
ISENID	anhydrate	no		
ISENOJ	anhydrate	no		
ISENUP	anhydrate	no		
ISEPAX	anhydrate	no		
ISEPEB	anhydrate	yes	maybe	Both listed as poly, one without 3D coord.
ISUQOC	anhydrate	no		
ITUYAX	anhydrate	yes	yes	Both listed as poly, one without 3D coord.
IVOBOK	solvate	no		
IVURAS	anhydrate	no		
IWOCON	anhydrate	no		
JABNIJ	anhydrate	yes	no	
JABPEH	anhydrate	no		
JABPOR	anhydrate	no		
JAGJEG	salt	yes	yes	Both listed as poly, one without 3D coord.
JAGREP	anhydrate	yes	no	Both have 3D coord., only one listed as poly
JAKYOJ	anhydrate	yes	no	
JAPHAJ	salt	no		
JARXAB	anhydrate	no		
JAXGUK	anhydrate	no		

JEFDON	anhydrate	no		
JEFDUT	anhydrate	no		
JEHFIL	anhydrate	no		
JEMRYY	anhydrate	no		
JEPMIA	salt	no		
JEPMIB	salt	no		
JETKID	salt solvate	yes	yes	Both listed as poly, one without 3D coord.
JEVSOS	anhydrate	yes	yes	Both listed as poly, one without 3D coord.
JEXBIY	anhydrate	no		
JEXKUS	anhydrate	yes		No unit cell values in other two entries
JISFUM	anhydrate	yes	yes	All listed as poly, only one with 3D coord.
JUHJIF	solvate	yes	no	
JUVJAL	anhydrate	yes	no	
KADZEU	anhydrate	no		
KADZIY	anhydrate	no		
KAHHAC	salt	yes	yes	All listed as poly, only one with 3D coord.
KAHQOA	anhydrate	no		
KAHTEU	anhydrate	no		
KAKBAZ	salt	yes	yes	Both listed as poly, one without 3D coord.
KAWYOW	anhydrate	yes	yes	Both listed as poly, one without 3D coord.
KCAPRA	salt	yes	yes	Both listed as poly, one without 3D coord.
KCHLOR	salt	yes	yes	Both listed as poly, one without 3D coord.
KEPXAE	anhydrate	yes	yes	Both listed as poly, one without 3D coord.
KHACDC	salt	no		
KICTUN	anhydrate	no		
KIMKUM	salt	yes	yes, PT	Both listed as poly, one without 3D coord.
KOFKAR	anhydrate	yes	yes, PT	Both listed as poly, one without 3D coord.
KOFKEV	anhydrate	yes	yes, PT	Both listed as poly, one without 3D coord.
KOFKIZ	anhydrate	yes	yes, PT	Both listed as poly, one without 3D coord.
KONZEU	cocrystal	no		
KPALMA	salt	yes	yes	Both listed as poly, one without 3D coord.
KTTOXC	salt	no		
KUHPIM	solvate	no		
KUHPIR	anhydrate	no		
KUPPOA	salt	no		
LACTAK	anhydrate	no		
LADGAY	solvate	yes	yes	Both listed as poly, one without 3D coord.
LAYMED	anhydrate	yes	yes	Both listed as poly, one without 3D coord.

LEJDUZ	solvate	no		
LEJFAH	solvate	no		
LEJKIU	anhydrate	no		
LEJNET	cocrystal	no		
LEJNIX	cocrystal	no		
LEVCUK	anhydrate	yes	yes	All listed as poly, only one with 3D coord.
LEVWEP	salt	yes		
LICSOF	solvate	yes	no	
LIFHOZ	salt	no		
LILWUY	hydrate	no		
LILYAG	anhydrate	no		
LIMBIS	anhydrate	no		
LIMCAL	anhydrate	no		
LIMCEP	anhydrate	no		
LIMNEA	anhydrate	yes	yes	Both listed as poly, one without 3D coord.
LOFDOB	anhydrate	yes	no	
LOFEXD	anhydrate	no		
LOHKUQ	salt	no		
LOKNII	solvate	no		
LONKEE	solvate	no		
LONKII	solvate	no		
LOQSEP	solvate	no		
LOWFUA	anhydrate	no		
LUDYEO	anhydrate	no		
LUFQIO	anhydrate	no		
LUHQIO	cocrystal	no		
LUMNPO	cocrystal	yes	yes	Both listed as poly, one without 3D coord.
LUQKEO	hydrate	no		
LUYJEU	salt	yes	yes, PT	Both listed as poly, one without 3D coord.
MABZOX	anhydrate	yes	no	
MAMMCL	salt	yes	no	
MAPWUV	hydrate	yes	yes	Both listed as poly, one without 3D coord.
MAZXBZ	anhydrate	no		
MBZANQ	anhydrate	yes	yes	Both listed as poly, one without 3D coord.
METMSX	anhydrate	no		
MEWFUR	anhydrate	no		
MHXHQU	anhydrate	no		
MHXHQV	anhydrate	no		

MIBABA	anhydrate	no		
MIHYIL	salt	no		
MNVIMZ	anhydrate	no		
MOCANT	anhydrate	yes	yes	Two listed as poly (diff), two have 3D coord. (same)
MOCYIM	anhydrate	yes	yes	Both listed as poly, one without 3D coord.
MOPHAA	salt	no		
MOVRIY	solvate	no		
MOVROE	solvate	no		
MOXVUR	cocrystal	no		
MOYGUC	anhydrate	yes	yes	Both listed as poly, one without 3D coord.
MPCSZX	anhydrate	no		
MPIMAC	anhydrate	no		
MTHOLI	salt	yes	yes	Both listed as poly, one without 3D coord.
MTHXPH	anhydrate	yes	yes	Both listed as poly, one without 3D coord.
MTKETZ	anhydrate	yes	yes	Two listed as poly (diff), two have 3D coord. (same)
MUCNOO	salt hydrate	yes	no	
MUFNEG	hydrate	no		
MUPQET	solvate	no		
MUTZOQ	anhydrate	no		
MUWTED	anhydrate	yes	yes	Both listed as poly, one without 3D coord.
MXBTCP	anhydrate	no		
MXPBUQ	anhydrate	no		
NAAMPH	salt hydrate	yes	no	
NACVEU	salt	no		
NACVIY	salt	no		
NAJDOS	anhydrate	no		
NAPYDH	hydrate	yes	yes	Two listed as poly (diff), two have 3D coord. (same)
NATLUP	solvate	no		
NAVCIX	anhydrate	yes	yes, PT	Both listed as poly, one without 3D coord.
NAWSUB	anhydrate	yes	yes	Both listed as poly, both have 3D coord., updated in late 2015
NBARBT	hydrate	yes	yes	Two listed as poly (diff), two have 3D coord. (same)
NBUTOL	anhydrate	no		
NBZSES	anhydrate	yes	yes	Both listed as poly, one without 3D coord.
NECWEX	anhydrate	no		
NENGAP	salt	no		

NENGET	salt	no		
NEXPAH	anhydrate	no		
NEXPEL	solvate	no		
NICSAL	salt	yes	yes	Two listed as poly (diff), two have 3D coord. (same)
NIDPIB	solvate	no		
NIQFAV	salt	yes	no	
NIYLUC	solvate	yes	yes	Two listed as poly (diff), two have 3D coord. (same)
NIZVEY	anhydrate	no		
NOHTOT	anhydrate	no		
NOPGUU	anhydrate	yes	yes	Both listed as poly, one without 3D coord.
NOQGIJ	solvate	no		
NOZWII	anhydrate	no		
NOZWOO	salt	no		
NUPBAD	salt	no		
NUPBEH	salt	no		
NUVXAE	anhydrate	no		
OBEHAE	anhydrate	no		
OBEQER	solvate	no		
OBEQIV	cocrystal	no		
OCTATT	anhydrate	no		
OCTCOS	anhydrate	yes	yes	Both listed as poly, one without 3D coord.
OEPYMI	salt	yes	no	
OETPOA	salt solvate	no		
OFOXEN	anhydrate	no		
OGERAU	anhydrate	no		
OGIDEN	anhydrate	no		
OGUROZ	anhydrate	yes	yes	Both listed as poly, one without 3D coord.
OIPFRP	anhydrate	yes	yes	Both listed as poly, one without 3D coord.
OMCSIO	anhydrate	yes	yes, PT	Both listed as poly, one without 3D coord.
OXAZOL	anhydrate	yes	yes, PT	Both listed as poly, one without 3D coord.
OXBICB	anhydrate	yes	yes	Both listed as poly, one without 3D coord.
PABNAJ	anhydrate	no		
PABNIR	anhydrate	no		
PASPLB	anhydrate	yes	yes	All listed as poly, only one with 3D coord.
PASWUD	salt	no		
PASXAK	salt hydrate	yes	yes	Both listed as poly, one without 3D coord.
PBRPOL	anhydrate	no		

PCCOCT	anhydrate	no		
PEFGOX	hydrate	yes	yes	Both listed as poly, both have 3D coord., second added 2016
PEFGUC	cocrystal	yes	yes	Both listed as poly, only one with 3D coord.
PEFZIL	salt	no		
PEJKAS	anhydrate	no		
PEJKIA	anhydrate	no		
PENJAT	anhydrate	no		
PENJAV	anhydrate	no		
PENMPH	anhydrate	yes	yes	Two with 3D coord. (same), three listed as poly
PEPGUM	anhydrate	no		
PETNEI	anhydrate	no		
PETXUH	anhydrate	yes	yes	Both listed as poly, one without 3D coord.
PGLUAC	anhydrate	yes	yes	Both listed as poly, one without 3D coord.
PHARFE	anhydrate	yes	yes	Both listed as poly, one without 3D coord.
PHCBUT	anhydrate	yes	yes	Both listed as poly, one without 3D coord.
PHPYRO	anhydrate	no		
PIBBEH	anhydrate	no		
PIBGOY	doesn’t show up			
PIFHOC	anhydrate	no		
PIFKIA	anhydrate	no		
PIJXEL	anhydrate	yes	yes	Both listed as poly, one without 3D coord.
PILDIX	salt	no		
PIPMDC	hydrate	yes	yes	All listed as poly, only one with 3D coord.
PIVGEH	salt	yes	maybe	Only one listed as poly and having 3D coord., other has neither
PIYSIA	anhydrate	no		
PIYSUM	solvate	no		
PMETRA	salt	yes	yes	Both listed as poly, one without 3D coord.
POBKOG	anhydrate	no		
POPJOV	anhydrate	no		
PROSTA	anhydrate	yes	yes	Both listed as poly, one without 3D coord.
PRSTER	anhydrate	no		
PTBORP	anhydrate	yes	yes	Both listed as poly, one without 3D coord.
PUDWUH	anhydrate	yes	yes	All listed as poly, only one with 3D coord.
PYDPYR	hydrate	yes	yes	Both listed as poly, one without 3D coord.
PYRDCI	salt	yes	yes	Both listed as poly, one without 3D coord.
QABDAZ	anhydrate	no		

QADGEH	cocrystal	yes	yes	Both listed as poly, one without 3D coord.
QAGHAI	anhydrate	no		
QAGHEM	solvate	no		
QAGLOZ	anhydrate	yes	yes	All listed as poly, only one with 3D coord.
QAMQOL	anhydrate	no		
QATNUU	salt	no		
QATPAC	salt	no		
QATPEG	salt	no		
QAYXAP	solvate	no		
QAYXET	solvate hydrate	no		
QEJGOB	anhydrate	yes	yes	Both listed as poly, one without 3D coord.
QESHUS	anhydrate	no		
QETZUL	anhydrate	no		
QIBQIB	anhydrate	yes	yes	Both listed as poly, one without 3D coord.
QIFNIE	anhydrate	no		
QIKZUH	anhydrate	yes	no	
QIMXUH	salt hydrate	no		
QIYKEP	salt	no		
QIYKIT	salt	no		
QIYKOZ	salt	no		
QOCDOB	anhydrate	yes	yes	Both listed as poly, one without 3D coord.
QOJNOU	doesn't show up			
QOLVOD	anhydrate	no		
QOPBED	anhydrate	yes	no	
QOPZUQ	salt	no		
QOQBAZ	salt	no		
QOTBIM	solvate solvate (2 separate)	no		
QOYKIY	anhydrate	no		
QQQAKS	anhydrate	yes	yes	All listed as poly, only one with 3D coord.
QQQAPG	anhydrate	yes	yes	All listed as poly, only one with 3D coord.
QQQBDJ	salt hydrate	yes	yes	All listed as poly, only one with 3D coord.
QQQBLM	anhydrate	yes	yes	Both listed as poly, one without 3D coord.
QQQBNV	salt solvate	yes	yes	Both listed as poly, one without 3D coord.
QQQBRD	anhydrate	yes	yes	All listed as poly, only one with 3D coord.
QQQBVP	anhydrate	yes	yes	All listed as poly, only one with 3D coord.
QQQCZS	anhydrate	yes	yes	Both listed as poly, one without 3D coord.
QQQDHD	salt	yes	yes	Both listed as poly, one without 3D coord.

QQQDZG	salt	yes	yes	Both listed as poly, one without 3D coord.
QQQFAA	anhydrate	yes	yes, PT	Both listed as poly, one without 3D coord.
QQQFAD	anhydrate	yes	yes, PT	All listed as poly, only one with 3D coord.
QQQFBP	anhydrate	yes	no	
QQQFDS	anhydrate	yes	yes	All listed as poly, only one with 3D coord.
QQQFDY	anhydrate	yes	yes	All listed as poly, only one with 3D coord.
QQQFED	anhydrate	yes	yes	All listed as poly, only one with 3D coord.
QQQFGD	salt	yes	yes	All listed as poly, only one with 3D coord.
QQQFHJ	anhydrate	yes	yes	Both listed as poly, one without 3D coord.
QQQFVD	anhydrate	yes	yes	All listed as poly, only one with 3D coord.
QQQGTS	solvate	yes	yes	Both listed as poly, one without 3D coord.
QQQHDS	anhydrate	yes	yes	Both listed as poly, one without 3D coord.
QUFHAA	solvate	no		
RALQIE	anhydrate	no		
RAMGOB	anhydrate	yes	yes	All listed as poly, all have 3D coord., all added late 2015 and 2016
RASTIO	anhydrate	no		
REHTII	salt	yes	no	
RIZXON	anhydrate	yes	yes	Both listed as poly, one without 3D coord.
RUGDUS	hydrate	no		
RUGFII	anhydrate	no		
RUKGUZ	hydrate	no		
RUKHAG	anhydrate	yes	yes	Both listed as poly, both have 3D coord., one added 2016
RUPFIS	anhydrate	yes	yes	Both listed as poly, one without 3D coord.
RUZWUE	anhydrate	no		
RVOXMI	salt	yes	yes	Both listed as poly, one without 3D coord.
SACXID	anhydrate	no		
SACXOJ	anhydrate	no		
SADJAI	anhydrate	yes	no	
SANCEP	anhydrate	yes	yes, PT	Both listed as poly, one without 3D coord.
SAWKUX	solvate	no		
SBBCHD	anhydrate	no		
SEBFAG	anhydrate	no		
SEBFAH	solvate	no		
SEGXAE	anhydrate	no		
SEKXUC	anhydrate	yes	yes	Both listed as poly, one without 3D coord.
SEQBAR	salt	no		
SETLOU	anhydrate	no		

SEYHOT	salt	no		
SEZXIE	salt	yes	no	
SINKEF	anhydrate	yes	yes	Both listed as poly, one without 3D coord.
SIQGOO	anhydrate	no		
SITKEL	solvate	no		
SIXCIM	salt hydrate	no		
SIXFIP	anhydrate	no		
SOGNUX	solvate	no		
SOHXUI	anhydrate	yes	yes	Both listed as poly, one without 3D coord.
SOXHUI	anhydrate	no		
SOXLAS	salt hydrate	no		
SOXVEG	anhydrate	no		
SOXVIK	anhydrate	no		
STENON	salt hydrate	no		
SUHVEW	anhydrate	no		
SUSJEV	anhydrate	no		
SUSKAS	anhydrate	no		
SUTWIO	anhydrate	no		
SUWMAY	anhydrate	no		
TAJQUR	salt	no		
TAPMEC	salt	no		
TARTAM	anhydrate	yes	yes	All listed as poly, only one with 3D coord.
TATNBZ	anhydrate	yes	yes	All listed as poly, only one with 3D coord.
TBUBEN	anhydrate	no		
TBUHLB	anhydrate	yes	yes, PT	All listed as poly, only one with 3D coord.
TBZMBA	anhydrate	yes	yes	Both listed as poly, one without 3D coord.
TCAPIN	anhydrate	no		
TCBCAO	anhydrate	no		
TCLCYH	anhydrate	yes	yes	Both listed as poly, one without 3D coord.
TCMPCQ	anhydrate	yes	no	
TCTCPH	anhydrate	no		
TDCHCH	anhydrate	yes	yes	All listed as poly, only one with 3D coord.
TEBHEN	salt	no		
TEBSSU	anhydrate	yes	yes	Both listed as poly, one without 3D coord.
TEMCIX	cocrystal hydrate	no		
TEPSEM	anhydrate	no		
TETGUU	anhydrate	no		
TEYJEM	anhydrate	yes	yes	Both listed as poly, one without 3D coord.

TEZNUJ	anhydrate	no		
TFMCYP	anhydrate	no		
TGLYCY	anhydrate	yes	yes	All listed as poly but one, only two have 3D coord. (one of which wasn't poly, but those two are the same form)
THFUCA	salt	yes	yes	Both listed as poly, one without 3D coord.
THIMHC	salt hydrate	no		
TIHVUC	anhydrate	yes	yes	Both listed as poly, both have 3D coord, second added in 2016
TIHWAJ	anhydrate	no		
TIHWEN	anhydrate	no		
TIHYAL	anhydrate	no		
TIHYUF	anhydrate	no		
TIJBAQ	anhydrate	no		
TIJSIP	solvate	no		
TIJSOV	solvate	no		
TIJSUB	solvate	no		
TIJTAI	solvate	no		
TIJTEM	solvate	no		
TIJTIQ	solvate	no		
TIKBOE	salt	yes	yes	All listed as poly, only one with 3D coord.
TIKIND	anhydrate	yes	yes	Both listed as poly, one without 3D coord.
TIPHAM	anhydrate	yes	yes	Both listed as poly, one without 3D coord.
TIPHUW	solvate	no		
TIPJAE	solvate	no		
TIPMEP	salt	no		
TITSOG	salt	no		
TIZVUT	hydrate	no		
TMBRBZ	anhydrate	yes	yes	Two have 3D coord. (same), one of those added 2016, other listed as poly with no 3D coord.
TMDHCB	anhydrate	yes	yes	Both listed as poly, one without 3D coord.
TMOSCA	anhydrate	yes	yes	All listed as poly, only one with 3D coord.
TMURIC	anhydrate	yes	yes	Both listed as poly, one without 3D coord.
TOSDAZ	anhydrate	yes	yes	Both listed as poly, one without 3D coord.
TPABAN	anhydrate	yes	yes, PT	All listed as poly, only one with 3D coord.
TPCHDA	cocrystal	no		
TPCHDB	cocrystal	no		
TPHNAP	anhydrate	yes	yes	Both listed as poly, one without 3D coord.
TUBRAJ	anhydrate	yes	yes	All listed as poly, only one with 3D coord.
TUSSIJ	salt	yes	yes, PT	Both listed as poly, one without 3D coord.

TZAZPC	anhydrate	yes	yes	Both listed as poly, one without 3D coord.
UBEJIW	salt	no		
UBUREO	anhydrate	yes	continual PT	Only one listed as poly, no distinct difference
UDAZII	solvate hydrate	yes	yes	Both listed as poly, one without 3D coord.
UDELEV	hydrate	no		
UHAWEF	salt	no		
UHUHOU	solvate	no		
UKIFIE	salt	no		
UNDEAC	anhydrate	no		
UXIYIK	anhydrate	no		
UYAZEA	cocrystal	no		
VAFCEK	anhydrate	yes	yes	Both listed as poly, one without 3D coord.
VALINM	solvate	no		
VAMZIT	anhydrate	yes	yes, PT	Both listed as poly, one without 3D coord.
VAMZOZ	anhydrate	yes	yes, PT	Both listed as poly, one without 3D coord.
VAMZUF	anhydrate	yes	yes, PT	Both listed as poly, one without 3D coord.
VANXAK	solvate	yes	no	
VARKII	anhydrate	yes	yes	Both listed as poly, one without 3D coord.
VARNEH	anhydrate	no		
VEBCAH	anhydrate	no		
VECXIK	anhydrate	yes	no	
VECXUW	anhydrate	yes	no	
VECYAD	anhydrate	yes	no	
VEFLIB	anhydrate	yes	yes	All listed as poly, only one with 3D coord.
VEFYAG	solvate	no		
VENVIT	anhydrate	yes	yes	All listed as poly, only one with 3D coord.
VESTIX	salt	no		
VEXKEO	anhydrate	no		
VEYVEA	hydrate	no		
VHVIMH	anhydrate	no		
VIBKIA	anhydrate	no		
VIJWEQ	anhydrate	no		
VIVRIB	salt	yes	yes	All listed as poly, only one with 3D coord.
VIXPOJ	anhydrate	yes	yes	Both listed as poly, both have 3D coord., second added 2016
VODGUR	anhydrate	no		
VOTTON	anhydrate	yes	yes	Both listed as poly, one without 3D coord.
VUJTOJ	anhydrate	no		
WAHSAZ	anhydrate	no		

WAHSEC	anhydrate	no		
WAMMAY	anhydrate	no		
WANLAY	anhydrate	yes	no	
WANMON	anhydrate	yes	yes	All listed as poly, only one with 3D coord.
WAPCAR	solvate	no		
WAVYAT	anhydrate	no		
WAYCUW	anhydrate	no		
WEJNEE	anhydrate	no		
WERZOI	anhydrate	no		
WESBAX	anhydrate	no		
WIFCAQ	anhydrate	no		
WIHBAS	anhydrate	no		
WIMTER	anhydrate	no		
WIMWOE	anhydrate	no		
WIMZUN	anhydrate	no		
WINBEA	anhydrate	no		
WINBIE	hydrate	no		
WINBIG	hydrate	no		
WINDEC	anhydrate	no		
WIQLEN	anhydrate	no		
WISBEF	salt solvate	no		
WISGEK	anhydrate	no		
WISRAR	anhydrate	no		
WIYVEF	salt	yes	yes, PT	Both listed as poly, one without 3D coord.
WOBQIO	cocrystal	no		
WOJXUO	anhydrate	yes	yes	Both listed as poly, one without 3D coord.
WOVMID	hydrate	no		
XAFMIC	anhydrate	no		
XEBKEV	anhydrate	no		
XEDVIM	anhydrate	no		
XEDVOS	anhydrate	no		
XEGMAX	anhydrate	yes	yes	Both listed as poly, one without 3D coord.
XEHQAC	anhydrate	no		
XEHQEG	anhydrate	no		
XENDUP	solvate	no		
XEZFIT	anhydrate	no		
XIHQEK	anhydrate	no		
XILREP	anhydrate	no		

XISJUE	anhydrate	no		
XISLUG	salt	yes	no	
XOKMOZ	anhydrate	yes	yes	Both listed as poly, one without 3D coord.
XOZJOM	salt hydrate	yes	no	
XUVNIM	anhydrate	no		
XUVZET	anhydrate	no		
YAJFOG	anhydrate	no		
YALYUH	anhydrate	yes	yes	Both listed as poly, one without 3D coord.
YANDAT	anhydrate	no		
YAQLEI	anhydrate	yes	yes, PT	Both listed as poly, one without 3D coord.
YAQLIM	anhydrate	no		
YAWMOY	anhydrate	yes	maybe	All listed as poly, only one with 3D coord.
YAWPIV	solvate	no		
YEFWEM	anhydrate	yes	no	
YENGAZ	anhydrate	yes	yes	Both listed as poly, one without 3D coord.
YETPES	anhydrate	no		
YETPIW	hydrate	no		
YIBVEL	anhydrate	no		
YIBVIP	anhydrate	no		
YICGEW	anhydrate	yes	yes	All listed poly, all have 3D coord., last two added 2017
YIFYAP	anhydrate	no		
YIPFUY	anhydrate	yes	yes, PT	Both listed as poly, one without 3D coord.
YIXTIJ	anhydrate	no		
YIXTOP	anhydrate	no		
YIXTUV	anhydrate	no		
YIZXIO	solvate	no		
YOKSOG	salt	yes	yes, PT	All listed as poly, only one with 3D coord.
YOLYUU	anhydrate	yes	yes, PT	All listed as poly, only one with 3D coord.
YUFCUZ	cocrystal solvate	no		
YUFDAG	cocrystal solvate	no		
YUFDEK	cocrystal	no		
YUFDIO	cocrystal	no		
YUFDOU	cocrystal solvate	no		
YUFDUA	cocrystal	no		
YUFFAI	cocrystal	no		

YUFFEM	cocrystal solvate	no		
YUFFIQ	cocrystal	no		
YUFFOW	cocrystal solvate	no		
YUFGEN	cocrystal solvate	no		
YUFGIR	cocrystal hydrate	no		
YUYDOL	anhydrate	no		
YUYMIO	anhydrate	yes	yes	All listed as poly, only one with 3D coord.
ZADREB	solvate hydrate	no		
ZADRIF	anhydrate	no		
ZADWOQ	solvate	no		
ZASQOZ	anhydrate	no		
ZEDGAQ	salt	yes	yes	Both listed as poly, one without 3D coord.
ZEGLUS	anhydrate	no		
ZEGMAZ	anhydrate	no		
ZEKPEM	hydrate	no		
ZEKPIQ	hydrate	no		
ZEMXOE	salt	no		
ZEXZIN	salt	yes	yes	Both listed as poly, one without 3D coord.
ZIWHOC	hydrate	no		
ZIWHUI	hydrate	no		
ZIWJAQ	hydrate	no		
ZIWYUZ	solvate	no		
ZOMDEM	solvate	yes	no	
ZUDTAT	anhydrate	yes	no	
ZUSLIK	anhydrate	no		
ZUZYAU	hydrate	no		
ZZZAKJ	anhydrate	yes	no	
ZZZAKP	anhydrate	yes	no	
ZZZAKV	anhydrate	yes	no	
ZZZAOV	anhydrate	yes	yes	Both listed as poly, one without 3D coord.
ZZZAWV	anhydrate	yes	no	
ZZZAYP	anhydrate	yes	yes	All listed as poly, only one with 3D coord.
ZZZBPD	anhydrate	yes	no	
ZZZFLM	anhydrate	yes	no	
ZZZHUW	anhydrate	yes	yes	All listed as poly, only one with 3D coord.
ZZZKAO	anhydrate	yes	yes	Both listed as poly, one without 3D coord.

ZZZKDW	anhydrate	yes	yes	Both listed as poly, one without 3D coord.
ZZZKGE	anhydrate	yes	yes	All listed as poly, only one with 3D coord.
ZZZMAI	salt hydrate	yes	yes	Both listed as poly, one without 3D coord.
ZZZMBE	anhydrate	yes	yes	All listed as poly, only one with 3D coord.
ZZZMJK	solvate	yes	yes	All listed as poly, only one with 3D coord.
ZZZNQQ	anhydrate	yes	yes	Both listed as poly, one without 3D coord.
ZZZNRU	anhydrate	yes	no	
ZZZNYY	anhydrate	yes	no	
ZZZODM	salt	yes	no	
ZZZOEG	anhydrate	yes	yes	All listed as poly, only two have 3D coord. (same)
ZZZOFC	anhydrate	yes	yes	All listed as poly, only one with 3D coord.
ZZZOPV	anhydrate	yes	yes	All listed as poly, only one with 3D coord.
ZZZOQK	anhydrate	yes	yes	Both listed as poly, one without 3D coord.
ZZZPLY	anhydrate	yes	yes	All listed as poly, only one with 3D coord.
ZZZPMI	anhydrate	yes	yes	Both listed as poly, one without 3D coord.
ZZZQJS	anhydrate	yes	yes	All listed as poly, only one with 3D coord.
ZZZSHS	salt	yes	yes	Both listed as poly, one without 3D coord.
ZZZVYE	anhydrate	yes	yes	Both listed as poly, one without 3D coord.
ZZZWEQ	anhydrate	yes	yes, PT	All listed as poly, only one with 3D coord.

Table S6 Description of compounds with multiple entries in the polymorph list (shown as yellow in Table S3) that are not included in Table S4 (180 refcodes).

Refcode	More in CSD than already listed?	Should it be included?	Details
ADAMAN	yes	no	No 3D coord.
ANTCEN	yes	no	Some don't have 3D coord., last three added 2015 and not flagged as poly but also not new forms
ANTMEU	yes	no	No 3D coord.
AWAJOX	yes	no	Some not flagged as poly but all same anyways
BADPEB	yes	no	No 3D coord.
BIZWAI	yes	no	No 3D coord.
BNPYRE	yes	no	No 3D coord.
BOMKUJ	no		
BOMWIJ	yes	no	No 3D coord.
BOWMIK	no		
BOWROU	yes	no	No 3D coord.
BOYFOK	yes	no	No 3D coord.
BPYZDO	no		
BRFUSO	yes	no	No 3D coord.
BTRILA	yes	no	No 3D coord.
BUFNEV	no		
CAXWUT	yes	no	No 3D coord.
CECPAB	yes	no	No 3D coord.
CHOCHL	yes	no	No 3D coord.
CINNDS	no		
CLAMPL	yes	no	No 3D coord.
COQNUR	no		
CORYIR	no		
CTBROM	yes	no	No 3D coord.
CUHDAK	no		
DALJAB	yes	no	No 3D coord.
DANTEN	yes	no	Not flagged as poly but same
DARGOT	yes	no	No 3D coord.
DAXLIZ	no		
DCHXYK	no		
DGLYAC	yes	no	No 3D coord.
DIXNIH	yes	no	No 3D coord.

DLASPA	yes	no	Some have no 3D coord., one added 2016, one not flagged as poly but same
DLSERN	yes	no	Three new: one no 3D coord., one not flagged as poly but 2016, one not flagged as poly but same
DMEACL	yes	no	No 3D coord.
DNTNAP	yes	no	No 3D coord.
DOMBOW	no		
DOSQAD	no		
DOWVOC	yes	no	All added early enough, all modified in 2016, listed as PT but all same regardless of temp
DPHOCE	yes	no	No 3D coord.
DQUNDS	yes	no	No 3D coord.
DUHSEE	yes	no	No 3D coord.
EDTAXX	yes	no	No 3D coord.
EJEROA	yes	no	No 3D coord.
EJIQEU	no		
FAWVOO	no		
FEYLUQ	no		
FIHYEA	yes	no	No 3D coord.
FIVSIM	no		
FIYVAK	yes	no	No 3D coord.
FOMGIY	no		
FOTYUI	no		
FUFNOJ	yes	no	No 3D coord.
FUPDOJ	no		
FUYYON	yes	no	No 3D coord.
FUYZEE	yes	no	No 3D coord.
GICYUN	no		
HECXAO	yes	no	No 3D coord.
HERMOH	no		
HEXBME	yes	no	Some no 3D coord., last one 2016
HEXDEC	yes	no	No 3D coord.
HEYJOK	no		
HOBBOP	yes	no	No 3D coord.
HOCYSL	no		
HOLVAG	no		
HXCFUL	yes	no	No 3D coord.
IQACIM	yes	no	No 3D coord.

IYUXUW	no		
JIPJAT	no		
JIPJEX	no		
JOJYOY	yes	no	Not flagged as poly but same
JORMAE	no		
KANQAR	no		
KGLUCP	yes	no	No 3D coord.
KIBBUS	no		
KIBQOC	no		
KIXLOS	no		
KTMSIO	no		
LDOPAS	yes	no	No 3D coord.
LIFORM	yes	no	No 3D coord.
LILXIN	yes	no	No 3D coord.
LULKIN	no		
MECHLF	yes	no	No 3D coord.
METACM	yes	no	One no 3D coord., one not flagged as poly but same
MPPTCQ	yes	no	No 3D coord.
MPYRAZ	yes	no	No 3D coord.
MPYZBD	yes	no	No 3D coord.
NAPHAC	yes	no	No 3D coord.
NAPHOB	yes	no	No 3D coord.
NAPPYR	yes	no	No 3D coord.
NECNEO	no		
NEPGCL	yes	no	One no 3D coord., one not flagged as poly but same
NIBZAL	no		
NIPHSS	no		
OSAGEV	yes	no	Not flagged as poly but same
OXIBZN	yes	no	No 3D coord.
OXTCNT	yes	no	One no 3D coord., one added 2016
OXTPTZ	no		
PAJZAC	no		
PARTEN	yes	no	No 3D coord.
PAZOXN	yes	no	One no 3D coord., one not flagged as poly but same
PERYTO	yes	no	Some no 3D coord., one added 2016, one not flagged as poly but same
PIDGOZ	yes	no	Not flagged as poly but same

PIVIND	no		Original space group was refined by another author. Same structure
POKSAJ	yes	no	No 3D coord.
PRMDIN	yes	no	Several not flagged as poly, one listed as form II but same for all
PTRPHT	yes	no	No 3D coord.
PUBMII	yes	no	Last one added 2016
PULHUZ	no		Not different form
PYRAZI	yes	no	One no 3D coord., rest Dec. 2015
QQQACY	yes	no	No 3D coord.
QQQBTP	yes	no	No 3D coord.
QQQCVG	yes	no	No 3D coord.
QQQFES	yes	no	No 3D coord.
RAKWIJ	no		
RBHACD	yes	no	No 3D coord.
RBHTCA	no		
REJNUP	no		
RORNIV	no		
RUKDAD	yes	no	One no 3D coord., one not flagged as poly but same
SACBAA	yes	no	One no 3D coord., one added Nov 2016
SEUREA	yes	no	One no 3D coord., others added 2017
SLFNMD	yes	no	No 3D coord.
SLFNMF	yes	no	No 3D coord.
SOLSIW	no		Same
SOMTUJ	no		
STILTQ	yes	no	One no 3D coord., other same, better assignment of space group
SUSKUM	no		
TANTOX	yes	no	No 3D coord.
TCHXYP	yes	no	No 3D coord.
TEBETU	yes	no	No 3D coord.
TEBGUC	no		
TETDAM	yes	no	No 3D coord.
TETTRI	yes	no	No 3D coord.
TFMETH	yes	no	No 3D coord.
THXNOO	no		
TIPSAM	no		
TIPWIY	no		

TIWTAW	no		
TOHBPA	yes	no	No 3D coord.
TOLUEN	yes	no	One no 3D coord., rest added 2016
TRITAN	no		Redetermination of a superlattice
TRIZIN	yes	no	No 3D coord.
TUPRBN	no		Redetermination
UREAXX	yes	no	Some no 3D coord., several added 2016, one not flagged as poly but same
VALINO	no		
VARYES	no		
VEJXAJ	no		
VOBHAX	no		Different temps, same structure
VUCFUU	no		
WEMGAW	yes	no	No 3D coord.
WEMGIE	yes	no	No 3D coord.
WERZUO	no		
WIRYEB	yes	no	No 3D coord.
WOKMUG	no		
WORMAN	yes	no	No 3D coord.
XAZHOW	no		
XEXKEQ	no		
XYLTOL	yes	no	Added Nov. 2015 and 2016
YESXUP	yes	no	No 3D coord.
YIQGEL	no		
YISJUF	yes	no	One with no 3D coord., of other two, one is superlattice
YUXCEZ	no		
ZAYKAL	yes	no	Added 2016
ZBCNON	no		
ZEFXIR	yes	no	One with no 3D coord., one added 2016. Of first two, second is redetermination of space group
ZEXPEX	no		
ZIRGUC	no		Second is improved structure from same people
ZZZBFM	yes	no	No 3D coord.
ZZZDDP	yes	no	No 3D coord.
ZZZHKQ	yes	no	No 3D coord.
ZZZNNM	yes	no	No 3D coord.
ZZZOYC	yes	no	No 3D coord.

ZZZPPI	yes	no	One with no 3D coord., of other two, second one is a better solving
ZZZPZE	yes	no	No 3D coord.
ZZZQSW	yes	no	No 3D coord.
ZZZUCY	yes	no	Two without 3D coord., one private communication but same as other
ZZZUQO	yes	no	Others no 3D coord., of two, one is reinterpretation
ZZZUWK	yes	no	No 3D coord.
ZZZWEO	yes	no	No 3D coord.

S6. Details of Polymorphism Tree Searching (Figure 2 in main text)

Searches of the CSD (Groom, 2016) detailed below were conducted with ConQuest version 1.18 with the restrictions of 3D coordinates known and organics only.

Single components:

A search was conducted restricting to one chemical unit under Z/Density, and restricting entries to those not containing the name ‘hydrate’ or the name ‘solvate’. This gives entries that should contain one neutral molecular unit (223,483 hits).

Multicomponent systems:

The search for salts involved analysis of any entry that contained two or more chemical units under Z/Density, giving 93,927 hits. Adding ‘no ions’ to this search resulted in 47,754, indicating that 46,173 hits contained charged species and were considered salts for this case.

The search for hydrates involved entries containing ‘hydrate’ in the name or a drawing of H-O-H to account for cases when water was not explicitly named as a hydrate (26,949 hits).

A simple search for solvates involved a text search for the word ‘solvate’ which gave 31,948 hits. However, solvates can also be listed under the term clathrate, a term used to designate host guest compounds, but the guest can be a solid or liquid. For this purpose, only clathrates that contain liquids are included. A listing of entries with the term clathrate that are not already in the solvate list produces 5,444 hits. These are analyzed to remove solid guests and 3,117 solvates were determined. Added with those in the search for just solvate, the total is 35,065.

To determine cocrystals, several searches were conducted:

A search for 2 chemical units with no ions, no hydrates, and no solvates would be two neutral components (11,314 hits). Not every one of these entries shows cocrystals; however, as some were clathrates or unlisted solvates, this list needed to be individually sorted through to find the number of cocrystal entries in this group (7,080 hits). A search for 3 or more chemical units could contain cocrystals plus a solvent, or two solvents and one neutral molecule, as well as salts and/or ionic cocrystals. This search gave 25,667 hits and these were individually analyzed to find entries containing at least two neutral components that are solids at room temperature (5,712 hits). Added together, this results in 12,792 cocrystal entries.

Families in each category were determined by finding the number of unique refcodes in each list.

The number of polymorph entries were determined by adding a text search for “polymorph” to any of the crystal type searches outlined above.

The number of polymorph families were determined by finding the number of unique refcodes in each polymorph entries list.

The number of polymorphic compounds are the numbers determined from the previously outlined search in section SI 2, with the data presented in Table S4. Breakdowns for each multicomponent crystal type also come from the data in Table S4, and show the combination multicomponent systems (such as cocrystal salts or hydrate solvates, for example) in each crystal type.

S7. Raw Data from Percentage of Polymorphism Searches

Table S7 Data for calculating percentage of polymorphs in all organics from 1991-2015

Year	Organic Entries	Polymorph Entries	Poly/Org %	New Org	New Poly	New poly/ new org %
1991	41212	2387	5.79			
1992	46632	2547	5.46	5420	160	2.95
1993	50728	2682	5.29	4096	135	3.30
1994	54909	2836	5.16	4181	154	3.68
1995	59926	3002	5.01	5017	166	3.31
1996	65772	3181	4.84	5846	179	3.06
1997	71079	3375	4.75	5307	194	3.66
1998	76883	3565	4.64	5804	190	3.27
1999	83529	3773	4.52	6646	208	3.13
2000	89901	3962	4.41	6372	189	2.97
2001	94818	4138	4.36	4917	176	3.58
2002	104756	4462	4.26	9938	324	3.26
2003	114181	4817	4.22	9425	355	3.77
2004	124586	5221	4.19	10405	404	3.88
2005	135626	5638	4.16	11040	417	3.78
2006	149477	6167	4.13	13851	529	3.82
2007	163143	6633	4.07	13666	466	3.41
2008	177135	7146	4.03	13992	513	3.67
2009	189967	7610	4.01	12832	464	3.62
2010	204177	8075	3.95	14210	465	3.27
2011	224995	8713	3.87	20818	638	3.06
2012	245169	9350	3.81	20174	637	3.16
2013	258837	9818	3.79	13668	468	3.42
2014	286680	10803	3.77	27843	985	3.54
2015	318524	11909	3.74	31844	1106	3.47

Table S8 Data for calculating percentage of polymorphs in organics with 1 molecular unit from 1991-2015

Year	Organic Entries 1 Mol. Unit	Poly Entries 1 Mol. Unit	Poly/Org %	New Org 1 Mol. Unit	New Poly 1 Mol. Unit	New Poly/ New Org %
1991	30257	1809	5.98			
1992	34277	1934	5.64	4020	125	3.11
1993	37387	2043	5.46	3110	109	3.50
1994	40428	2142	5.30	3041	99	3.26
1995	44095	2266	5.14	3667	124	3.38
1996	48254	2403	4.98	4159	137	3.29
1997	52076	2555	4.91	3822	152	3.98
1998	56272	2676	4.76	4196	121	2.88
1999	60901	2828	4.64	4629	152	3.28
2000	65339	2967	4.54	4438	139	3.13
2001	68803	3114	4.53	3464	147	4.24
2002	75680	3351	4.43	6877	237	3.45
2003	82280	3599	4.37	6600	248	3.76
2004	89537	3894	4.35	7257	295	4.07
2005	97407	4242	4.35	7870	348	4.42
2006	107051	4672	4.36	9644	430	4.46
2007	116627	5023	4.31	9576	351	3.67
2008	126607	5410	4.27	9980	387	3.88
2009	135645	5771	4.25	9038	361	3.99
2010	145484	6110	4.20	9839	339	3.45
2011	159936	6580	4.11	14452	470	3.25
2012	173940	7076	4.07	14004	496	3.54
2013	183170	7406	4.04	9230	330	3.58
2014	202364	8078	3.99	19194	672	3.50
2015	224617	8871	3.95	22253	793	3.56

Table S9 Data for calculating percentage of polymorphs in organics with 2+ molecular units from 1991-2015

Year	Org Entries 2+ Mol. Unit	Poly Entries 2+ Mol. Unit	Poly/Org %	New Org 2+ Mol. Unit	New Poly 2+ Mol. Unit	New Poly/ New Org %
1991	10955	578	5.28			
1992	12355	613	4.96	1400	35	2.50
1993	13341	639	4.79	986	26	2.64
1994	14481	694	4.79	1140	55	4.82
1995	15831	736	4.65	1350	42	3.11
1996	17518	778	4.44	1687	42	2.49
1997	19003	820	4.32	1485	42	2.83
1998	20611	889	4.31	1608	69	4.29
1999	22625	945	4.18	2014	56	2.78
2000	24562	995	4.05	1937	50	2.58
2001	26015	1024	3.94	1453	29	2.00
2002	29076	1111	3.82	3061	87	2.84
2003	31901	1218	3.82	2825	107	3.79
2004	35049	1327	3.79	3148	109	3.46
2005	38219	1396	3.65	3170	69	2.18
2006	42426	1495	3.52	4207	99	2.35
2007	46516	1610	3.46	4090	115	2.81
2008	50528	1736	3.44	4012	126	3.14
2009	54322	1839	3.39	3794	103	2.72
2010	58693	1965	3.35	4371	126	2.88
2011	65059	2133	3.28	6366	168	2.64
2012	71229	2274	3.19	6170	141	2.29
2013	75667	2412	3.19	4438	138	3.11
2014	84316	2725	3.23	8649	313	3.62
2015	93907	3038	3.24	9591	313	3.26

Table S10 Data for calculating percentage of polymorphs in organics with 2 molecular units from 1991-2015

Year	Org Entries CC 2 Mol. Units	Poly Entries CC 2 Mol. Units	Poly/Org %
1991	728	44	6.04
1992	832	45	5.41
1993	892	46	5.16
1994	973	51	5.24
1995	1057	52	4.92
1996	1163	58	4.99
1997	1283	68	5.30
1998	1426	80	5.61
1999	1567	89	5.68
2000	1703	96	5.64
2001	1851	100	5.40
2002	2103	116	5.52
2003	2379	142	5.97
2004	2648	164	6.19
2005	2837	168	5.92
2006	3205	182	5.68
2007	3470	193	5.56
2008	3722	213	5.72
2009	3958	232	5.86
2010	4293	261	6.08
2011	4761	289	6.07
2012	5244	330	6.29
2013	5651	366	6.48
2014	6386	436	6.83
2015	7121	515	7.23

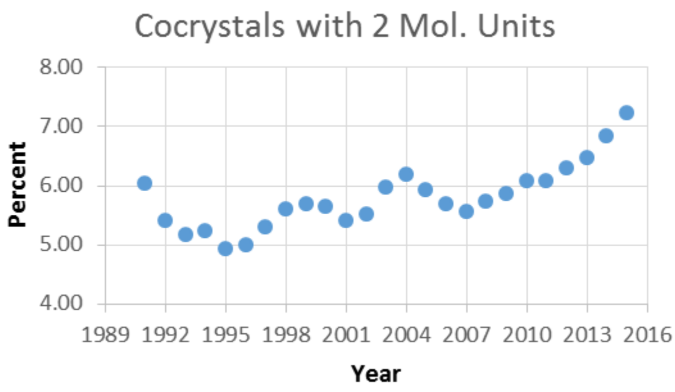

Figure S1 Percentage of polymorphs versus organics in the CSD for cocrystals with only 2 molecular units.

Table S11 Data for calculating percentage of polymorphs in organics with 3+ molecular units from 1991-2015

Year	Org Entries CC 3+ Mol. Units	Poly Entries CC 3+ Mol. Units	Poly/Org %
1991	574	11	1.92
1992	623	11	1.77
1993	656	11	1.68
1994	681	13	1.91
1995	721	13	1.80
1996	799	15	1.88
1997	877	15	1.71
1998	977	16	1.64
1999	1129	17	1.51
2000	1255	17	1.35
2001	1345	17	1.26
2002	1529	23	1.50
2003	1664	24	1.44
2004	1882	29	1.54
2005	2056	29	1.41
2006	2311	34	1.47
2007	2538	40	1.58
2008	2755	42	1.52
2009	3009	48	1.60
2010	3264	48	1.47
2011	3779	55	1.46
2012	4208	55	1.31
2013	4538	61	1.34
2014	5099	71	1.39
2015	5740	82	1.43

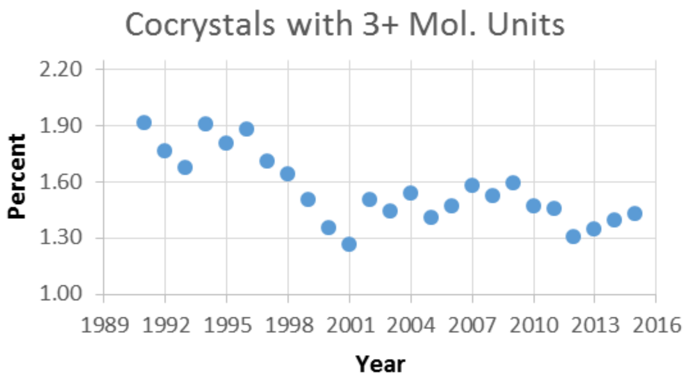

Figure S2 Percentage of polymorphs versus organics in the CSD for cocrystals with 3+ molecular units.

Table S12 Data for calculating percentage of polymorphs in organic hydrates from 1991-2015

Year	Org Hydrate Entries	Poly Hydrate Entries	Poly Hydrate/ Org Hydrate %	New Org Hydrate	New Poly Hydrate	New Poly/ New Org %
1991	3711	130	3.50			
1992	4120	137	3.33	409	7	1.71
1993	4425	142	3.21	305	5	1.64
1994	4719	154	3.26	294	12	4.08
1995	5104	163	3.19	385	9	2.34
1996	5584	179	3.21	480	16	3.33
1997	5980	192	3.21	396	13	3.28
1998	6380	211	3.31	400	19	4.75
1999	6890	225	3.27	510	14	2.75
2000	7466	237	3.17	576	12	2.08
2001	7824	243	3.11	358	6	1.68
2002	8678	259	2.98	854	16	1.87
2003	9453	274	2.90	775	15	1.94
2004	10295	294	2.86	842	20	2.38
2005	11211	297	2.65	916	3	0.33
2006	12442	328	2.64	1231	31	2.52
2007	13616	341	2.50	1174	13	1.11
2008	14823	367	2.48	1207	26	2.15
2009	15959	388	2.43	1136	21	1.85
2010	17276	418	2.42	1317	30	2.28
2011	19186	447	2.33	1910	29	1.52
2012	20994	475	2.26	1808	28	1.55
2013	22208	500	2.25	1214	25	2.06
2014	24518	533	2.17	2310	33	1.43
2015	26684	570	2.14	2166	37	1.71

Table S13 Data for calculating percentage of polymorphs in organic salts from 1991-2015

Year	Org Salt Entries	Poly Salt Entries	Poly Salt / Org Salt %	New Org Salt	New Poly Salt	New Poly/ New Org %
1991	6646	395	5.94			
1992	7403	422	5.70	757	27	3.57
1993	7870	439	5.58	467	17	3.64
1994	8467	471	5.56	597	32	5.36
1995	9238	496	5.37	771	25	3.24
1996	10121	517	5.11	883	21	2.38
1997	10875	542	4.98	754	25	3.32
1998	11662	575	4.93	787	33	4.19
1999	12711	599	4.71	1049	24	2.29
2000	13665	625	4.57	954	26	2.73
2001	14347	640	4.46	682	15	2.20
2002	15774	687	4.36	1427	47	3.29
2003	17063	750	4.40	1289	63	4.89
2004	18440	801	4.34	1377	51	3.70
2005	19991	845	4.23	1551	44	2.84
2006	21947	883	4.02	1956	38	1.94
2007	23916	966	4.04	1969	83	4.22
2008	25834	1041	4.03	1918	75	3.91
2009	27663	1104	3.99	1829	63	3.44
2010	29686	1168	3.93	2023	64	3.16
2011	32681	1269	3.88	2995	101	3.37
2012	35599	1321	3.71	2918	52	1.78
2013	37735	1386	3.67	2136	65	3.04
2014	41855	1586	3.79	4120	200	4.85
2015	50249	1875	3.73	8394	289	3.44

S8. References

Crystal Growth & Design. http://pubs.acs.org/journal/cgdefu. 2017, American Chemical Society.

Groom, C. R., Bruno, I. J., Lightfoot, M. P., and Ward, S. C. The Cambridge Structural Database. (2016). *Acta Crystallogr., Sect. B: Struct. Sci.* **72**, 171-179.

Merz, K. and Kupka, A. Deuterium Perturbs the Molecular Arrangement in the Solid State. (2015). *Cryst. Growth Des.* **15,** 1553-1558.
